# Structure of a Complete ATP Synthase Dimer Reveals the Molecular Basis of Inner Mitochondrial Membrane Morphology

**DOI:** 10.1016/j.molcel.2016.05.037

**Published:** 2016-08-04

**Authors:** Alexander Hahn, Kristian Parey, Maike Bublitz, Deryck J. Mills, Volker Zickermann, Janet Vonck, Werner Kühlbrandt, Thomas Meier

**Affiliations:** 1 Department of Structural Biology, Max Planck Institute of Biophysics, Max-von-Laue-Str. 3, 60438 Frankfurt am Main, Germany; 2 Institute of Biochemistry, Goethe University Frankfurt, Max-von-Laue-Str. 9, 60438 Frankfurt am Main, Germany; 3 Institute of Biochemistry II, Medical School, Goethe University Frankfurt, Max-von-Laue-Str. 9, 60438 Frankfurt am Main, Germany

**Keywords:** mitochondria, inner membrane morphology, F_1_F_o_-ATP synthase dimer, bioenergetics, membrane protein complex, rotary ATPase mechanism, yeast *Yarrowia lipolytica*, cryoelectron microscopy, X-ray crystallography

## Abstract

We determined the structure of a complete, dimeric F_1_F_o_-ATP synthase from yeast *Yarrowia lipolytica* mitochondria by a combination of cryo-EM and X-ray crystallography. The final structure resolves 58 of the 60 dimer subunits. Horizontal helices of subunit *a* in F_o_ wrap around the *c*-ring rotor, and a total of six vertical helices assigned to subunits *a, b, f, i*, and *8* span the membrane. Subunit *8* (*A6L* in human) is an evolutionary derivative of the bacterial *b* subunit. On the lumenal membrane surface, subunit *f* establishes direct contact between the two monomers. Comparison with a cryo-EM map of the F_1_F_o_ monomer identifies subunits *e* and *g* at the lateral dimer interface. They do not form dimer contacts but enable dimer formation by inducing a strong membrane curvature of ∼100°. Our structure explains the structural basis of cristae formation in mitochondria, a landmark signature of eukaryotic cell morphology.

## Highlights

• Cryo-EM structure of a yeast F_1_F_o_-ATP synthase dimer
• Inhibitor-free X-ray structure of the F_1_ head and rotor complex
• Mechanism of ATP generation by rotary catalysis
• Structural basis of cristae formation in the inner mitochondrial membrane

## Graphical Abstract

**Figure:**
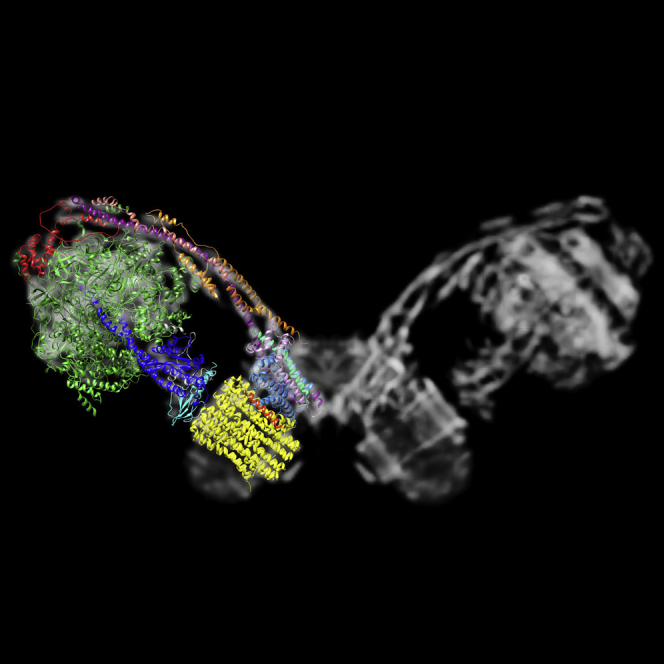

## Teaser

ATP synthases are complex macromolecular machines that supply most of the ATP in cells. Hahn et al. present the structure of a complete ATP synthase dimer, which provides insights into both the mechanism of these nanomotors and how they cause membrane bending to form cristae in the inner mitochondrial membrane.

## Introduction

The mitochondrial F_1_F_o_-ATP synthase produces most of the ATP in the cell by rotary catalysis and plays a crucial role in severe human neurodegenerative disorders (Kucharczyk et al., 2009). The proton motive force (pmf) across the inner membrane drives the *c*-ring rotor in the membrane-embedded F_o_ subcomplex, generating the torque that powers a sequence of conformational changes in the membrane-extrinsic F_1_ subcomplex, resulting in ATP generation (Abrahams et al., 1994, Boyer, 1997, Noji et al., 1997). The F_o_ subcomplex is connected to F_1_ by the central stalk, which transmits torque to the catalytic head, and the peripheral stalk, which acts as a stator to prevent idle rotation of the F_1_ head with the *c*-ring.

Dimers of the ATP synthase shape the inner mitochondrial membrane and mediate cristae formation (Davies et al., 2012, Paumard et al., 2002). The ATP synthase forms rows of V-shaped dimers along the highly curved edges of inner membrane cristae (Strauss et al., 2008). The dimer angle is 86° in yeasts and metazoans, but different in mitochondria of plants (Davies et al., 2011) and algae (Allegretti et al., 2015). Recently, the complete structure of the dimeric mitochondrial ATP synthase of the chlorophyll-less green alga *Polytomella* sp. was reported at 6.2 Å resolution, revealing the unexpected feature of a horizontal four-helix bundle in the *a* subunit of the F_o_ subcomplex (Allegretti et al., 2015). The long horizontal helices are conserved not only in mammalian mitochondria (Zhou et al., 2015) and bacteria (Morales-Rios et al., 2015) but also in the more distantly related V-type and A-type ATPases (Zhao et al., 2015), and are thus a fundamental feature common to all rotary ATPases (Kühlbrandt and Davies, 2016). Together with the *c*-ring rotor, the horizontal helices of subunit *a* create two aqueous half-channels on either side of the membrane (Allegretti et al., 2015, Kühlbrandt and Davies, 2016). The *c* subunits in the rotor ring bind and release protons as the ring rotates through the alternating hydrophobic environment of the lipid bilayer and the aqueous environment of the half-channels (Allegretti et al., 2015, Meier et al., 2011, Meier et al., 2005, Pogoryelov et al., 2010, Symersky et al., 2012), thereby generating the torque for ATP synthesis.

The recently reported structures include the dimeric form of an ATP synthase that has unusual peripheral stalks (Allegretti et al., 2015), and the monomer of the bovine complex (Zhou et al., 2015) as well as a bacterial ATP synthase (Morales-Rios et al., 2015), which both appear to be incomplete. There is currently no structure of an ATP synthase dimer that closely resembles the mammalian complex. Mitochondrial ATP synthases from yeasts have a subunit composition very similar to the mammalian (human) ATP synthase and form the same V-shaped dimers. By a combination of cryoelectron microscopy (cryo-EM) and X-ray crystallography, we have obtained the structure of the complete ATP synthase dimer from the aerobic, genetically accessible yeast *Yarrowia lipolytica*, in which ATP synthase dimers were previously reported (Davies et al., 2011, Nübel et al., 2009). The combined maps resolve 58 of the 60 known protein subunits and the inhibitor protein *IF1*. The structure reveals the previously unknown subunit architecture of the dimer interface in the membrane, thereby providing major new insights into mitochondrial membrane architecture.

## Results

### Isolation and Biochemistry of *Yarrowia lipolytica* ATP Synthase Dimers

ATP synthase dimers from *Y. lipolytica* were purified from dodecylmaltoside (DDM)-solubilized mitochondrial membranes by centrifugation in a digitonin-containing glycerol gradient, followed by anion exchange chromatography. Two-dimensional gel electrophoresis and liquid chromatography-mass spectrometry (LC-MS) indicated that the 2*YL*F_1_F_o_ (*Y. lipolytica* ATP synthase dimer) fraction contained all ATP synthase subunits, including *e*, *g*, and *k*, which are known as dimer specific (Arnold et al., 1998) (Figure S1; Table S1, available online). The DDM-purified monomeric *Y. lipolytica* ATP synthase (1*YL*F_1_F_o_) lacks subunits *e*, *g*, and *k*. The ATP hydrolysis activity of both 1*YL*F_1_F_o_ and 2*YL*F_1_F_o_ is ∼2.25 U/mg. F_o_ is coupled to 95% and 75%, respectively, as determined by oligomycin inhibition. The lower percentage of coupled complexes in 2*YL*F_1_F_o_ is most likely due to free F_1_ subcomplexes and detergent in the dimer preparation (Figure S1A). The similarly high activities of 2*YL*F_1_F_o_ and 1*YL*F_1_F_o_ indicate that the two ATP synthase monomers within the dimer operate independently in ATP hydrolysis mode.

### F_1_*c*_10_ Crystal Structure

**Figure 1:**
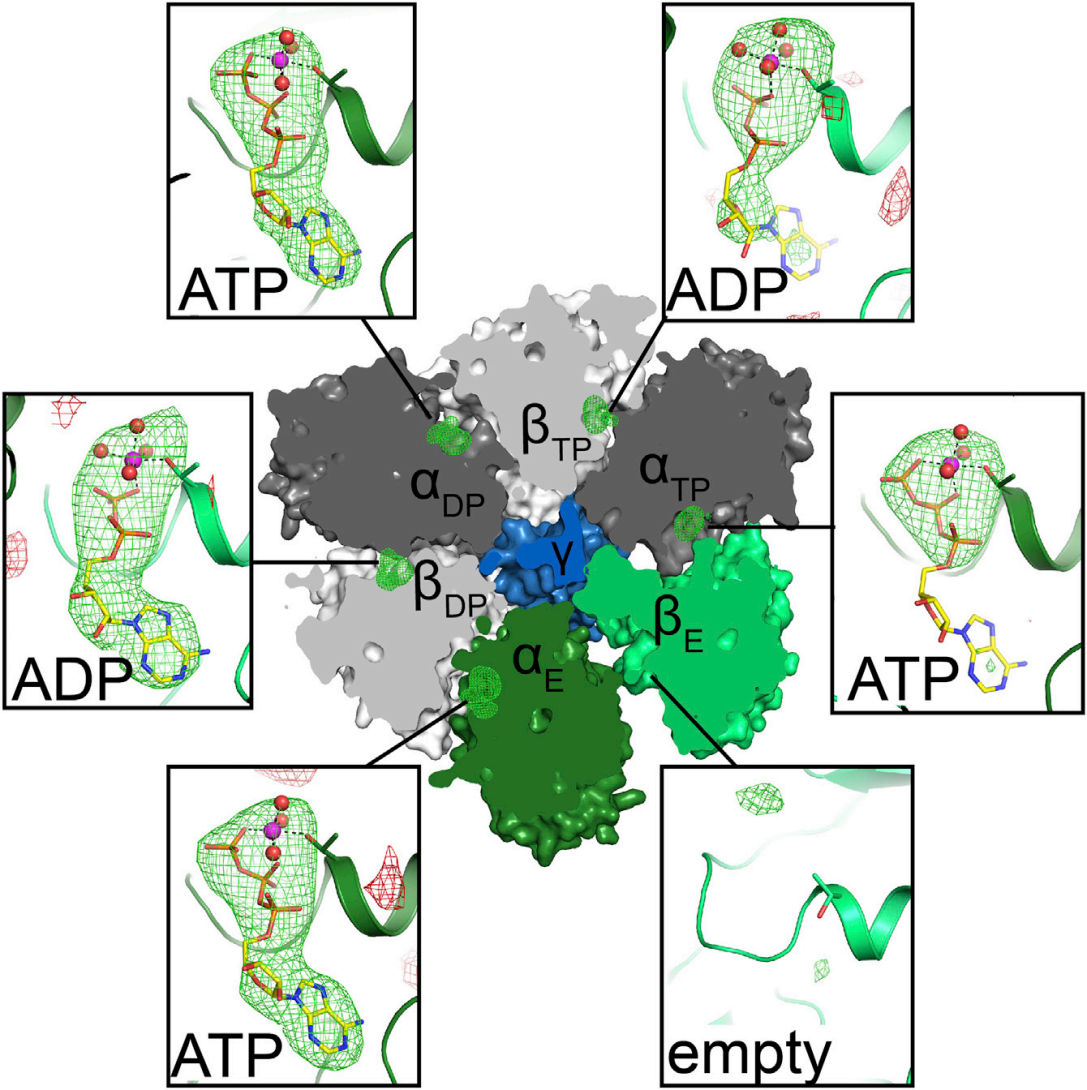
Nucleotide Binding Sites in *Yarrowia lipolytica* F_1_*c*_10_ Cross section through the F_1_ X-ray structure shows the six nucleotide binding sites at the α/β subunit interfaces viewed from the matrix. Subunits α (dark green, dark gray) and β (light green, light gray) are arranged around the central γ subunit (blue). Green and red mesh indicates unbiased *m*F_obs_-*D*F_calc_ nucleotide difference densities contoured at 3.0σ and −3.0σ, respectively. Boxed, close-up views of Walker A nucleotide binding motifs (cartoon) with αThr202, βThr195, and nucleotides in stick representation. Mg^2+^ ions with coordinated water molecules are shown as spheres. Atoms of C, N, O, P, and Mg are colored yellow, blue, red, orange, and magenta, respectively. Positive difference densities match Mg·ATP [·3 H_2_O] in all three α sites, and Mg·ADP[·4 H_2_O] in β_DP_ and β_TP_. The β_E_ site is empty. The conformational flexibility of the β_E_ subunit is a possible cause of the weaker adenosine density of the ATP in the adjacent α_TP_ site. See Figure S3 for further difference maps after modeling various nucleotides in the α and β sites.

Crystals of the *YL*F_1_*c*_10_ subcomplex were obtained from the 1*YL*F_1_F_o_ complex. Whereas previous crystallographic studies of similar complexes (Giraud et al., 2012, Pagadala et al., 2011, Stock et al., 1999) used an excess of nucleotide substrates or inhibitors to trap functional states, we crystallized *YL*F_1_*c*_10_ without any such additives to ensure similar conditions for crystallography and cryo-EM. The 3.5 Å X-ray structure of *YL*F_1_*c*_10_ (Table 1; Figures S2A–S2D) reveals that all three non-catalytic α subunits bind Mg·ATP in their nucleotide sites. Of the three catalytic β subunits, one is empty (β_E_), while both the β_DP_ and β_TP_ sites (Abrahams et al., 1994) contain Mg·ADP (Figures 1 and S2E).

### Cryo-EM Structure of the *Yarrowia lipolytica* ATP Synthase Dimer

**Figure 2:**
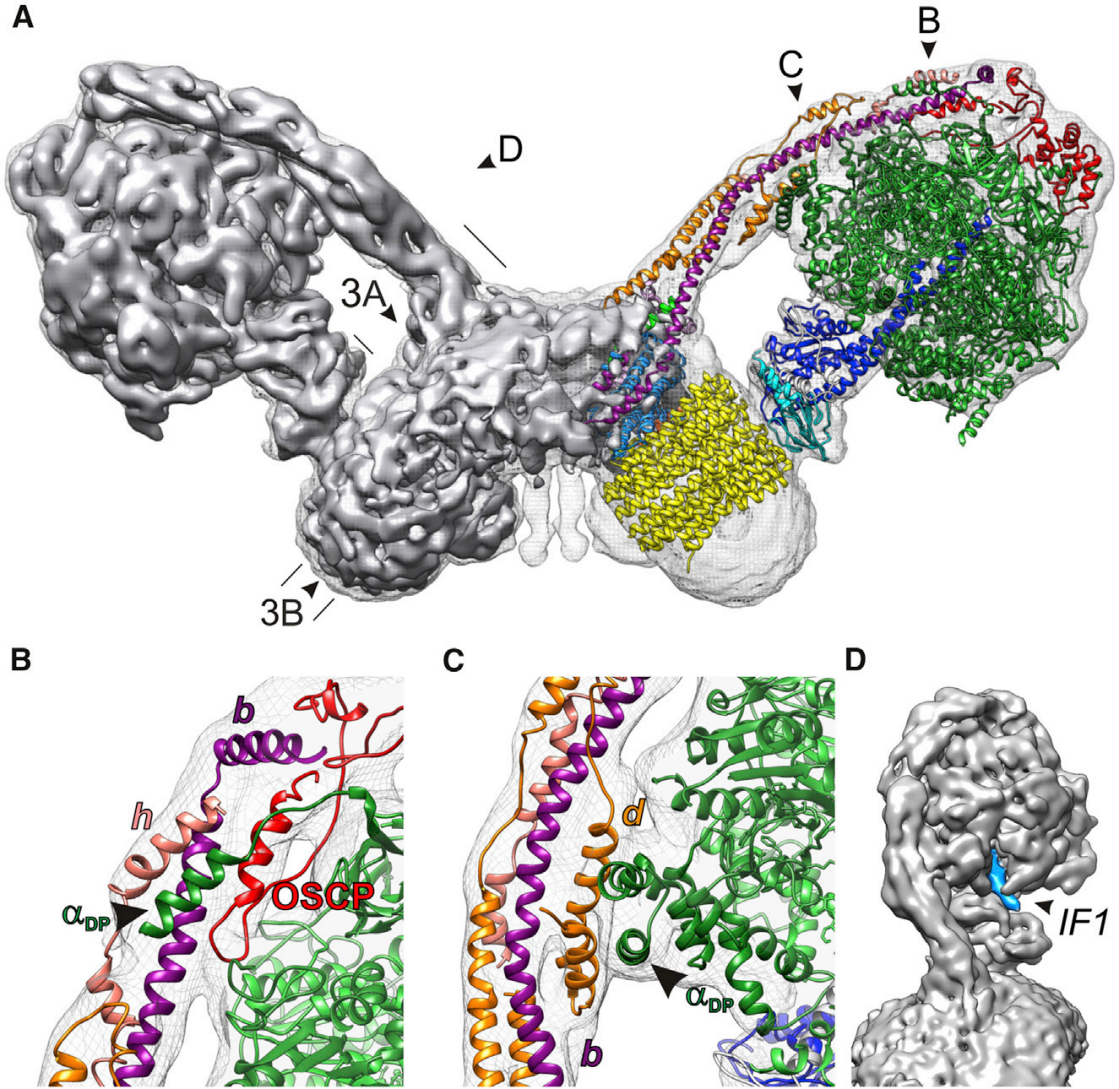
Cryo-EM Structure of the *Yarrowia lipolytica* F_1_F_o_-ATP Synthase Dimer (A) Side view of the map (gray surface and volume). The monomer on the right was fitted in Coot (Emsley et al., 2010) with the X-ray structure of the *Y. lipolytica* F_1_*c*_10_ complex and homology models of peripheral stalk subunits based on atomic models from the *B. taurus* outer stalk structures (PDB: 2WSS and 2CLY) (Figures 1 and S2E). Cross sections shown in Figures 3A and 3B and viewing directions for (B)–(D) are indicated. (B and C) Detailed views of peripheral stalk subunit interactions as indicated in (A). (B) Upper and (C) lower section. Dark and light green, α and β subunits, respectively; light blue, subunit a; yellow, *c*_10_ ring; blue, subunit γ; cyan, subunit δ; light gray, subunit ε; purple, subunit *b*; orange, subunit *d*; salmon, subunit *h*; red, *OSCP*. (D) The intrinsic inhibitor protein *IF1* (light blue) binds in the α/β_DP_ site proximal to the peripheral stalk. The overall map resolution of 7.8 Å in (A) improved upon masking to 6.9 Å for the F_1_ complex (B–D). Movie S1 is a video of the rotating complete ATP synthase dimer.

We determined the structure of the 2*YL*F_1_F_o_ by single-particle cryo-EM (Figure 2A). After 2D and 3D classification, 38,679 particles were selected for reconstruction of a 3D map with C2 symmetry imposed. The central stalks of the two monomers include an angle of ∼100°. Masking one monomer in the dimer during 3D refinement improved the resolution to 6.9 Å for the F_1_ subcomplex and masking the F_o_ dimer improved it to 6.2 Å, as determined by gold-standard Fourier shell correlation (Figure S4). The long helices in the peripheral stalks and the F_o_ part of the stator are the best-resolved features (Movie S1). The resolution of the F_1_*c*_10_ subcomplex in the cryo-EM map is slightly lower, due to minor variations in the dimer angle (Figures S3D and S3E) and to differences in rotational position of the rotor assembly. Further classification revealed that the position of the central stalk varies independently in both monomers (Figures S3F and S3G), confirming that the two ATP synthase assemblies in the dimer function independently, as already suggested by the similar ATPase hydrolysis activities of 1*YL*F_1_F_o_ and 2*YL*F_1_F_o_.

Classification of the same dataset with one monomer in the dimer masked enabled us to distinguish three different rotational states of the F_1_ head assembly, with two out of three positions favored, in which the positions of the central stalk differ by ∼120° or 240° (Figures S3H and S3I). Interaction with the central stalk affects the nucleotide binding domains and C-terminal domains of the β subunits (Figure S3I). The three conformations show the three “Boyer states,” open, loose, and tight (Boyer, 1997), of the *Y. lipolytica* complex as seen in the *YL*F_1_*c*_10_ crystal structure (Movies S2 and S3), similar to the crystal structure of the bovine F_1_ complex (Abrahams et al., 1994). The three states are trapped in energy wells, which stall the rotor in defined positions upon dissipation of the pmf by the membrane-solubilizing detergent.

In the most populated class (45% of the particles; subclass 2 in Figures S3H and S3I), a rod-like density protrudes from the α_DP_β_DP_ pair close to the peripheral stalk (Figure 2D). This density superposes precisely on the inhibitor *IF1* in an X-ray structure of the bovine mitochondrial F_1_F_o_-ATP synthase with *IF1* bound (Gledhill et al., 2007). The presence of *IF1* in ATP synthases prepared from large-scale yeast fermentations is not unexpected, as oxygen concentration of these cultures can decrease, which reduces the matrix pH and triggers *IF1* binding, as observed with yeast grown on non-fermentable substrates (Satre et al., 1975). The fact that *IF1* is found in only one of the three classes is, however, surprising.

### Peripheral Stalk

The peripheral stalk consists of several long, well-resolved α helices, which were traced without ambiguity (Figures 2A–2C). Homology models based on crystal structures of the bovine subunits *b*, *d*, and *OSCP* (Dickson et al., 2006, Rees et al., 2009) were fitted to the soluble sector of the *Y. lipolytica* peripheral stalk, which has the same subunit composition (Table S1). Subunit *h* has only 20% sequence identity to the equivalent bovine *F6* (Fujikawa et al., 2015), accounting for the observed structural differences. The overall curvature of the peripheral stalk differs from that in the bovine crystal structure, but resembles that in the cryo-EM map of the monomeric bovine complex (Baker et al., 2012), suggesting that crystal contacts affect stalk curvature. As in the bovine complex (Zhou et al., 2015), helices 1 and 5 of *OSCP* on the F_1_ head are in contact with the N terminus of α_E_ (Rees et al., 2009). Two further close contacts are found at the N terminus of α_TP_, which interacts with helices 4 and 5 of *OSCP*, and at the N terminus of α_DP_, which intercalates between the peripheral stalk helices. The N terminus of this α subunit forms a previously unrecognized four-helix bundle with *b*, *h*, and the C terminus of *OSCP*, which positions the F_1_ head and bonds it to the peripheral stalk (Figure 2B). The contacts in this interaction are mainly hydrophobic, except for those mediated by the conserved residues αGlu33 and αArg41. The *d* subunit interacts with the C terminus of the α_DP_ subunit, displacing it toward the peripheral stalk by 5 Å relative to the *YL*F_1_*c*_10_ X-ray structure (Figure 2C). Below the F_1_ head, peripheral stalk subunits *d* and *b* bend toward the central stalk. The density of subunit *b*, which is thought to have two trans-membrane helices at its N terminus (Figure S4), continues without interruption into the membrane.

### Helix Assignment in the F_o_ Stator

**Figure 3:**
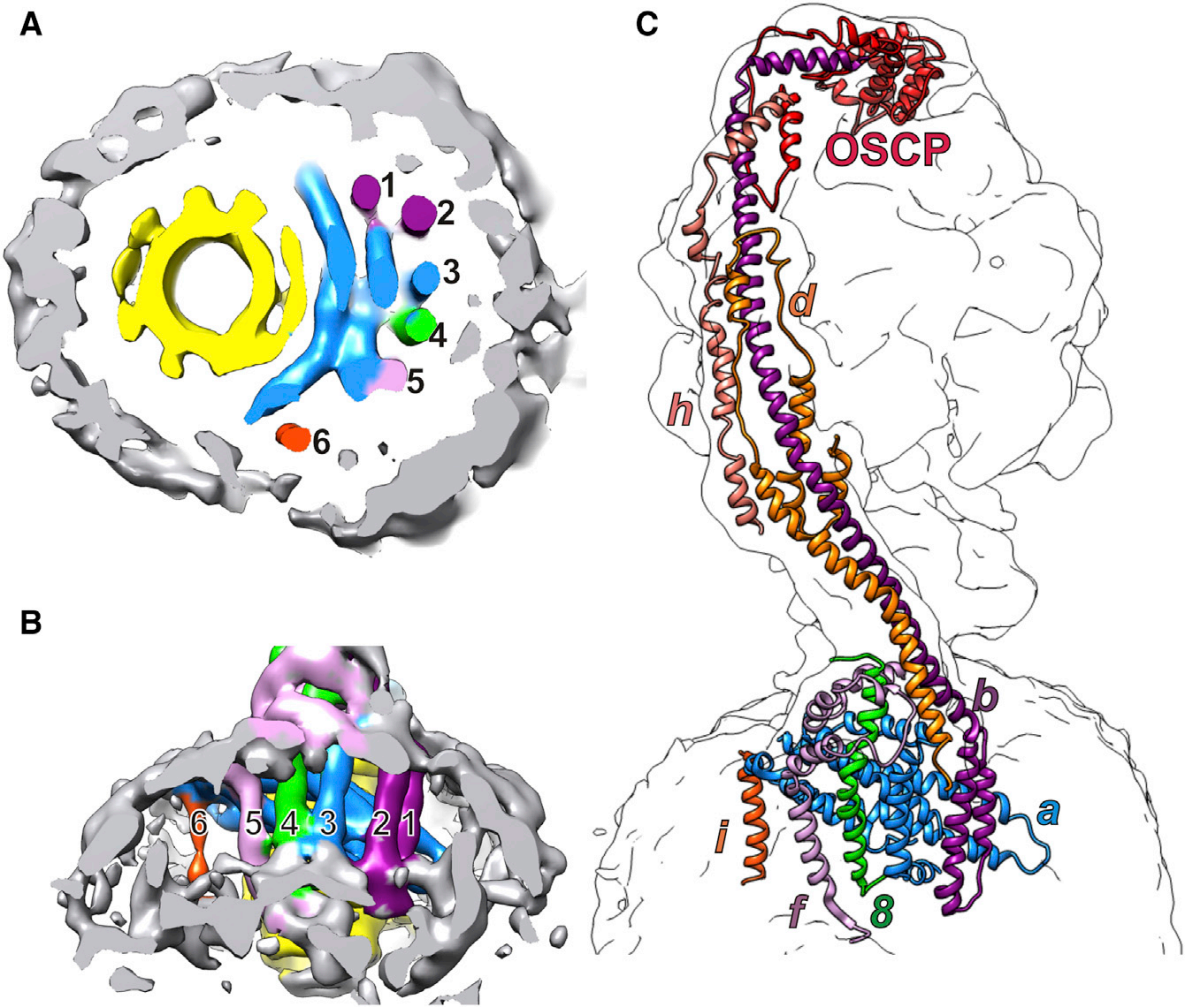
Assignment of the *Yarrowia lipolytica* F_1_F_o_-ATP Synthase Stator Region (A and B) Cross sections through the F_o_ stator region as indicated in Figure 2A. (A) View from the matrix and (B) from the membrane. Four horizontal and six vertical helix densities (labeled 1–6) next to the *c*_10_ ring rotor (yellow) in the detergent micelle (gray) were assigned to stator subunits. Blue, subunit *a*; purple, subunit *b*; green, subunit *8*; lavender, subunit *f*; dark orange, subunit *i*. (C) Overview of peripheral stalk and stator subunits in the cryo-EM map. The overall map resolution of 7.8 Å in Figure 2A improved upon masking to 6.2 Å for the F_o_ dimer (A and B) and 6.9 Å for the F_1_ complex (C).

The *Y. lipolytica* F_o_ stator subcomplex comprises the eight membrane protein subunits *a*, *b*, *e*, *f*, *g*, *i*, *k*, and *8*. The F_o_ part of each monomer contains ten well-defined α-helical densities enveloped by a detergent micelle that features the characteristic ∼90° dimer membrane curvature (Davies et al., 2012) (Figure 2A). Six of these densities indicate trans-membrane α helices, numbered 1–6 in Figures 3A and 3B and assigned in Figure 3C. The loops connecting the helices are, for the most part, not visible at this resolution, but the helix segments can be identified on the basis of sequence comparison, secondary structure predictions, proximity, and known helix topology.

#### Subunit b

Helix 1 is the continuation of the peripheral stalk subunit *b* and is thus the second trans-membrane helix of *b.* Helix 2 is close to it and is the most likely candidate for the first trans-membrane helix of this subunit. The second-nearest helix 3 is too far for the short, six-residue loop connecting the two trans-membrane helices of subunit *b* (Figure S4A).

#### Subunit a

Sequence alignment of subunit *a* indicates a consistent pattern of seven characteristic consecutive protein regions (Figure S5A): (i) the hydrophilic N terminus; (ii) a ∼20 residue hydrophobic stretch indicative of a trans-membrane helix; (iii) a region rich in hydrophilic and polar residues, prone to form an amphipathic helix (Figure S5B); (iv) two hydrophobic sequences separated by charged or polar side chains; (v) a region with several positively charged residues followed by (vi) a proline-rich region; and finally (vii) an extensive hydrophobic stretch with interspersed, highly conserved charged and polar residues.

**Figure 4:**
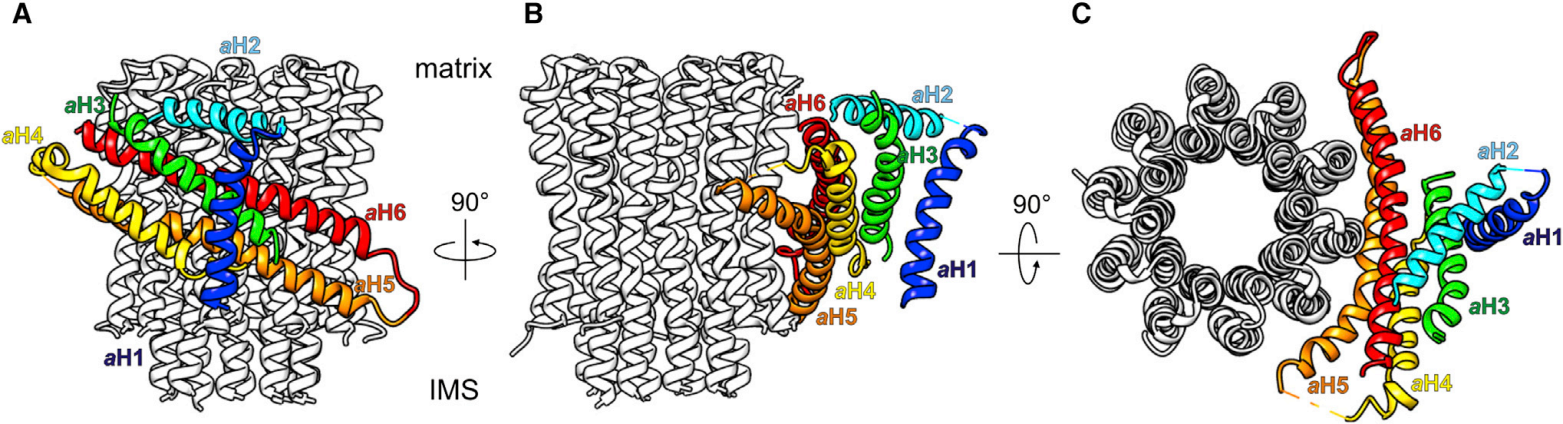
Subunit *a* View (A) from the dimer interface, (B) along the *a*/*c* interface, and (C) from the matrix. The hydrophilic N terminus on the IMS side (region (i) in the text) is not resolved. Helix *a*H1 (blue, region (ii)) is the only vertical trans-membrane helix of subunit *a*. The amphipathic helix *a*H2 (cyan, region (iii)) runs along the matrix membrane surface. The membrane-intrinsic helices *a*H3 (green) and *a*H4 (yellow) of region (iv) form a hairpin. Regions (v) and (vi) are the unresolved connection between helices *a*H4 and *a*H5. The membrane-intrinsic helices *a*H5 (orange) and *a*H6 (red) form a second, longer hairpin (region (vii)), tilted by 20°–25° relative to the membrane plane. *a*H5 follows the curve of the *c*-ring (light gray) closely. *a*H5 and *a*H6 are ∼70 Å long and in contact with 3–4 *c* subunits, while the helices in the distal hairpin, *a*H3 and *a*H4, are ∼45 and ∼35 Å long; neither is in direct contact with the *c*-ring. The *a*H4/*a*H5 hairpin loop is on the IMS side, while the C terminus is exposed on the matrix surface. All six *a* subunit helices are highly conserved (Figure S5).

We can assign region (iii), the amphipathic helix *a*H2, to the straight helix density on the matrix side just above the horizontal four-helix bundle (Figure 4). Region (ii), the trans-membrane helix of subunit *a*, which we refer to as *a*H1, would thus be helix density 3 in the map (Figures 3 and 4). The N-terminal region (i) of subunit *a* is small, is without clear predicted secondary structure, and has no discernible map density. Regions (iv) to (vii) are assigned to the two membrane-intrinsic helix hairpins of subunit *a* (which we refer to as *a*H3 to *a*H6) on the basis of their striking similarity to the same feature in the *Polytomella* dimer map (Allegretti et al., 2015). The assignment of the two shorter helices as *a*H3 and *a*H4 follows from their proximity to the amphipathic helix *a*H2 (Figure 4). The non-helical regions (v) and (vi) link the two helix hairpins, but only limited density is visible for them in the map. We assign the longest helix in the four-helix bundle, which follows the curve of the *c*-ring closely, to *a*H5 in the first half of region (vii), and the second helix in this hairpin to helix *a*H6 in the C-terminal half of this region (Figure 4). Helix *a*H5 contains the essential Arg182, which interacts with the protonatable *c*-ring glutamate (Cain, 2000, Eya et al., 1991, Lightowlers et al., 1987). Our assignment places this residue and a series of conserved charged or polar residues in the long horizontal hairpin at the subunit *a*/*c* interface (see Discussion). Our *a* subunit assignment is fully consistent with that of the bovine (Zhou et al., 2015) and *Paracoccus* ATP synthase (Morales-Rios et al., 2015), but the order of helices *a*H5 and *a*H6 with respect to the *Polytomella* assignment (Allegretti et al., 2015) is reversed.

#### Subunit 8

Helix 4 (green in Figure 3) has a short matrix extension with a slight kink toward the *c*-ring. We assign this density to the small, 48-residue subunit *8* (Figure S4B). Subunit *8* has a conserved N-terminal MPQL motif located in the intermembrane space (IMS) (Stephens et al., 2000), followed by a trans-membrane helix, terminated in yeasts by the conserved Pro33, and a short hydrophilic C-terminal stretch. This sequence fits the density well, with Pro33 at the kink. The trans-membrane helix of subunit *8* has a short connecting density in the IMS toward the *c*-ring and below the first helix hairpin of the *a* subunit, which accommodates the conserved MPQL motif. Thus, the N terminus of subunit *8* appears to anchor the horizontal four-helix bundle of subunit *a* in its position within the F_o_ assembly.

The longer mammalian subunit *8* has been shown to interact at its C terminus with the peripheral stalk subunits *b* and *d* (Lee et al., 2015); in plant mitochondria, subunit *8* is as long as a typical *b* subunit. This subunit was thought to have no prokaryotic equivalent (Lee et al., 2015, Stephens et al., 2003), but comparison with the *b* subunit of α-proteobacteria, which share a common ancestor with mitochondria, strikingly reveals the same N-terminal MPQL motif. Therefore, the mitochondrial subunit *8* derives from one of the two *b* subunits of its bacterial ancestor and is truncated in the mammalian and fungal lines. Subunit *8* is one of the few mitochondrially encoded ATP synthase components in *Y. lipolytica*, together with the *a* and *c* subunits (Kerscher et al., 2001), consistent with its bacterial origin.

#### Subunit f

Helix 5 (lavender in Figure 3) is the most likely candidate for the nuclear-encoded trans-membrane subunit *f*. In yeast, this 100-residue subunit has a hydrophilic N-terminal domain on the matrix side and a predicted C-terminal trans-membrane helix (Figure S4C). Three curved densities at the base of the peripheral stalk (Figure 3B) that surround the matrix extension of subunit *8* are assigned to the N terminus of subunit *f*. The sharp changes in direction between the densities assigned to this subunit are consistent with the positions of conserved prolines in the *f* subunit sequence alignment.

#### Subunit i

Finally, the density of helix 6 (orange in Figures 3A and 3B) is weaker than the others. Based on its position next to the *a* subunit, we assign it to the yeast-specific, non-essential subunit *i*, which is present in both the monomer and the dimer in *Y. lipolytica* (Table S1) and has been shown to interact with subunits *a*, *f*, *d*, and *g* (Paumard et al., 2000).

Our assignments are fully consistent with all previously reported chemical crosslinking results of ATP synthases from yeasts, metazoans, and bacteria (DeLeon-Rangel et al., 2013, Jiang and Fillingame, 1998, Schwem and Fillingame, 2006, Stephens et al., 2003) (Figure S6).

#### Subunits e and g at the Dimer Interface

**Figure 5:**
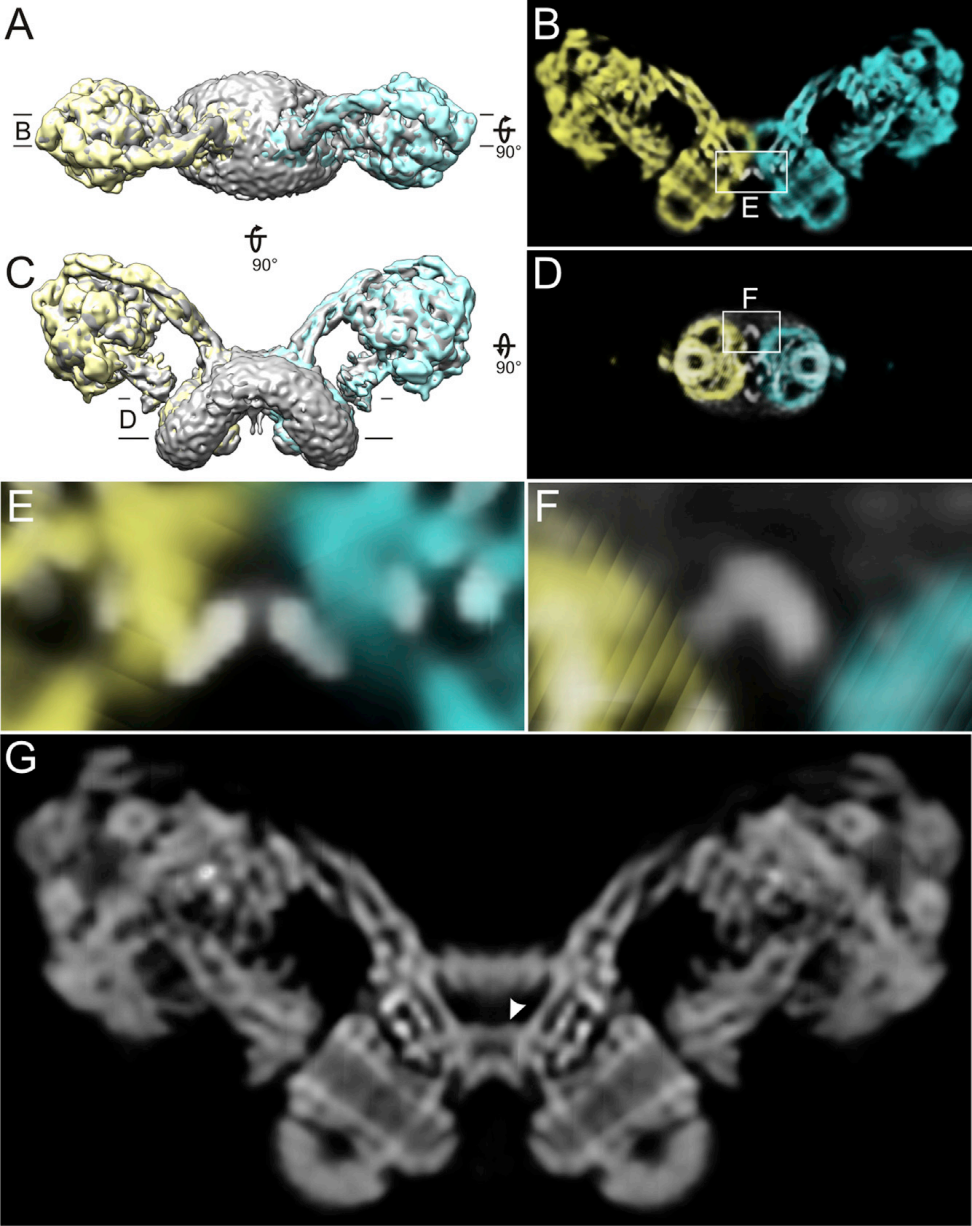
Dimer Interface (A) Matrix view of the *Y. lipolytica* ATP synthase cryo-EM dimer map (gray) with superposed monomer maps (yellow and blue). (B) Vertical slice through monomer maps as indicated in (A). Dimer contacts are mediated by protein densities outside the detergent micelle of the monomer (boxed). (C) Side view of superposed maps in (A). (D) Horizontal slice through the dimer interface as indicated in (C). The white density (boxed) belongs to membrane subunits present in the dimer, but not in the monomer. LC-MS analysis of monomer and dimer subunit composition identifies these subunits as *e* and *g* (Table S1). (E) Detailed view of the lumenal dimer contacts as indicated in (B). (F) Detailed view of the density assigned to the dimer-specific subunits *e* and *g* as indicated in (D). (G) Central slice of the dimer. A sheet-like density (arrowhead) connecting subunits *a* and *8* may be the yeast-specific subunit *k*, the only unassigned protein in the dimer map.

We collected a cryo-EM dataset of 1*YL*F_1_F_o_ and generated a 3D map of the monomeric *Y. lipolytica* ATP synthase at 8.4 Å resolution (Figure 5). Unlike the dimer, 1*YL*F_1_F_o_ does not contain the dimer-specific subunits *e*, *g*, and *k* (Table S1). The bovine monomer has subunits *e* and *g*, but not *k* (Baker et al., 2012). A comparison of the 3D map volumes therefore reveals the location of *e* and *g* in the dimer map (Figures 5D and 5F). They occupy a roughly triangular density on the dimer interface next to the N-terminal trans-membrane helices of subunit *b*, with a narrow extension that protrudes ∼40 Å into the IMS. This density is similar to the *e*/*g* density assigned in the bovine monomer (Zhou et al., 2015), but the orientation of the IMS extension is different (see below).

Subunit *e* is predicted to have an N-terminal trans-membrane helix with a conserved, essential GxxxG motif, a signature of helix-helix interaction (Arselin et al., 2003), and a hydrophilic C terminus that would account for the IMS extension. The *g* subunit can be crosslinked to the N terminus of *b* in the matrix (Soubannier et al., 1999). Deleting the first trans-membrane helix of *b* results in the loss of *g* and dissociation of the dimer (Soubannier et al., 2002), indicating that *g* contributes to dimer stability.

**Figure 6:**
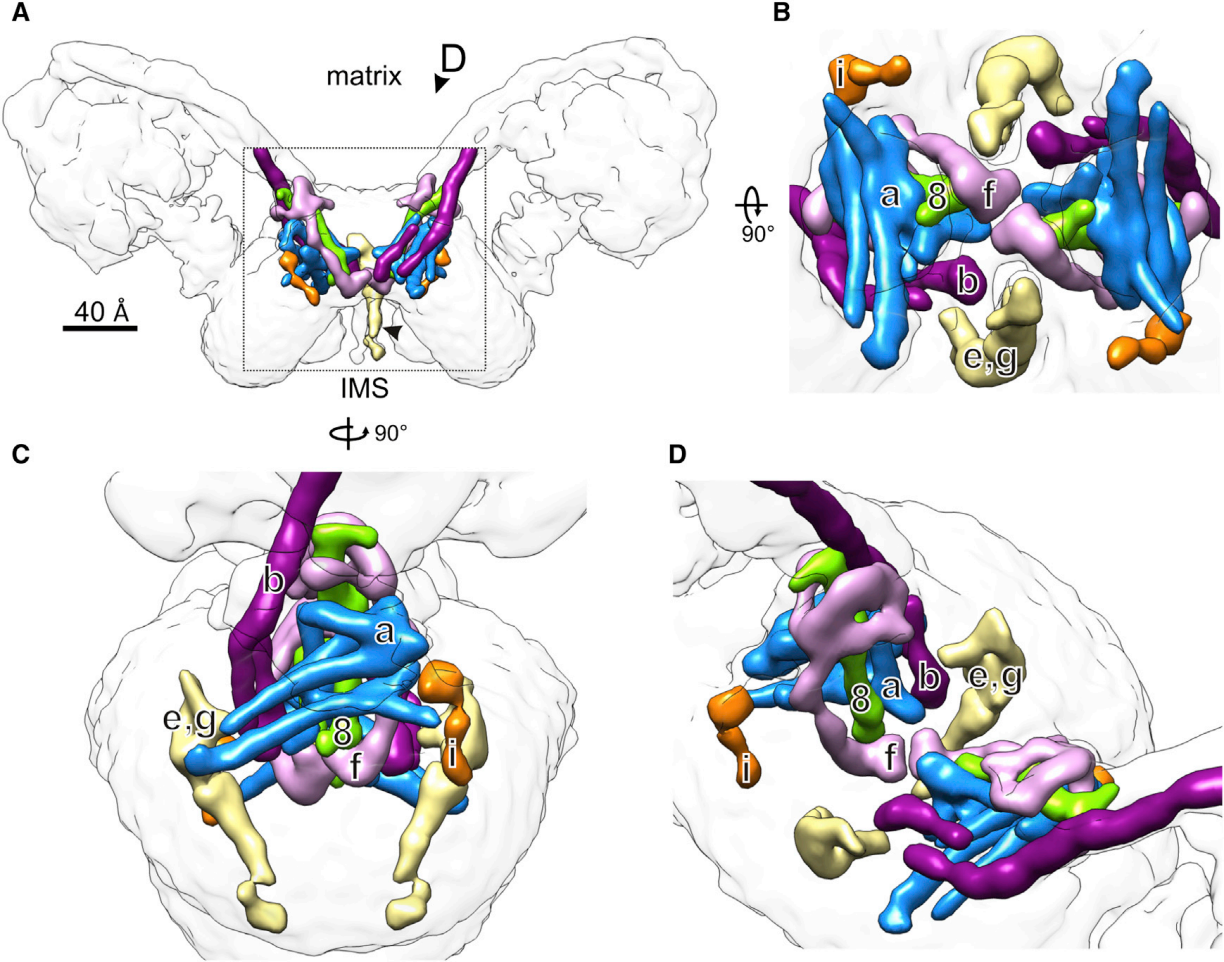
The F_o_ Stator Membrane protein densities in the two F_o_ stator complexes of the *Y. lipolytica* ATP synthase dimer. Subunits *a*, *b*, *f*, *i*, and *8* are colored as in Figure 2. Subunits *e* and *g* are ivory. (A) Side view of the dimer interface. The IMS extensions of subunit *e* (arrowhead) were segmented at a lower contour level. F_o_ subunits of the two ATP synthase monomers interact on the IMS side. A 40 Å gap on the matrix side contains lipid or detergent. (B) View from the IMS. The C-terminal segment of subunit *f* mediates a direct protein contact between the two monomers in the dimer. Densities on either side of the protein contact are assigned to subunits *e* and *g* (see Figure 6). (C) View from the *c*-ring. (D) Oblique view in the direction indicated in (A). For an evaluation of Cys-Cys crosslinks, see Figure S6 and Table S3.

Subunit *g* consists of an N-terminal matrix domain and a predicted C-terminal trans-membrane helix that likewise contains a conserved GxxxG motif. Subunits *e* and *g* may thus form a tight heterodimer in the membrane via their GxxxG motifs. The helices in such a tight heterodimer would not be resolved at 6.2 Å, like the inner helices of the *c*-ring, which are known to interact through such motifs (Vonck et al., 2002). There is no contact between the *e*/*g* density of one monomer to any subunit of the other, so *e* and *g* do not participate directly in dimer formation. Side views of the bovine and *Y. lipolytica* maps (Figure S7A) indicate that each *e*/*g* heterodimer bends the membrane by ∼50°, resulting in the ∼100° kink observed in the dimer. The most prominent direct dimer contact is formed by the C-terminal domain of subunit *f* (Figure 6). The C terminus of subunit *f* contains conserved charged and polar residues that would mediate this interaction (Figure S3C). The membrane curvature induced by subunits *e* and *g* appears to be necessary to position the C-terminal domains of the *f* subunits in both monomers for interaction across the interface, resulting in dimer formation.

## Discussion

### Rotational F_1_ States

A detailed comparison of the *YL*F_1_*c*_10_ crystal structure to the bovine (Abrahams et al., 1994) and *Saccharomyces cerevisiae* (Kabaleeswaran et al., 2006) F_1_ and F_1_*c*_10_ complexes (Figure S8) reveals that the three conformational states of the corresponding α/β heterodimers are very similar in the two yeast species, with root-mean-square deviation (RMSD) values below 1.7 Å, while nucleotide binding and C-terminal regions of the bovine β subunits differ (Table S2; Movies S4 and S5). In *YL*F_1_*c*_10_ both the β_E_ and the β_DP_ site are more open than in the bovine complex, while their β_TP_ sites are similar. Overall, the three β subunits resemble one another more closely in *Y. lipolytica* than in the bovine and *S. cerevisiae* complexes (Figure S8B).

Aligning the γ subunits in all F_1_ X-ray structures and comparing the relative positions of the conserved P loop in the β_DP_ subunit (Figure S8A), we find that the *YL*F_1_*c*_10_ P loop is shifted to a position that, in bovine F_1_ (Rees et al., 2012), would indicate a post-hydrolysis or pre-product release state. The post-hydrolysis position of the γ subunit in *YL*F_1_*c*_10_ agrees with the presence of bound Mg·ADP in the catalytic β_DP_ and β_TP_ sites. Since *YL*F_1_*c*_10_ was crystallized without added nucleotides, the ADP originates from ATP hydrolysis during isolation or crystallization (Abrahams et al., 1996, Gledhill et al., 2007). The fact that ADP is present in the *Y. lipolytica* β_DP_ at this late stage of hydrolysis without addition of nucleotide-stabilizing azide (Bowler et al., 2006) is surprising, as ADP was not found in the binding sites of other F_1_ complexes crystallized under similar conditions (Bianchet et al., 1998, Stocker et al., 2007). This might indicate a higher nucleotide affinity of the *Y. lipolytica* β_DP_ site. In contrast, phosphate (P_i_) was not detected in the β_DP_ and β_TP_ sites, in line with a possible alternative leaving route for P_i_, as described for *S. cerevisiae* F_1_ (Kabaleeswaran et al., 2006).

We found the inhibitor protein *IF1* bound to one of the three different rotational F_1_ states in the cryo-EM maps, but not in the *YL*F_1_*c*_10_ X-ray structure, indicating that it was lost during crystallization. By contrast, *IF1* was present in all seven rotary states in the cryo-EM maps of monomeric bovine F_1_F_o_-ATP synthase (Zhou et al., 2015), as would be expected since the complex was purified by *IF1* affinity chromatography. Apart from the absence of *IF1* in the β_DP_ region (Figure 2D), the 3.5 Å *YL*F_1_*c*_10_ crystal structure matches the dimer map closely (Figures 2A, S3H, and S3I). Like the X-ray structure, the cryo-EM map therefore shows a post-hydrolysis state.

### Structure of the F_o_ Stator

The subunit *a* structure is remarkably conserved in F_1_F_o_-ATP synthases. Densities for all six helices of our *Y. lipolytica* structure are also present in the same orientations in the cryo-EM map of the bovine heart monomer (Zhou et al., 2015), while four helices are present in the recent 4 Å X-ray structure of the bacterial complex (Morales-Rios et al., 2015) (Figure S7B). The *Polytomella* cryo-EM map (Allegretti et al., 2015) has elements that correspond to each of the six *a* subunit helices in *Y. lipolytica*, even though the polypeptide sequences diverge. The other *Polytomella* stator subunits do not resemble those of yeasts, metazoans, or bacteria.

The cryo-EM map of the bovine monomer shows four trans-membrane helices, two of which were identified as belonging to the peripheral stalk subunit *b*, and one each to subunits *a* and *A6L* (subunit *8* in fungi) (Zhou et al., 2015). All helices superpose well on our map and the assignment agrees with ours, except that there is no density for the *f* subunit in the bovine map (Zhou et al., 2015). Although the bovine complex prepared according to Runswick et al. (Runswick et al., 2013) should contain the *f* subunit, this subunit was not identified by Zhou et al. Subunit *f* may have dissociated during isolation of the bovine monomer, suggesting that it is not firmly attached. As the *f* subunit is responsible for direct dimer contacts in our assignment (Figure 6), its dissociation from the bovine complex may explain why dimers from mammalian mitochondria are, in our experience, less stable.

The X-ray structure of the bacterial F_1_F_o_ complex from *Paracoccus denitrificans* (Morales-Rios et al., 2015) shows only two of the trans-membrane helices in the F_o_ stator, which superpose well on the trans-membrane helix of subunit *8* and *a*H1 in the *Y. lipolytica* dimer (panel (iv) in Figure S7B). Therefore, these helix densities, which were unassigned in the *Paracoccus* map, belong to one of the two bacterial *b* subunits and the trans-membrane helix of subunit *a* (*a*H1), lending strong support to our conclusion that mitochondrial subunit *8* derives from a bacterial *b* subunit. Surprisingly, the trans-membrane helix of the second *b* subunit seems to be completely absent in the *Paracoccus* structure, indicating that it is flexible or disordered in the 4 Å X-ray map.

### Proton Translocation through F_o_

**Figure 7:**
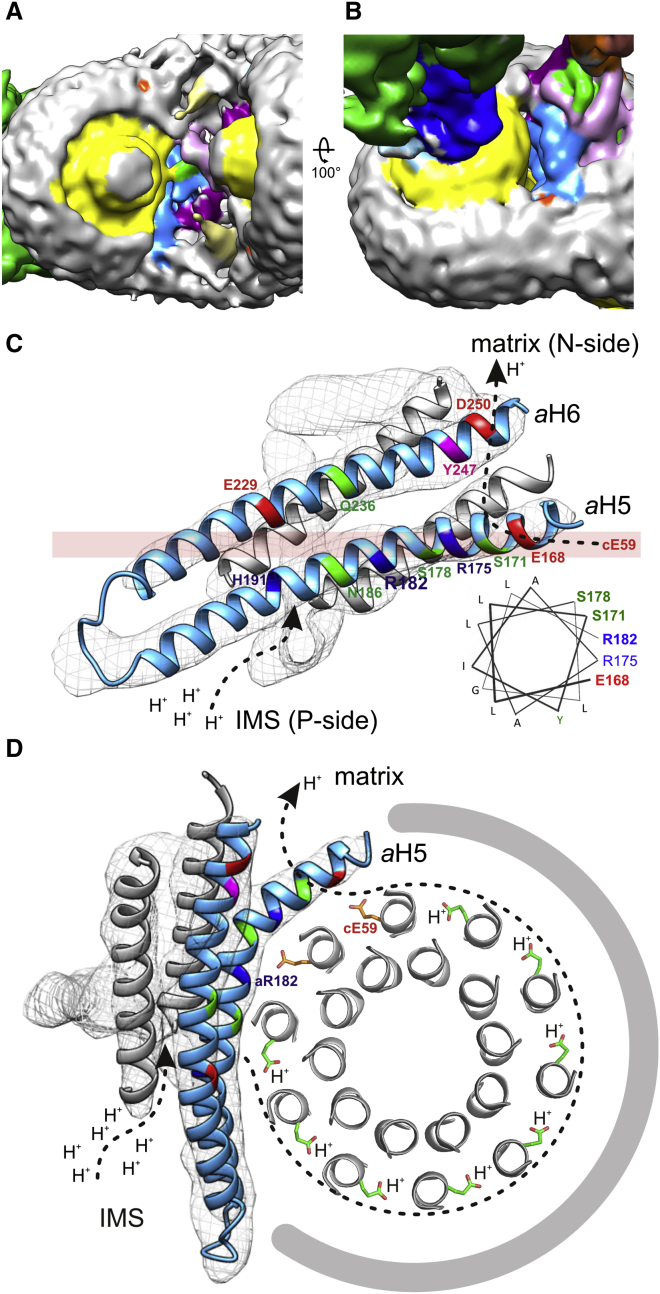
Ion Translocation through F_o_ (A) IMS surface of the *Y. lipolytica* dimer. Subunits are color coded as in Figure 2. The *c*-ring and the dimer contact region are solvent exposed. *a*H5 of subunit *a* (light blue) is visible from the IMS side. The central plug on the *c*-rings consists of lipid or detergent (Meier et al., 2001). (B) Oblique view from the matrix shows the gap between the *c*-ring (yellow) and subunit *a* (light blue). (C) Fitted *a*H5 and *a*H6 hairpin of subunit *a* next to the *c*-ring rotor with modeled positions of conserved positive (blue), negative (red), and polar residues (green). The helical wheel projection (inset) indicates alternating polar, charged, and hydrophobic residues at the start of *a*H5, with polar or charged residues oriented toward the *c*-ring. The transparent pink bar indicates the level at which the protonated *c*-ring glutamates rotate past the strictly conserved *a*Arg182. (D) Section through the *c*_10_-ring (gray) and subunit *a* (gray mesh) with a cartoon model (blue) of the *c*-ring at the level of protonated glutamates in the locked (green) or open (orange) conformation (Pogoryelov et al., 2010, Symersky et al., 2012). *a*H6 peels away from the *c*-ring, accounting for the observed gap at the *a*/*c* interface. Dashed arrows in (C) and (D) indicate the proton pathway from the IMS (P side) to the matrix (N side), as proposed for the *Polytomella* ATP synthase (Allegretti et al., 2015).

Ion translocation through F_o_ is mediated by the *a* subunit and the *c*-ring (Figure 7). A number of high-resolution X-ray structures of ATPase *c*- or *K*-rings have shown that the conserved glutamate residues in the *c* subunits lock the protons (or Na^+^) in the hydrophobic environment of the lipid bilayer (Meier et al., 2005, Murata et al., 2005, Pogoryelov et al., 2009) but open to release the ions in a hydrophilic environment (Mizutani et al., 2011, Pogoryelov et al., 2010, Symersky et al., 2012). The recent cryo-EM structures of the *Polytomella* ATP synthase and *S. cerevisiae* V-type ATPase indicate two aqueous half-channels at the subunit *a*/*c* interface that are thought to conduct protons to and from the *c*-ring protonation sites (Allegretti et al., 2015). We find similar aqueous half-channels in equivalent positions of the *Y. lipolytica* dimer map (Figures 7A and 7B). Conserved hydrophilic residues of *a*H5 and *a*H6 line the aqueous cavity on the matrix side (Figures 7C and 7D). The conserved charged and polar residues of *a*H5, starting with Glu168 four helix turns upstream of Arg182, create the hydrophilic environment to release the proton from the opposing *c* subunit glutamate into the matrix. The ∼20° tilt of the *a*H5/*a*H6 hairpin places the hairpin loop close to the IMS surface, and the C terminus of *a*H6 on the matrix side (Figure 7A). Consequently, the lumenal half-channel near the hairpin loop and the matrix half-channel at the C terminus of subunit *a* are laterally offset, as anticipated (Junge et al., 1997, Vik and Antonio, 1994). The proton entrance channel on the IMS side is likely to include the conserved Asn186 in *a*H5, Asn106 in *a*H3, and the exchangeable pair His191/Glu229 in *a*H5 and *a*H6, one of which is Glu or Asp in all ATP synthases (Figure S5). Rather than mediating proton release, the essential Arg182 would allow only deprotonated *c* subunit glutamate side chains to pass (Figure 7D). Removal of this arginine by mutagenesis uncouples ion translocation from ATP synthesis (Mitome et al., 2010), as it results in futile proton translocation without *c-*ring rotation.

Clinical studies show that mutations in *a*H5 and *a*H6 impair the functionality or assembly of ATP synthase in human mitochondria (Kucharczyk et al., 2009, Xu et al., 2015), giving rise to severe neuropathological disorders (Houstek et al., 2006), such as the maternally inherited Leigh syndrome or retinitis pigmentosa (Kucharczyk et al., 2009). A molecular understanding of the exact ion translocation mechanism is essential for exploring future therapy. The structure of the genetically accessible *Y. lipolytica* ATP synthase now provides a basis for structural and functional studies to combat these diseases at the molecular level.

### Dimer Contacts

In the *Y. lipolytica* F_1_F_o_ dimer, which contains subunits *e* and *g*, the detergent belt is bent by roughly 100°. In the bovine F_1_F_o_ monomer, which contains subunits *e* and *g*, it is bent by ∼50° (Baker et al., 2012, Zhou et al., 2015), but in the *Y. lipolytica* monomer, which lacks *e* and *g*, the belt is more or less straight (Figure S7A; Table S1). We conclude that *e* and *g* are chiefly responsible for inducing the membrane curvature that results in mitochondrial cristae morphology.

There is no evident function for the C-terminal IMS helix of subunit *e* in the dimeric complex (Figure 6A). In contrast to the bovine map (Zhou et al., 2015), this elongated density protrudes straight out of the F_o_ stator region into the IMS, while in the bovine map it is curved and contacts the central plug of the *c*-ring. A role in the formation of higher-order ATP synthase oligomers and dimer rows (Strauss et al., 2008, Davies et al., 2012) seems unlikely, as the extension is not easily accessible and appears to point in the wrong direction for interaction between adjacent dimers (Figure 5D). Moreover, the dimer spacing along the rows is irregular (Daum et al., 2013), which suggests that the inter-dimer protein contacts are dynamic. Instead, the subunit *e* extension with its predicted coiled-coil propensity may play a role in complex assembly.

The two F_o_ subcomplexes in the membrane are separated by a wedge-shaped gap that is ∼40 Å wide on the matrix side and narrows to ∼15 Å on the IMS side (Figures 5G and 6A). On the matrix side, this wedge appears to be filled by lipid or detergent, as there is no distinct protein density. The tip of the wedge on the IMS side contains the dimer contact domain assigned to the C terminus of subunit *f* (Figure 6). The center of the wedge-shaped gap is bridged by a conspicuous sheet of density connecting the trans-membrane helices of subunits *a* and *8* (Figure 5G), which may contribute to dimer formation. It is tempting to speculate that this density belongs to the so far unassigned yeast-specific subunit *k*, a small, partly hydrophilic protein without predicted trans-membrane helix (Figure S4D). The sheet may form a barrier between the membrane leaflet on the matrix side and hydrophilic protein domains on the IMS side of the dimer interface. The matrix half of the wedge-shaped space has the thickness of one membrane leaflet, implying a new and unusual membrane architecture in this region of the dimer.

### Role of ATP Synthase Dimers in Membrane Morphology

The comparatively simple bacterial and chloroplast ATP synthases consist of eight or nine different subunits, which are sufficient for ATP production. The chloroplast ATP synthase has been shown to be monomeric (Daum et al., 2010), and no ATP synthase dimers have been reported in bacteria. By contrast, all known mitochondrial ATP synthases form dimers in the membrane that self-assemble into rows (Davies et al., 2012). Mitochondrial ATP synthases of yeasts and metazoans have eight additional subunits of so far unexplained structure and function. Our map shows how the mitochondria-specific subunits in the mitochondrial F_o_ subcomplex are arranged, and that most of them have a role in dimer formation: the N-terminal trans-membrane extension of *b* anchors *e* and *g* to the complex, and the *e*/*g* heterodimer induces local membrane curvature, which in turn appears to enable the IMS domain of *f* (and possibly subunit *k*) to establish protein-protein contacts across the dimer interface.

ATP synthase dimer rows are a prerequisite for the formation of inner membrane cristae (Davies et al., 2012), a hallmark signature of mitochondrial morphology. Cristae formation extends the membrane surface to accommodate a large number of respiratory chain complexes, making it possible to meet the high energy demands of eukaryotic cells (Lane and Martin, 2010). They also form a mitochondrial sub-compartment that supports a locally increased proton concentration in the confined cristae space. The structure of the mitochondrial ATP synthase dimer thus offers new insights into how mitochondria became the efficient power plants of eukaryotic cells.

## Experimental Procedures

ATP synthase dimers fully competent for oligomycin-sensitive ATP hydrolysis were isolated from mitochondria prepared from large-scale *Yarrowia lipolytica* cultures (Kashani-Poor et al., 2001) and purified by glycerol gradient centrifugation, anion exchange, and gel filtration chromatography. Cryo-EM grids of 2*YL*F_1_F_o_ dimers and 1*YL*F_1_F_o_ monomers were prepared and images were recorded on an in-column energy-filtered JEOL 3200 FSC electron microscope with a Gatan K2 direct electron detector in movie mode. Global beam-induced motion was corrected by movie frame processing (Li et al., 2013). Two- and three-dimensional classification and 3D map refinement were carried out with RELION 1.3 (Scheres, 2012). Crystals of the F_1_*c*_10_ subcomplex were grown from concentrated samples of the 1*YL*F_1_F_o_. X-ray data were collected at beamline PX-II X10SA (Swiss Light Source), and the structure was determined by molecular replacement with a model based on the *S. cerevisiae* F_1_*c*_10_ complex (PDB: 2XOK) (Stock et al., 1999).

## Author Contributions

T.M. initiated the study; T.M. and W.K. directed the project; V.Z. provided mitochondrial membranes; A.H. purified the protein; D.J.M. devised the cryo-EM data collection procedure; A.H. and D.J.M. collected cryo-EM data; A.H. and J.V. analyzed cryo-EM data; K.P. grew crystals and collected X-ray data; K.P., M.B., and T.M. analyzed X-ray data; T.M., J.V., W.K., K.P., M.B., and A.H. wrote the paper.

## Figures and Tables

**Table 1:** Table of Crystallography

	*Y. lipolytica* F_1_*c*_10_
**Data Collection**	

Wavelength (Å)	1.008
Space group	*P*2_1_2_1_2
Cell dimensions: *a*, *b*, *c* (Å)	169.5, 182.2, 193.0
Cell dimensions: α, β, γ (°)	90, 90, 90
Resolution (Å)	49.19–3.50 (3.60–3.50)^a^
Total reflections	1,477,286 (119,821)^a^
Unique reflections	75,882 (6,046)^a^
*R*_merged_	18.2 (>100)^a^
*I / σ(I)*	9.84 (0.61)^a^
Completeness (%)	99.99 (100.0)^a^
Redundancy	19.5 (19.8)^a^

**Refinement**	

Resolution (Å)	3.50
*R*_work_ / *R*_free_ (%)	27.39 / 30.54
Wilson β factor	158
Average β factor (Å^2^)	167
No. atoms	30,123
Protein	29,954
Ligands	152
Water	17^b^
RMSDs: bond lengths (Å)	0.006
RMSDs: bond angles (°)	0.883
PDB code	5FL7

a Values in parentheses are for highest-resolution shell.

b Water molecules coordinated to Mg^2+^ in the nucleotide binding sites (Figure S2E).

## Accession Numbers

The structure coordinates have been deposited in the Protein Data Bank under ID code PDB: 5FL7. The EM data were deposited in the worldwide PDB under ID codes wwPDB: EMD-8151 (symmetric dimer map); EMD-8152 (masked F_o_ map); and EMD-8153, EMD-8154, and EMD-8155 (subclass 1, 2, and 3 of the masked F_1_F_o_ monomer).

## Supplemental Information

Document S1. Supplemental Experimental Procedures, Figures S1–S8, and Tables S1–S3

Movie S1. *Y. lipolytica* ATP Synthase Dimer EM Map Related to Figure 2. Cartoon model of all assigned protein subunits is shown in one of the two monomers. Color code is as in Figure 2.

Movie S2. Morphed Conformational Changes in *Y. lipolytica* F_1_-ATP Synthase Related to Figure 1. Animation based on a morph between the three different conformational states observed in the *Y. lipolytica* F1*c*10 crystal structure. Subunit representations and coloring as follows: α, dark green or dark gray ribbon; β, light green or light gray ribbon; γ, blue surface; δ, cyan surface; ε, white surface. MgADP is represented as stick-ball model with C, N, O, P, and Mg colored in yellow, blue, red, orange, and magenta, respectively. The corner points for the morphs were generated by triplicating the entire molecule and then superposing the αDP subunit of the first copy and the αTP subunit of the second copy on the αE subunit of the original structure.

Movie S3. Morphed Conformational Changes in the α/β Heterodimer Related to Figure 1. Alternative view on the morphed animation shown also in Movie S2. For clarity, only one catalytic α/β heterodimer is shown, together with the rotating stalk containing subunits γ, δ, and ε. Representations and coloring as in Movie S2.

Movie S4. Comparison of Yeast and Bovine ATP Synthase Related to Figure 1. Superposed morphs of a catalytic α/β heterodimer from *B. taurus* (PDB: 1BMF; α, dark gray; β, light gray) (Abrahams et al., 1994) and *Y. lipolytica* (this work; α, dark green; β, light green). The α subunits were used as reference for both morphing and superposition. The conformations of the β subunits are most divergent in the βDP states, and more similar in the βTP and βE states. Also, the bovine structure undergoes larger overall conformational changes than the *Y. lipolytica* structure.

Movie S5. Comparison of *S. cerevisiae* and *Y. lipolytica* α/β Heterodimers Related to Figure 1. Superposed morphs of a catalytic α/β heterodimer from *S. cerevisiae* (PDB: 2HLD; α, dark gray; β, light gray) (Kabaleeswaran et al., 2006) and *Y. lipolytica* (this work; α, dark green; β, light green). The α subunits were used as reference for both morphing and superposition.

Document S2. Article plus Supplemental Information
