# Supplementary material for: Structure of a Complete ATP Synthase Dimer Reveals the Molecular Basis of Inner Mitochondrial Membrane Morphology
Source: Mol Cell. 2016 Aug 4;63(3):445–56. doi: 10.1016/j.molcel.2016.05.037 (PMC4980432; doi:10.1016/j.molcel.2016.05.037)
Supplement: Document S1. Supplemental Experimental Procedures, Figures S1–S8, and Tables S1–S3 [file mmc1.pdf]

Molecular Cell, Volume *63*

Supplemental Information

Structure of a Complete ATP Synthase Dimer Reveals the Molecular Basis of Inner Mitochondrial Membrane Morphology

Alexander Hahn, Kristian Parey, Maike Bublitz, Deryck J. Mills, Volker Zickermann, Janet Vonck, Werner Kühlbrandt, and Thomas Meier

SUPPLEMENTARY FIGURES

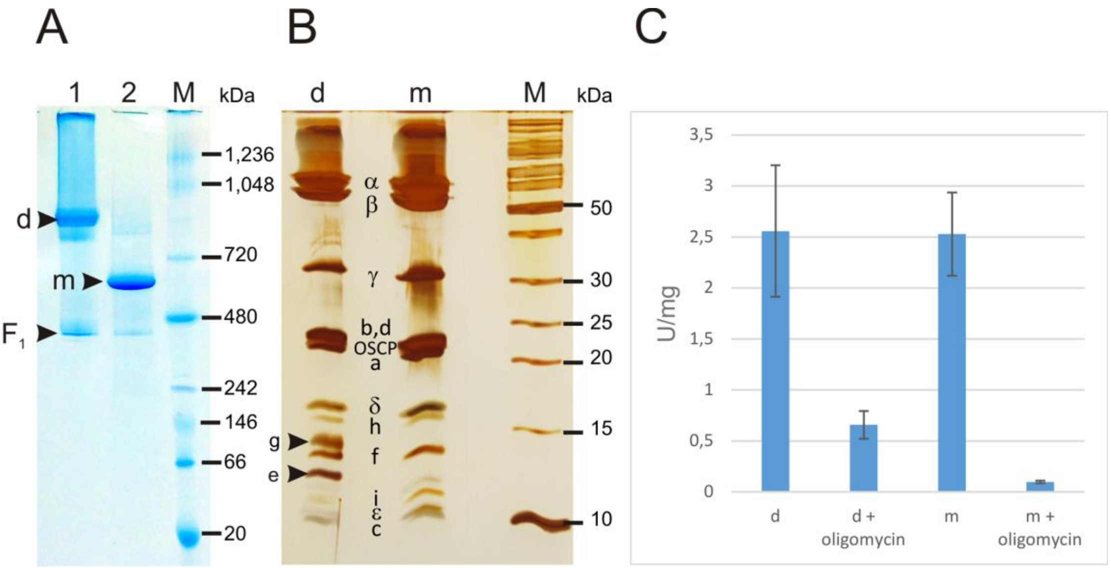

Figure S1, related to Figure 2. Subunit composition of monomeric and dimeric *Y. lipolytica* ATP synthase and ATP hydrolase activities of purified ATPases. The (1) dimeric (digitonin) and (2) monomeric (dodecylmaltoside) purified ATP synthase samples were separated by (**A**) Blue native-PAGE. Indicated protein complexes (d, m) were cut out of the gel and used for LC-MS analysis and 2nd dimension SDS-PAGE. (**B**) ATP synthase subunits were separated in the 2nd dimension SDS-PAGE using a 15% polyacrylamide gel, according to (Schägger and von Jagow, 1987). The subunits are indicated according to their molecular weight (Liu et al., 2015). Molecular weight markers (in kDa) are indicated on the right side of the gels. Arrow heads indicate the presence of subunit *e* and *g* only in the (d) dimer preparation but not in the (m) monomeric ATP synthase preparation. (**C**) Determination of ATP hydrolytic activities using the spectrophotometric coupled assay (Cook et al., 2003) and inhibition of ATP hydrolytic activity upon adding 5 µg/ml oligomycin (= 6.3 µM). Error bars are indicated (*n*=3).

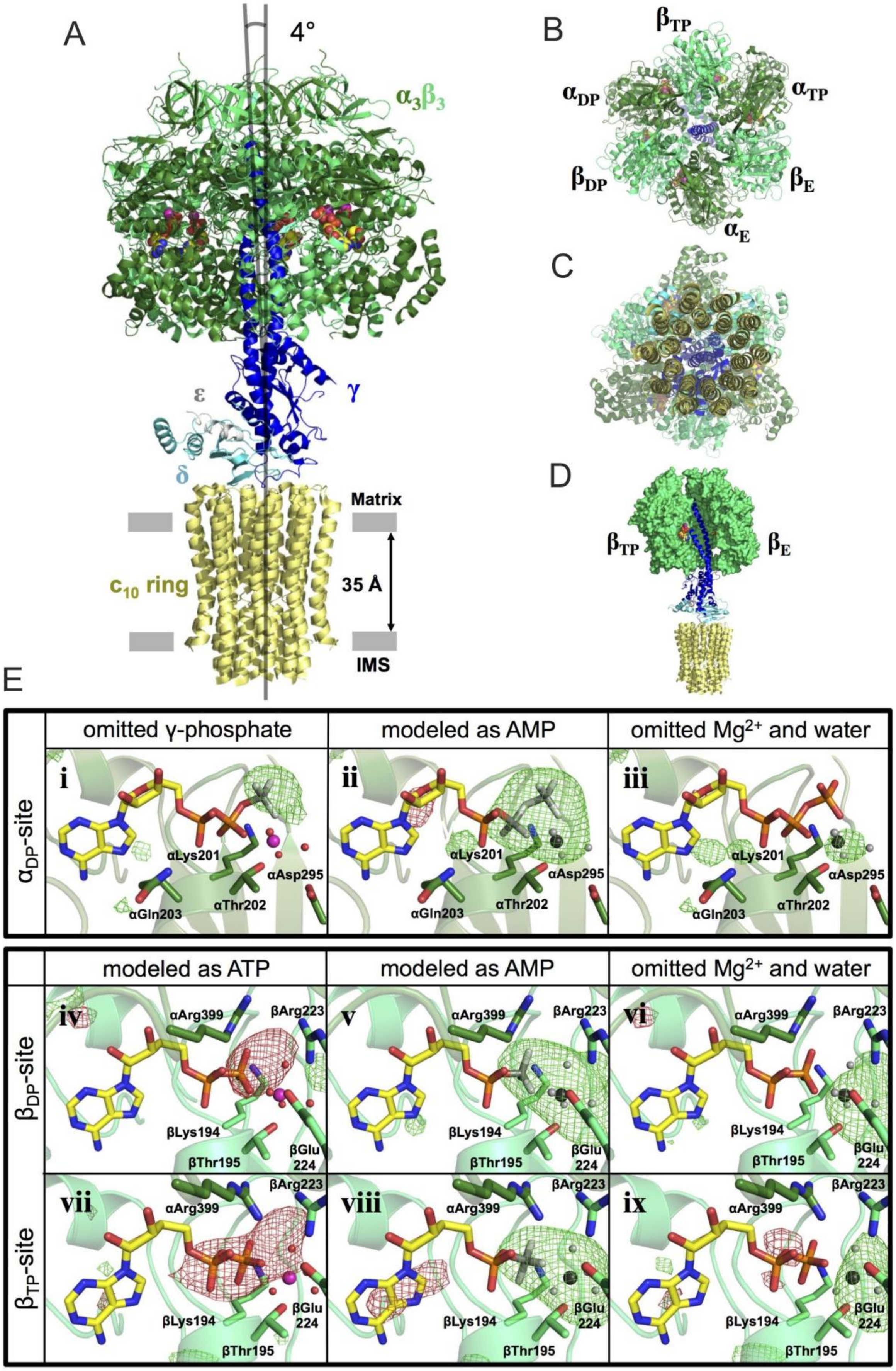
A
4°
α3β3
ε
γ
δ
Matrix
c10 ring
35 Å
IMS
B
βTP
αDP
αTP
βDP
βE
αE
C
D
βTP
βE
E
omitted γ-phosphate
modeled as AMP
omitted Mg2+ and water
αDP-site
i
ii
iii
αLys201
αAsp295
αThr202
αGln203
modeled as ATP
modeled as AMP
omitted Mg2+ and water
βDP-site
iv
v
vi
αArg399
βArg223
βLys194
βThr195
βGlu 224
βTP-site
vii
viii
ix

Figure S2, related to Figure 1. Crystal structure of the *Y. lipolytica* F_1c_{10} ATP synthase subcomplex. Overview from the (**A**) side, (**B**) top and (**C**) bottom. The structure is displayed in ribbon representation. The α- and β-subunits are shown in light and dark green, respectively, the γ-, δ- and ε-subunits are represented in blue, cyan and light grey, respectively, and the *c*-ring is colored in yellow. Nucleotides and Mg^{2+} are drawn as spheres. The symmetry axes of the F_1-stator and F_o-rotor, drawn as grey lines, are tilted by ~4° (as determined by Chimera). The membrane borders are indicated by grey bars. IMS: Intermembrane space. (**D**) Open F_1 (α-subunits omitted) in surface representation (β-subunits) and rotor in ribbon model. (**E**) Difference (omit) maps after modeling different nucleotide ligands at the α_{DP}-, β_{DP}- and β_{TP}-sites. The main chain is represented as a ribbon in dark and light green for the α- and β-subunits, respectively. Coordinating side chains are shown in stick representation. Nucleotides are also in stick representation with carbon, nitrogen, oxygen and phosphorus in yellow, blue, red and orange, respectively; Mg^{2+} and coordinating waters are depicted as spheres in magenta and red, respectively. Omitted atoms are indicated in grey, as a positional reference. The mF_{obs}-DF_{calc} electron density map after refinement is shown as mesh (contoured at ±3.0σ; green, positive; red, negative). *Upper panels*: α_{DP}-site after omitting (i) the γ-phosphate, (ii) the β- and γ-phosphate, Mg^{2+} and coordinating water, or (iii) Mg^{2+} and water. The positive peaks appearing after omitting parts of the ligand confirm the presence of Mg·ATP[·3 H_2O]. *Middle panels*: β_{DP}-site after (iv) refining with Mg·ATP[·3 H_2O] instead of Mg·ADP[·4 H_2O], (v) refining with AMP, or (vi) omitting Mg^{2+} and water. *Lower panels*: β_{TP}-site after (vii) refining with Mg·ATP[·3 H_2O] instead of Mg·ADP[·4 H_2O], (viii) refining with AMP, or (ix) omitting Mg^{2+} and water. In both the β_{DP}- and β_{TP}-site, negative density peaks appearing upon refinement of a triphosphate ligand indicate the absence of a γ-phosphate. In contrast, the presence of both the β-phosphates and the Mg^{2+} ions (including coordinating water) is supported by positive difference density peaks. While the resolution of the data does not allow to see density for water molecules directly, the presence of coordinated water at the Mg^{2+} ions was deduced from residual difference density peaks in the F_o-F_c maps if waters were omitted during the data analysis and from structure comparison with *S. cerevisiae* F_1 structures at higher resolution (Bowler et al., 2007; Kabaleeswaran et al., 2006).

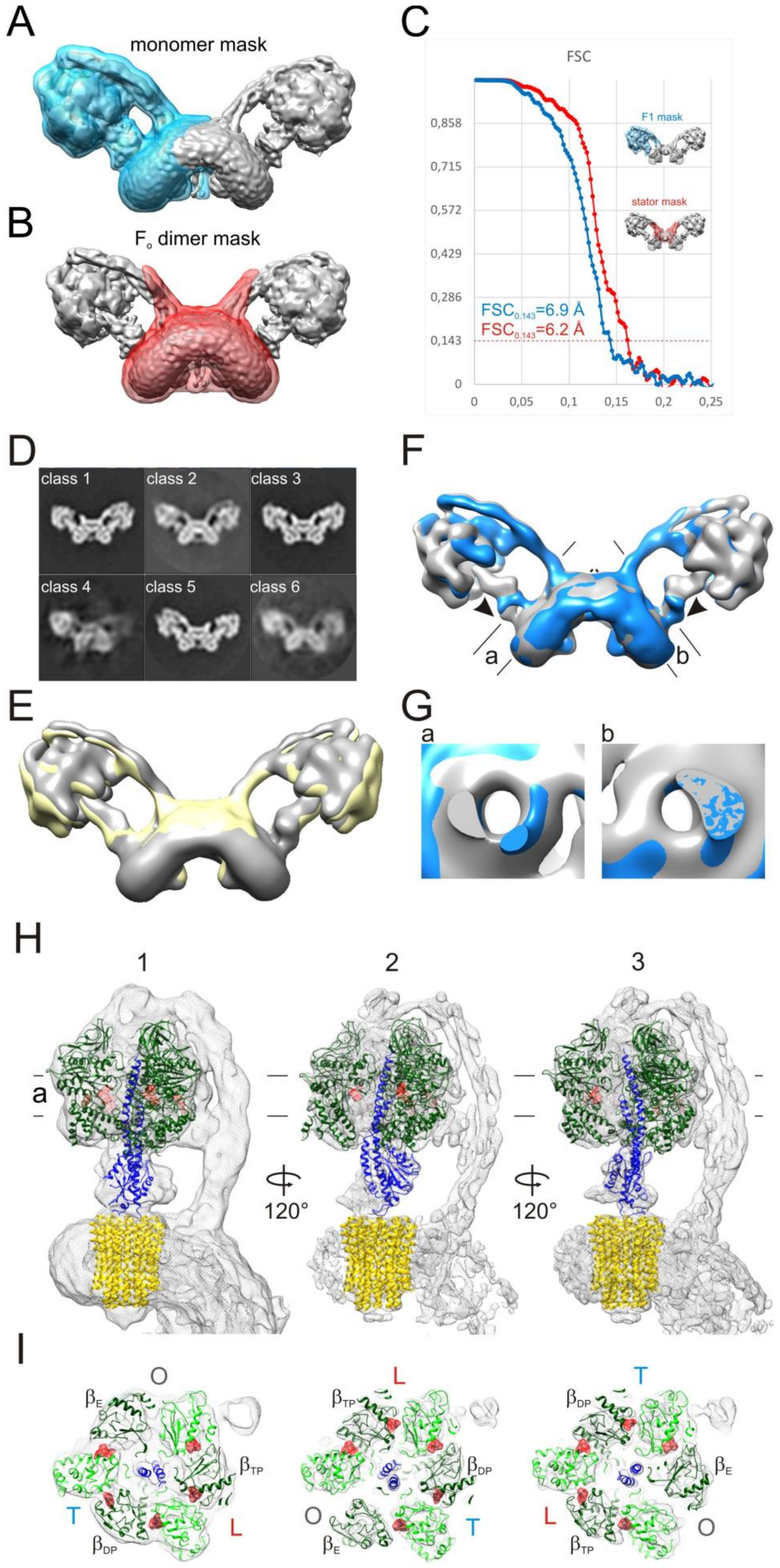
A
monomer mask
B
F_o dimer mask
C
FSC
F1 mask
stator mask
0,858
0,715
0,572
0,429
0,286
0,143
0
0
0,05
0,1
0,15
0,2
0,25
$FSC_{0,143}$=6.9 Å
$FSC_{0,143}$=6.2 Å
D
class 1
class 2
class 3
class 4
class 5
class 6
E
F
a
b
G
a
b
H
1
2
3
a
120°
120°
I
O
L
T
β_E
β_{TP}
β_{DP}
T
L
O
T
O
L
β_{TP}
β_{DP}
β_E
β_{DP}
β_E
β_{TP}

Figure S3, related to Experimental Procedures, Figure 1 and Figure 2. Conformational variability in the F_1F_o dimer. (**A-C**) Use of soft edge masks to compensate for conformational variability and estimation of local resolution of the *Y. lipolytica* ATP synthase dimer. The resolution of 3D reconstructions is limited by conformational variability and structural flexibility of the V-shaped dimers. To further improve the resolution of the reconstructed model, customized soft edge masks were used which only include selected subvolumes. (**A**) Mask that includes an F_1F_o monomer (blue) and (**B**) mask of the F_o-dimerization interface (red). Both masks were introduced during the last 3D refinement iterations in RELION-1.3 (Scheres, 2012). (**C**) Resolution of the masked F_1 head (blue) and masked F_o stator region (red) was determined by Fourier Shell Correlation (FSC). The resolution of F_1 and F_o stator was improved to 6.9 Å and 6.2 Å, respectively. (**D-G**) 3D classification of *Y. lipolytica* ATP synthase dimers reveals variation in the angle of the two monomers and asymmetric resting positions of two rotor elements within the dimer. (**D**) RELION-1.3 was used for 3D classification of 48,207 particles (selected after 2D classification). Six classes were generated. Class 1 (16,286 particles), class 3 (11,346 particles) and class 5 (11,047 particles) are very similar. Class 5 shows inverted handedness. Particles in class 2, 4 and 6 (9,628 particles total) do not form well shaped 3D volumes. All particles of class 2, 4 and 6 were discarded to clean the dataset before final 3D refinement of all particles. (**E**) Difference in the angle of the V-shaped dimer between class 1 (grey) and class 3 (yellow). (**F**) 38,679 particles were used for 3D reconstruction. The aligned dataset was used as starting point for 3D classification with an angular sampling interval of 1.8° and local angular search range of ±5° without applied symmetry to determine the structural heterogeneity of the dataset. Nine classes were generated to allow for structural flexibility during 3D classification. Class 4 (grey, 7,259 particles) and class 8 (blue, 6,862 particles) are superposed. (**G**) Slices through the maps in (F) (marked a and b) indicate variable rotor positions: In contrast to class 4 (grey) class 8 (blue) shows an asymmetric resting position of the two rotor elements in the dimer. The structural asymmetry of rotor positions within the dimer in the dataset suggests that the two F_1F_o monomers in an ATP synthase dimer are not functionally coupled. (**H, I**) Three different conformational states of the *Y. lipolytica* ATP synthase monomer complexes. The states are separated by 3D classification with an angular sampling interval of 1.8° and local angular search range of ±5° combined with an F_1F_o monomer soft edge mask (A-C). 38,679 particles were used for 3D classification. The majority of particles were sorted into subclass 2 and 3. The three classes are *subclass 1*: 5,621 (15.3%), *subclass 2*: 16,671 (45.5%) and *subclass 3*: 14,379 (39.2%) particles. Particles in each subclass were used for a final 3D refinement in RELION-1.3 (Scheres, 2012). (**H**) The three final maps are distinguished by 120° rotations of the central stalk subunit γ (shown in blue). (**I**) Cross sections of the three states at the level of the nucleotide binding sites. The α/β headpiece (light green/dark green) shows different conformations corresponding to the 'Boyer-Walker' states described as *open* (β_E), *loose* (β_{TP}) and *tight* (β_{DP}) (Abrahams et al., 1994; Boyer, 1997). The view from the matrix side shows the counterclockwise transition of the α/β subunits in synthesis direction. The nucleotides are indicated in red, according to the *Y. lipolytica* F_1c_{10} structure (this work). The *c*-ring is indicated in yellow.

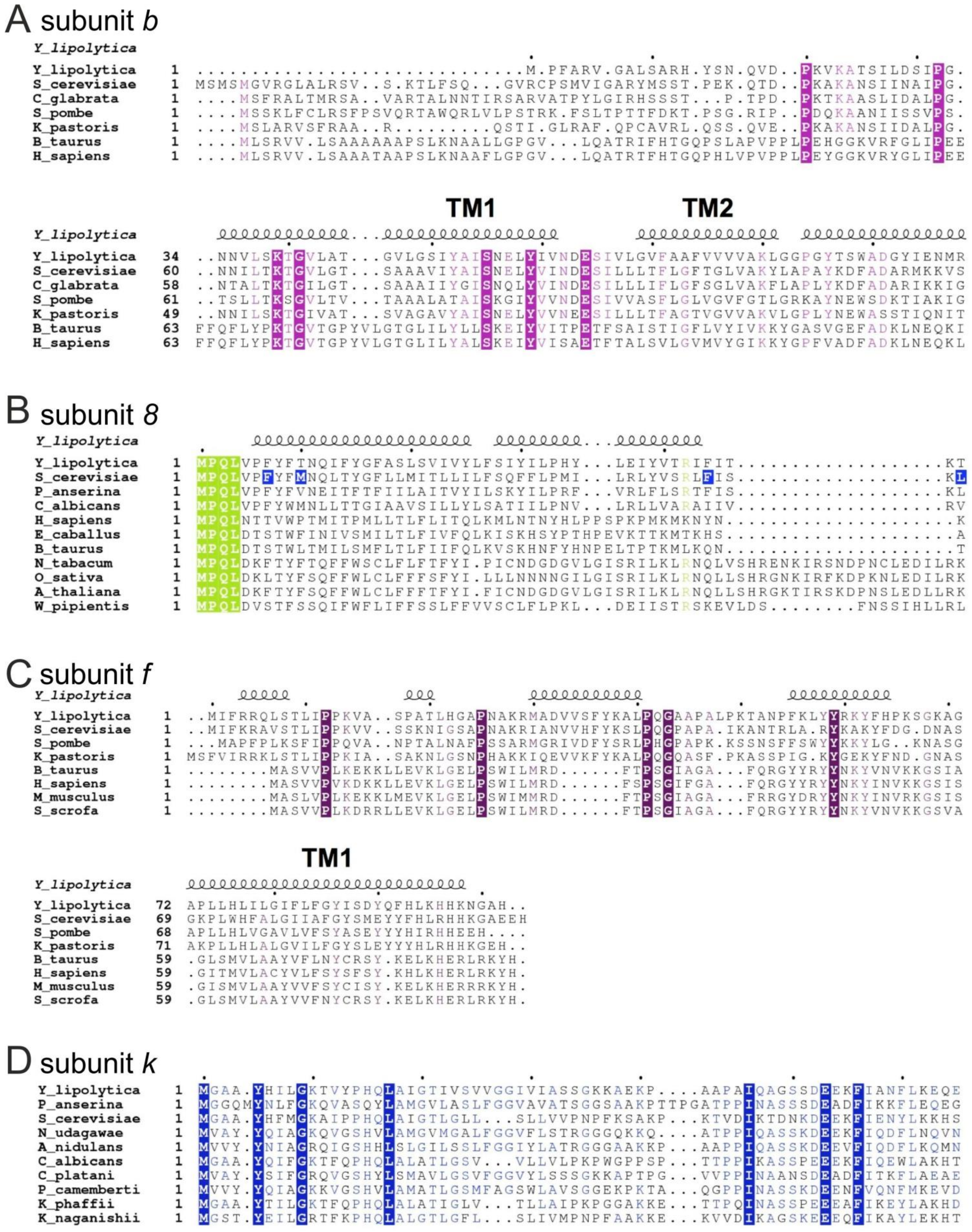
A subunit b
TM1
TM2
B subunit 8
C subunit f
TM1
D subunit k

Figure S4, related to Figure 3, Figure 5 and Figure 6. Sequence alignments of subunits *b*, *8*, *f* and *k*. (**A**) Subunit *b*. The alignment was created using the sequences from *Yarrowia lipolytica, Saccharomyces cerevisiae, Candida glabrata*, *Schizosaccharomyces pombe*, *Komagataella pastoris*, *Bos taurus* and *Homo sapiens*. (**B**) Subunit *8*. The alignment was created by using the sequences from *Yarrowia lipolytica*, *Saccharomyces cerevisiae, Podospora anserina*, *Candida albicans*, *Homo sapiens*, *Equus caballus* and *Bos taurus* with subunit *b* from *Nicotiana tabacum*, *Oryza sativa*, *Arabidopsis thaliana* and *Wolbachia pipientis*. Crosslinking residues (**Figure S6**) are represented in blue boxes. (**C**) Subunit *f*. The alignment was created using the sequences from *Yarrowia lipolytica*, *Saccharomyces cerevisiae*, *Schizosaccharomyces pombe*, *Komagataella pastoris*, *Bos taurus*, *Homo sapiens*, *Mus musculus* and *Sus scrofa*. (**D**) Subunit *k*. The alignment was created using the sequences from *Yarrowia lipolytica*, *Podospora anserina*, *Saccharomyces cerevisiae*, *Aspergillus nidulans* FGSC A4, *Neosartorya udagawae*, *Candida albicans* P94015, *Ceratocystis platani*, *Penicillium camemberti*, *Komagataella phaffii* GS115 and *Kazachstania naganishii* CBS 8797. The secondary structure is indicated according to the model based on the cryoEM structure. The alignments do not show the complete sequences (except subunit *f*).

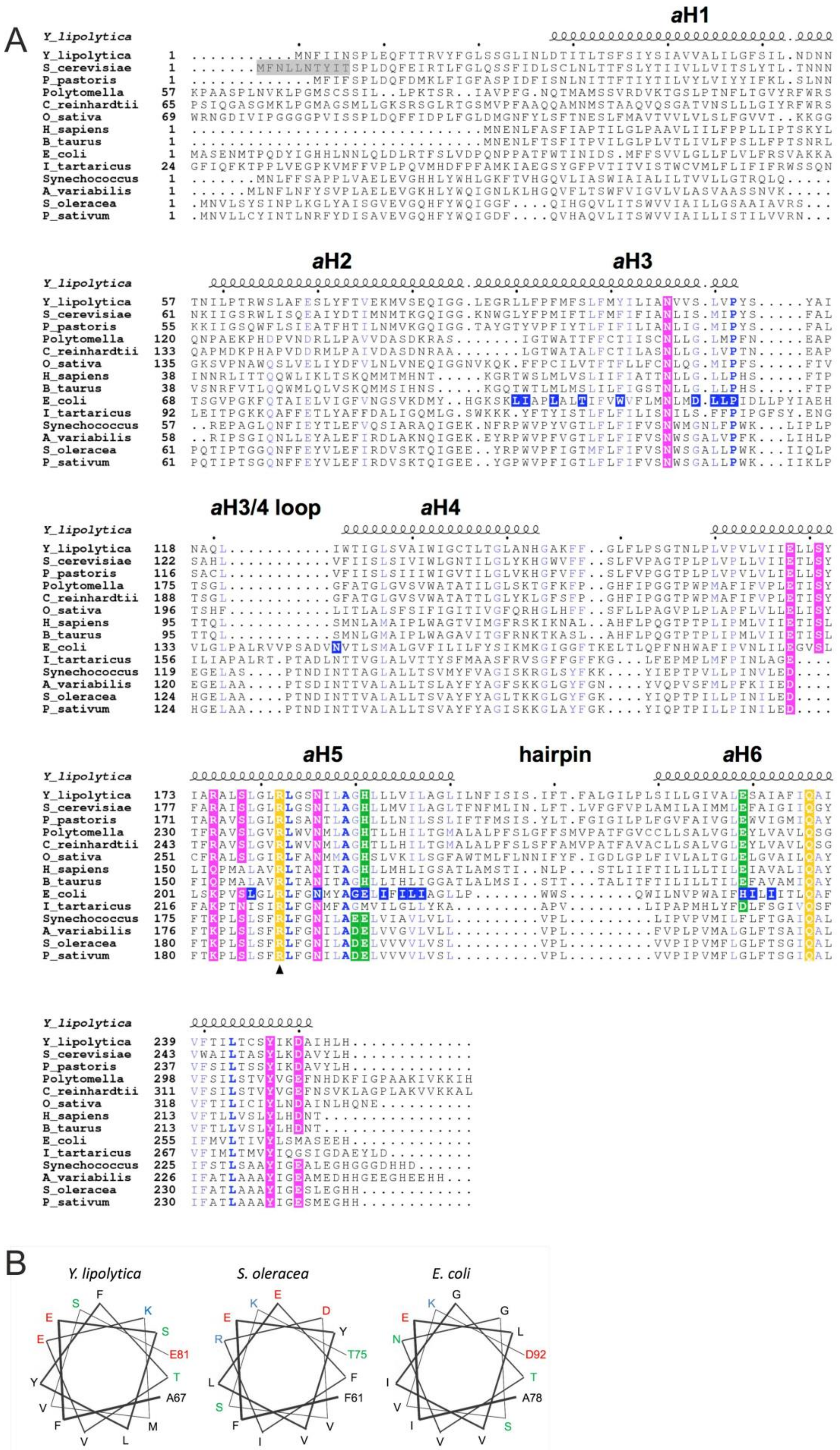
A
aH1
aH2
aH3
aH3/4 loop
aH4
aH5
hairpin
aH6
Y_lipolytica
S_cerevisiae
P_pastoris
Polytomella
C_reinhardtii
O_sativa
H_sapiens
B_taurus
E_coli
I_tartaricus
Synechococcus
A_variabilis
S_oleracea
P_sativum
B
Y. lipolytica
S. oleracea
E. coli
E81
A67
T75
F61
D92
A78

Figure S5, related to Figure 4. Sequence alignment and helical wheels of subunit *a*. (**A**) The alignment was generated from sequences of *Yarrowia lipolytica, Saccharomyces cerevisiae, Pichia pastoris, Polytomella* sp. Pringsheim, *Chlamydomonas reinhardtii*, *Oryza sativa*, *Homo sapiens*, *Bos taurus*, *Escherichia coli*, *Ilyobacter tartaricus, Synechococcus* sp., *Anabaena variabilis*, *Spinacia oleracea* and *Pisum sativum*. The secondary structure assignment is based on the model of the cryoEM structure. The interchangeable residue pairs *a*Arg175/*a*Gln236 and *a*Glu229/*a*His191 are yellow and green, respectively (Cain, 2000; Eya et al., 1991; Lightowlers et al., 1987). Conserved charged and polar residues are highlighted in pink. The black arrowhead marks the strictly conserved arginine essential for coupled proton translocation. Crosslinking residues are represented in blue boxes. The signal sequence of *S. cerevisiae* is represented as a bar in light grey. (**B**) Helical wheels for the predicted helix *a*H2 show evidence for a conserved amphipathic helix in all species in mitochondria (exemplified by *Y. lipolytica*), chloroplasts (*S. oleracea*) and bacteria (*E. coli*). Hydrophobic residues are shown in black, positively charged in blue, negatively charged in red, polar in green.

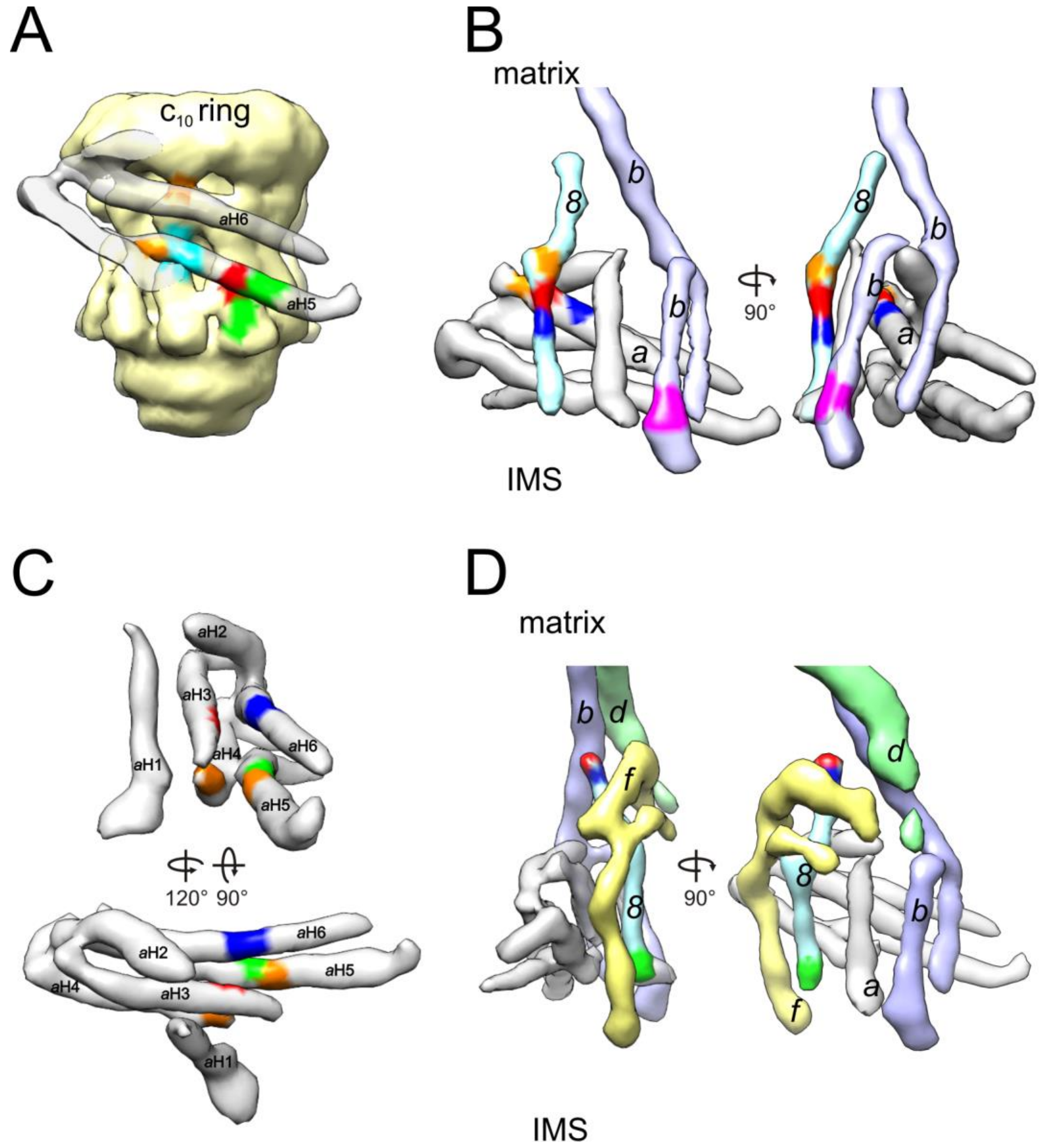

Figure S6, related to Figure 6. Cys-Cys crosslink positions in *E. coli* and *S. cerevisiae*. To verify the assignment of α-helices in the F_o region of the *Y. lipolytica* F_1F_o dimer, we compared available crosslink studies for *E. coli* and *S. cerevisiae* (DeLeon-Rangel et al., 2013; Jiang and Fillingame, 1998; Schwem and Fillingame, 2006; Stephens et al., 2003) with our ATP synthase dimer model. By using multiple pairwise sequence alignment ESPript (Robert and Gouet, 2014) we generated a homology model and assigned corresponding amino acids in **Table S3**. To visualize the crosslink positions, the crosslinks are shown as color pairs on the segmented cryo-EM volumes as indicated in **Table S3**. (**A**) Cys-Cys crosslink positions of subunit *a*H5 to the c_{10} ring. The crosslink pairs follow the curvature of *a*H5 around the c_{10} ring. (**B**) Subunit *8* is in close proximity to *a*H2 and *a*H3. Val111 and Pro112 of subunit *a* are located in the flexible loop region between *a*H3 and *a*H4, which lies in close proximity to Ser55 from subunit *b* (pink). (**C**) Crosslink positions in the horizontal double hairpin between *a*H3 and *a*H5 (red/green), *a*H4 and *a*H5 (orange), *a*H3 and *a*H6 (red/blue) and *a*H5 and *a*H6 (green/blue). (**D**) Crosslinks between subunit *8* and subunit *b*, *d* and *f* (red/blue) on the matrix side. Our model places the N terminus of subunit *8* (green) and the C terminus of *f* in close proximity at the IMS, which also matches with *S. cerevisiae* crosslinks (Stephens et al., 2003).

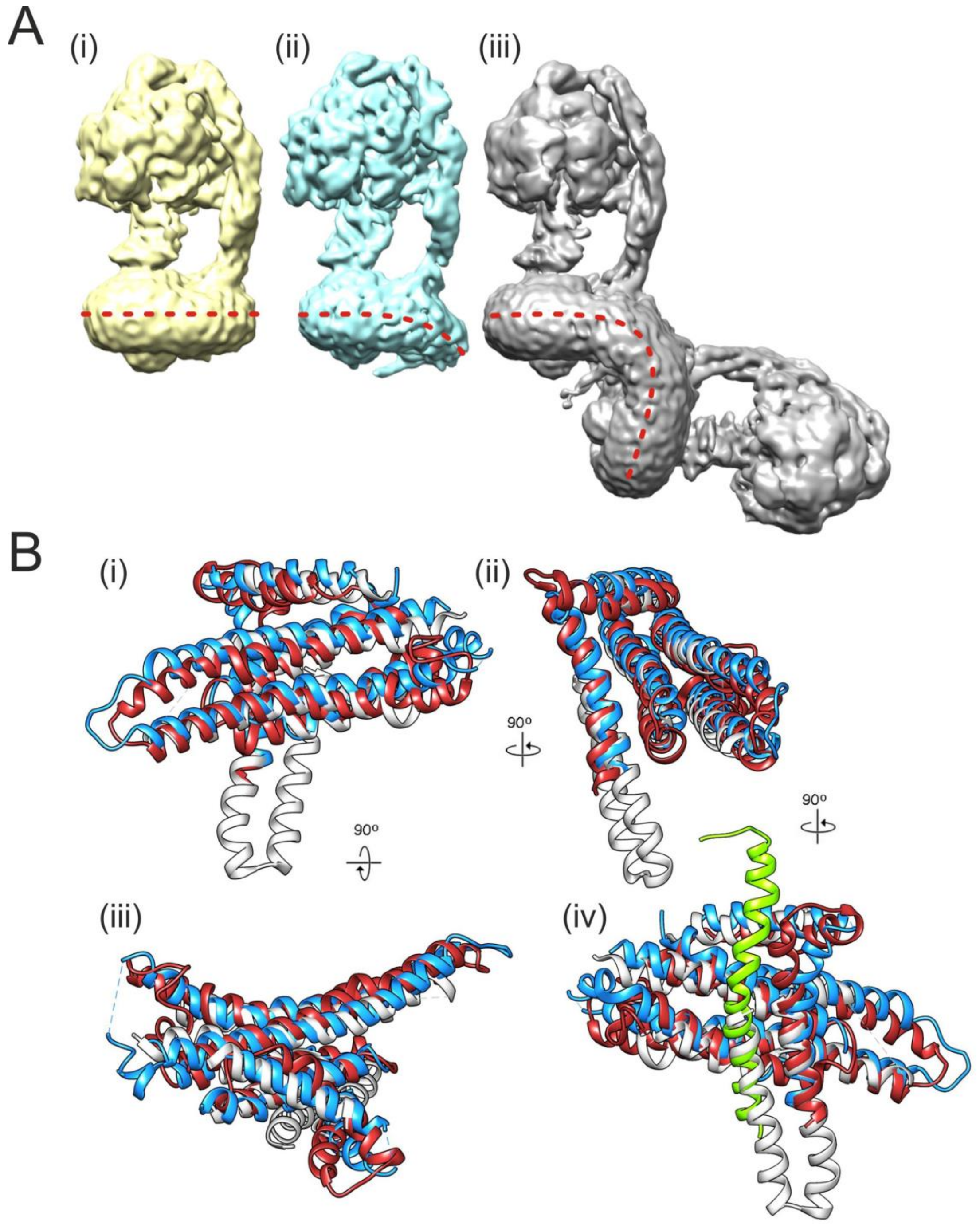

Figure S7, related to Figure 4 and Figure 5. (**A**) Subunit *e* and *g* bend the inner mitochondrial membrane. (i) Monomeric ATP synthase of *Y. lipolytica* (8.4 Å). Subunit *e* and *g* and the fungi-specific subunit *k* are lost during purification with the detergent dodecyl maltoside (**Table S1**). The detergent belt forms a level, unbent ring around the F_o sector. (ii) Monomeric bovine ATP synthase (Zhou et al., 2015): EMD-3165, 7.4 Å. The dimer-specific subunits *e* and *g* were retained during purification. The detergent belt bends away from the *c*-ring, probably due to the presence of subunits *e* and *g*. (iii) Dimeric ATP synthase of *Y. lipolytica* at 7.8 Å resolution. Subunits *e*, *g* and *k* were retained during purification in the mild detergent digitonin (**Table S1**). The F_o sector is strongly bent, presumably by subunits *e* and *g*. This might force both halves of the dimer into a position where subunit *f* can form the dimer contact. (**B**) Comparison of subunit *a* from different species. *Y. lipolytica* mitochondria, blue (this work); *Paracoccus denitrificans* (5DN6), white (Morales-Rios et al., 2015); bovine heart

mitochondria (5ARA), brown (Zhou et al., 2015). (i) View from the *c*-ring towards the *a*H5-*a*H6 hairpin. The matrix side is on top. (ii) Side view. (iii) Top view from the matrix side. (iv) View from the dimer interface. Subunit *8* of *Y. lipolytica* is shown in green. This subunit superposes well on an unassigned trans-membrane helix in the *P. denitrificans* ATP synthase. We identify this unassigned helix as belonging to one of the two bacterial *b*-subunits. There is no density for the second *b*-subunit trans-membrane helix in the *P. denitrificans* X-ray map.

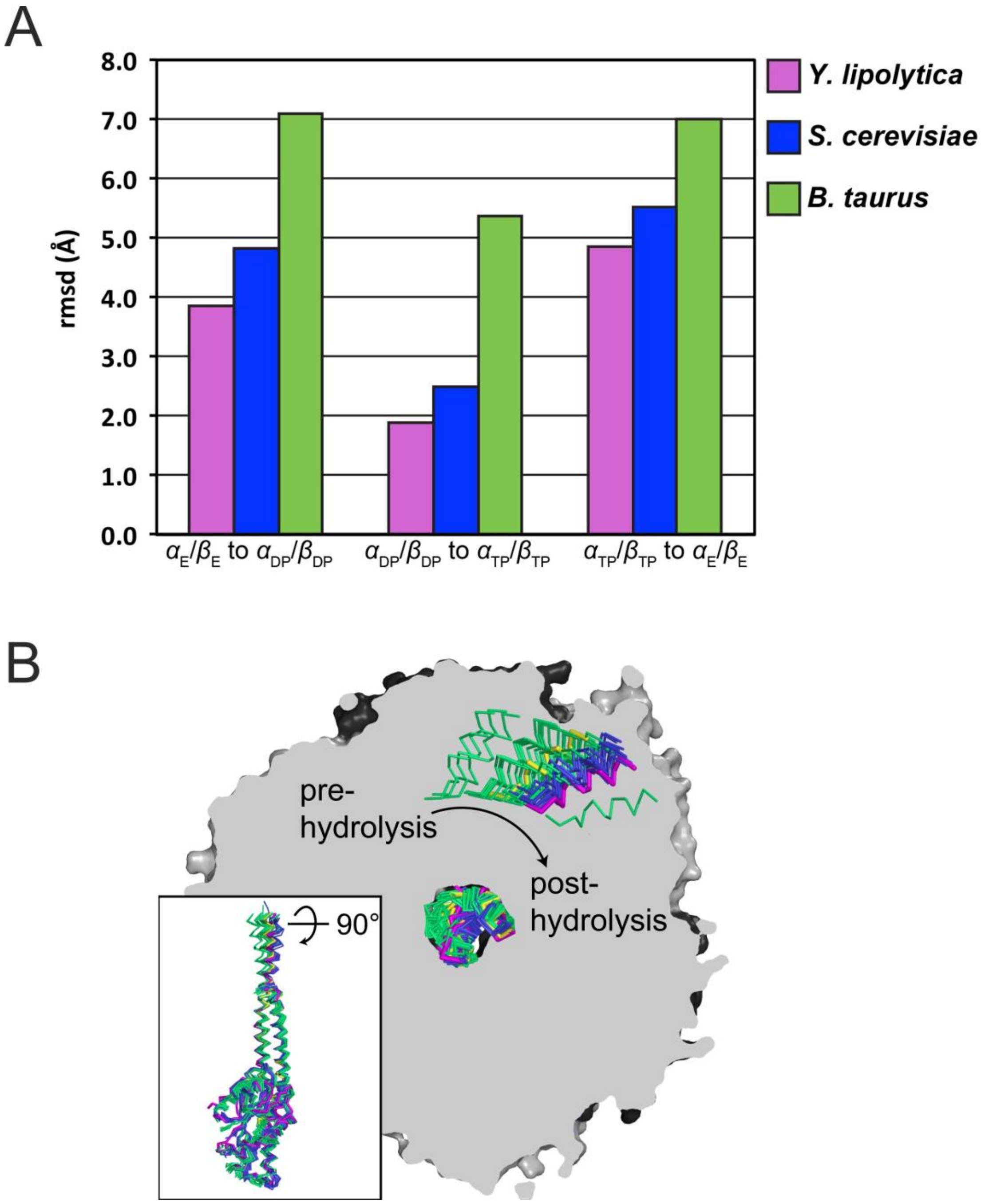

Figure S8, related to Figure 1. Comparison of the *Y. lipolytica* F_1c_{10} structure with *B. taurus* and *S. cerevisiae* F_1 and F_1c_{10} structures. (**A**) Structural differences between α/β heterodimers in *Y. lipolytica*, *S. cerevisiae* and *B. taurus* ATP synthase. To compare the structural differences within the α/β heterodimer, including the catalytic site in the β-subunits, the root mean square deviation (rmsd) values were calculated using the α/β heterodimers of the F_1 sectors from *Y. lipolytica* F_1c_{10} structure (*YL*F_1c_{10}, this work), *S. cerevisiae* structure PDB: 2HLD (Kabaleeswaran et al., 2006) and the F_1 reference structure, PDB: 1BMF (Abrahams et al., 1994). The rmsd values of the three different conformational Boyer states *open*, *tight* and *loose* (Boyer, 1997), corresponding to β_E, β_{DP} and β_{TP} (Abrahams et al., 1994), respectively, are largest within the structure of the bovine F_1 (PDB: 1BMF), while the inhibitor-free *YL*F_1c_{10}

exhibits the smallest overall conformational differences. (B) Rotational positioning of the *Yarrowia lipolytica* F_1c_{10} ATP synthase γ-subunit and P-loop in comparison with other F_1 crystal structures from *Saccharomyces cerevisiae* and *Bos taurus*. The angular position of the γ-subunit relative to the α_3/β_3 head piece was visualized by aligning the structures on the γ-subunits (the overall alignment is shown in the inset in the bottom left corner; see Methods for details) and comparing the angular position of the P-loops in the β_{DP} site of the F_1 sector (depicted as colored ribbons). The following structures were compared from *B. taurus* (green): PDBs: 1BMF, 1COW, 1E1Q, 1E1R, 1E79, 1EFR, 1H8E, 1H8H, 1NBM, 1W0J, 1W0K, 2CK3, 2JDI, 2JIZ, 2JJ1, 2JJ2, 2V7Q, 2WSS, 2XND, 4ASU, 4TSF, 4TT3, 4YXW, and 4Z1M (Abrahams et al., 1996; Abrahams et al., 1994; Bason et al., 2014, 2015; Bowler et al., 2006, 2007; Braig et al., 2000; Gledhill et al., 2007; Kagawa et al., 2004; Menz et al., 2001a; Menz et al., 2001b; Orriss et al., 1998; Rees et al., 2009; Rees et al., 2012; van Raaij et al., 1996; Watt et al., 2010). The reference structure (Abrahams et al., 1994) is highlighted in yellow. *S. cerevisiae* (blue), PDBs: 2HLD, 2WPD, 2XOK, 3FKS, 3OE7, 3OEE, 3OEH, 3OFN, 3ZIA, 3ZRY (Arsenieva et al., 2010; Dautant et al., 2010; Giraud et al., 2012; Kabaleeswaran et al., 2006; Kabaleeswaran et al., 2009; Robinson et al., 2013; Stock et al., 1999). Purple: *Y. lipolytica*. The view is from the membrane, the *Y. lipolytica* F_1 sector is depicted as a cross-section at the approximate level of the P-loop. The ATP hydrolysis rotation direction of α_3/β_3 relative to the central stalk is indicated by an arrow. The *Y. lipolytica* P-loop is shifted the furthest to a post-hydrolysis state, as compared to the majority of the *S. cerevisiae* and *B. taurus* F_1 structures, with only one exception, the *B. taurus* post-hydrolysis (pre-product release) structure 4ASU (Rees et al., 2012).

Supplementary Movies

Movie S1, related to Figure 2. *Y. lipolytica* ATP synthase dimer EM map. Cartoon model of all assigned protein subunits is shown in one of the two monomers. Color code is as in **Figure 2**.

Movie S2, related to Figure 1. Morphed conformational changes in *Y. lipolytica* F_1-ATP synthase. Animation based on a morph between the three different conformational states observed in the *Y. lipolytica* F_1c_{10} crystal structure. Subunit representations and coloring as follows: *α*, dark green or dark grey ribbon; *β*, light green or light grey ribbon; *γ*, blue surface; *δ*, cyan surface; *ε*, white surface. MgADP is represented as stick-ball model with C, N, O, P, and Mg colored in yellow, blue, red, orange, and magenta, respectively. The corner points for the morphs were generated by triplicating the entire molecule and then superposing the α_{DP} subunit of the first copy and the α_{TP} subunit of the second copy on the α_E subunit of the original structure.

Movie S3, related to Figure 1. Morphed conformational changes in the α/β heterodimer. Alternative view on the morphed animation shown also in **Movie S2**. For clarity, only one catalytic *α*/*β* heterodimer is shown, together with the rotating stalk containing subunits *γ*, *δ*, and *ε*. Representations and coloring as in **Movie S2**.

Movie S4, related to Figure 1. Comparison of yeast and bovine ATP synthase. Superposed morphs of a catalytic *α*/*β* heterodimer from *B. taurus* (PDB: 1BMF; *α*, dark gray; *β*, light gray) (Abrahams et al., 1994) and *Y. lipolytica* (this work, *α*, dark green; *β*, light green). The *α*-subunits were used as reference for both morphing and superposition. The conformations of the *β*-subunits are most divergent in the β_{DP} states, and more similar in the β_{TP} and β_E states. Also, the bovine structure undergoes larger overall conformational changes than the *Y. lipolytica* structure.

Movie S5, related to Figure 1. Comparison of *S. cerevisiae* and *Y. lipolytica* α/β heterodimers. Superposed morphs of a catalytic *α*/*β* heterodimer from *S. cerevisiae* (PDB: 2HLD; *α*, dark gray; *β*, light gray) (Kabaleeswaran et al., 2006) and *Y. lipolytica* (this work, *α*, dark green; *β*, light green). The *α*-subunits were used as reference for both morphing and superposition.

Supplementary Tables

Table S1, related to Figure 2 and Figure 5. Protein subunits in *Y. lipolytica* monomeric and dimeric ATP synthase, analyzed by LC-MS[*].

					**Dimer**		**Monomer**	
	**subunit**	**dimer**	**monomer**	**size (kDa)**	**sequence coverage (%)**	**score**	**sequence coverage (%)**	**score**
**F_1**	***α***	+ [a,b]	+ [a,b]	58	50.9[a], 62.7[b]	1993.48[a], 1918.78[b]	58[a], 52.4[b]	2158.7[a], 1415.97[b]
	β	+ [a,b]	+ [a,b]	54.5	55.2[a], 57.2[b]	1716.49[a], 1160.33[b]	61.7[a], 38.7[b]	1727.06[a], 726.89[b]
	γ	+ [a,b]	+ [a,b]	32.3	37.5[a], 41.30[b]	664.48[a], 390.50[b]	46.4[a], 43.7[b]	838.31[a], 473.33[b]
	δ	n.d.	n.d.	-	-	-	-	-
	ε	n.d.	n.d.	-	-	-	-	-
	OSCP	+ [a,b]	+ [a,b]	22.8	25.7[a], 32.2[b]	219.4[a], 299.74[b]	48.6[a], 32.7[b]	512.61[a], 307.53[b]
**F_o**	***a***	+ [a,b]	+ [a,b]	27.8	7.5[a], 7.1[b]	141.9[a]5, 88.2[b]	7.1[a], 3.1[b]	116.46[a], 80.84[b]
	b	+ [a,b]	+ [a,b]	24	46.1[a], 34.6[b]	556.2[a], 264.85[b]	48.8[a], 12.9[b]	589.47[a], 133.45[b]
	c	n.d.	n.d.		-	-		
	d (F6)	+ [a,b]	+ [a,b]	19.8	46.6[a], 46.6[b]	451.47[a], 289.53[b]	47.2[a], 43.8[b]	439.34[a], 285.17[b]
	f	+ [a,b]	+ [a,b]	11.4	42.7[a], 47.6[b]	473.38[a], 322.14[b]	41.7[a], 47.6[b]	347.87[a], 337.87[b]
	h	n.d.	n.d.	-	-	-	-	-
	i	+ [b]	+ [b]	6.9	59.7[b]	132.48[b]	43.5[b]	136.8[b]
	8 (A6L)	n.d.	n.d.	-	-	-	-	-
	e	+ [a,b]	n.d.	10	48.9[a], 28.9[b]	158.46[a], 142.68[b]	-	-
	g	+ [a,b]	n.d.	15.1	22.5[a], 49.3[b]	156.25[a], 284.93[b]	-	-
	k	+ [a]	n.d..	7.3	37.1[a]	150.8[a]	-	-

[a]Trypsin, [b]Chymotrypsin, n.d., not detected, ( ) alternative nomenclature for bovine,
[*]for details see Supplementary Experimental Procedures

Table S2, related to Figure 1. Comparison of the *Y. lipolytica* α/β heterodimers with other crystal structures. Structures of the three α/β heterodimers in the F_1 sectors of ATP synthase from *Y. lipolytica* (this work), *B. taurus* PDB: 1BMF (Abrahams et al., 1994) and *S. cerevisiae*, PDB: 2HLD (Kabaleeswaran et al., 2006) were compared. The root mean square deviation (rmsd)-values (Å) between the main chain atoms of the respective heterodimers were determined with the 'align' function in PyMOL (Schrödinger, LLC).

		Y. lipolytica			***B. taurus***			***S. cerevisiae***	
		α_E/β_E	α_{DP}/β_{DP}	α_{TP}/β_{TP}	α_E/β_E	α_{DP}/β_{DP}	α_{TP}/β_{TP}	α_E/β_E	α_{TP}/β_{TP}
Y. lipolytica	α_E/β_E								
	α_{DP}/β_{DP}	3.85							
	α_{TP}/β_{TP}	4.84	1.89						
B. taurus	α_E/β_E	1.89	4.28	5.35					
	α_{DP}/β_{DP}	5.54	2.63	2.70	7.11				
	α_{TP}/β_{TP}	5.32	2.88	2.07	7.00	5.38			
S. cerevisiae	α_E/β_E	1.28	4.27	5.31	2.95	6.04	6.05		
	α_{DP}/β_{DP}	4.11	1.97	2.46	4.90	3.11	3.23	4.84	
	α_{TP}/β_{TP}	4.96	2.44	1.75	5.71	3.43	2.67	5.54	2.49

Table S3, related to Figure 6. Cys-Cys crosslink positions in *E. coli, S. cerevisiae* and *Y. lipolytica*. List of residue pairs that form Cys-substitution crosslinks in *E. coli* and *S. cerevisiae*. Homologous residues in *Y. lipolytica* were identified by sequence alignments (**Figures S4** and **Figure S5**). For each crosslink study (A-D) a color code was assigned to show the position of each homologous crosslink pair in the segmented EM subvolume of *Y. lipolytica* (**Figure S6**).

	E. coli[1]	*Y. lipolytica*
A	*a* L207/*c* I55	*a* L179/c_{10} L53
	a N214/*c* A62	*a* N186/c_{10} A60
	a N214/*c* M65	*a* N186/c_{10} L63
	a I221/*c* G69	*a* L193/c_{10} M67
	a I223/*c* L72	*a* V195/c_{10} F70
	a L224/*c* Y73	*a* I196/c_{10} L71
	a I225/*c* Y73	*a* L197/c_{10} L71
	E. coli[2]	*Y. lipolytica*
B	*a* P122/*b* N4	*a* P112/*b* S55
	a L121/*b* N4	*a* V111/*b* S55
	a W111/*b* F14	*a* Y101/*8* L20
	a W111/*b* V15	*a* Y101/*8* S21
	a T107/*b* V18	*a* S97/*8* V24
	a L104/*b* M22	*a* F94/*8* S28
	a I101C/*b* W26	*a* P91/*8* L32
	a L100C/*b* W26	*a* L90/*8* L32
	E. coli[3]	*Y. lipolytica*
C	*a* D119/*a* G218	*a* S109/*a* G190
	a L120/*a* G218	*a* L110/*a* G190
	a N148/*a* E219	*a* I122/*a* H191
	a L120/*a* I246	*a* L110/*a* S230
	a L120/*a* H245	*a* L110/*a* E229
	a G218/*a* I248	*a* G190/*a* I232
	S. cerevisiae[4]	*Y. lipolytica*
D	*8* F44/sub. *b* matrix	*8* F44/sub. *b* matrix
	8 F44/sub. *d* matrix	*8* F44/sub. *d* matrix
	8 F44/sub. *f* matrix	*8* F44/sub. *f* matrix
	8 L48/sub. *d* matrix	*8* T48/sub. *d* matrix
	8 L48/sub. *f* matrix	*8* T48/sub. *f* matrix
	8 M10/*a* C23	*8* T10/*a* 23
	8 F7/*a* C23	*8* F7/*a* 23
	8 F7/sub. *f* IMS	*8* F7/sub. *f* IMS

[1](Jiang and Fillingame, 1998)
[2](DeLeon-Rangel et al., 2013)
[3](Schwem and Fillingame, 2006)
[4](Stephens et al., 2003)

SUPPLEMENTARY EXPERIMENTAL PROCEDURES

Isolation of *Yarrowia lipolytica* ATP synthase dimers and monomers. Large-scale fermentation of *Y. lipolytica* and isolation of mitochondrial membranes were carried out as previously described (Hunte et al., 2010). Complex I was removed by metal affinity purification. For long-term storage at -80°C, glycerol was added to a final concentration of 20% (v/v) before solubilized membranes were rapidly frozen in liquid nitrogen. For purification of intact ATP synthase dimers (2*YL*F_1F_o), the sample was diluted 1:1 with Buffer A [30mM MOPS-NaOH pH 7.5, 2 mM $MgCl_2$, 0.5 mM EDTA, 50 mM NaCl, 0.2% (w/v) digitonin (AppliChem, D)] to a total volume of 16 ml. Subsequently 50% (w/w) PEG 6000 was added to a final concentration of 3%. After 15 min incubation on ice the protein precipitate was removed by centrifugation for 15 min at 20,000 x *g*. The supernatant was loaded on a discontinuous glycerol gradient (55-25% in 5% steps) of Buffer A and run at 34.6k rpm with a SW40 rotor (Beckman Coulter, USA) for at least 16 h at 4°C. Fractions containing 2*YL*F_1F_o as judged by BN-PAGE (Wittig et al., 2006) were collected and loaded on a POROS GoPure HQ 50 anion exchange column (Life Technologies, USA) equilibrated with Buffer B (Buffer A with 0.1% (w/v) digitonin) and gradually eluted with Buffer C (Buffer B with 1 M NaCl) using an ÄKTAexplorer chromatography system (GE Healthcare, USA). Fractions containing ATP synthase dimers were collected and concentrated to 0.5 ml (Amicon Ultra-15 centrifugal filters with 100 kDa molecular mass cutoff) and loaded onto a 16/300 Superose 6 gel filtration column (GE Healthcare, USA) equilibrated with Buffer D (Buffer A with 0.05% (w/v) digitonin). Fractions eluting at a retention volume of 10–11 ml were collected and used for further analysis. Protein concentrations were determined with a BCA Protein Assay Kit (Thermo Scientific, D). Purification of the F_1F_o-ATP synthase monomer was carried out as described above, except that digitonin was replaced by 0.1% DDM during all purification steps and the gel filtration step was omitted.

ATPase activity measurements. ATP hydrolysis activity of 2*YL*F_1F_o was measured by an ATP-regenerating assay (Cook et al., 2003). Samples of 15 µg 2*YL*F_1F_o (0.5 mg/ml) were measured in 1 ml reaction volumes. 100 µg/ml yeast polar lipid extract and 12 µg/ml cardiolipin (Avanti, USA) was added and incubated overnight. To remove degradation products, 2*YL*F_1F_o was again applied to a Superose 6 column equilibrated

with Buffer E (Buffer D with yeast polar lipid and cardiolipin). ATP hydrolysis was inhibited by adding oligomycin (0.5 mg/ml in DMSO) to a final concentration of 5 µg/ml and incubation for at least 30 min. 1*YL*F_1F_o monomers were generated by adding DDM (20% (w/v) in water) to a final concentration of 1% (w/v) and 30 min incubation. Complete dissociation of the dimer was confirmed by BN-PAGE and electron microscopy of negatively stained samples.

Single particle cryo-EM. 3 µl of a 2-3 mg/ml 2*YL*F_1F_o (or 1*YL*F_1F_o) solution in 0.05% (w/v) digitonin [or 0.1% (w/v) DDM] was applied to R2/2 holey carbon grids (Quantifoil, DE) and plunge-frozen in liquid ethane using a Vitrobot (FEI) at 100% humidity after blotting for 7 to 9 s. Dose-fractionated 6 s movies of 30 frames with an electron dose of 0.6 electrons per $Å^2$ per frame were recorded on a Gatan K2 direct electron detector in a JEOL 3200 FSC electron microscope with an in column energy filter operated at zero-loss mode at 300 kV at 1.5–3.5 µm defocus, using a nominal magnification of 20,000x. The calibrated pixel size at was 1.63 Å.

Image processing and map interpretation. Global beam-induced motion was corrected by movie frame processing (Li et al., 2013). The contrast transfer function for each image was determined using CTFFIND3 (Mindell and Grigorieff, 2003) within the RELION-1.3 workflow (Scheres, 2012). 50,118 particles were picked manually with EMAN boxer (Ludtke et al., 1999). A sub-tomogram average of the *Saccharomyces cerevisiae* ATP synthase dimer EMD-2161 (Davies et al., 2012), obtained from tomographic volumes of mitochondrial membranes, was used as an initial reference for refinement in RELION-1.3 (Scheres, 2012). Two-dimensional classification of picked particles was performed in RELION-1.3. 2,774 particles were discarded. Three-dimensional classification of remaining particles was performed to identify structural flexibility and heterogeneity. A total of 38,679 particles belonging to three classes were combined for further processing. Individual frames were B-factor weighted and movements of individual particles were reversed by movie frame correction in RELION-1.3 (Scheres, 2014). A C2-averaged volume was calculated from particles in frames 1–20. A B-factor of -200 $Å^2$ for map sharpening was determined using the modulation transfer function of the K2 detector. To improve the resolution of individual sub-volumes during 3D refinement, customized soft edge masks for the F_1F_o monomer and the F_o dimer were generated using Chimera and

EMAN2 (Pettersen et al., 2004; Tang et al., 2007). Masks were used in the final iteration steps without imposing symmetry. Gold-standard Fourier shell correlations were calculated from two independently refined data sets to determine the resolution of the complete volume and sub-areas of the structure. For the latter, different regions of two independently refined maps were soft-masked and the resolution determined by Fourier shell correlations using EMAN2 (Tang et al., 2007). Models were fitted with Chimera (Pettersen et al., 2004).

Analysis of conformational states within the EM map. All pre-aligned particles were 3D-classified without symmetry, with an angular sampling interval of 1.8 degrees and local angular search range of +/-5 degrees in RELION-1.3. To analyze each monomer individually we repeated the 3D classification and applied a monomer mask. The classification quickly converged to three classes [named: *subclass 1*: 5,621 (15.3%), *subclass 2*: 16,671 (45.5%) and *subclass 3*: 14,379 (39.2%) particles] (**Figure S3D-G**). Next, we reconstructed 3D maps from all particles in each class using the 40 Å low-pass filtered maps of the corresponding 3D classes as new initial models.

Crystallization, X-ray data collection and structure determination of the *Y. lipolytica* F_1c_{10} subcomplex. Purified 1*YL*F_1F_o at a protein concentration of 15 mg/ml was supplemented with 1.0% (w/v) 3-([3-cholamidopropyl]-dimethylammonio)-2-hydroxy-1-propanesulfonate (CHAPSO, Hampton Research, USA). 1 µl of the protein solution was then mixed with 1 µl of 100 mM Tris-HCl with a pH adjusted to 8.0 and 23% (v/v) of PEG 400 (Sigma-Aldrich, USA). Crystallization plates were incubated at 18°C. Cuboid crystals were obtained after 14 days. The crystals were harvested and cryo-cooled in liquid nitrogen after dipping into cryo-protectant solution containing reservoir solution supplemented with 15% (v/v) glycerol. Datasets were collected at 100 K at the beamline PX-II X10SA (Swiss Light Source, Villigen, CH). Diffraction data were indexed and integrated with XDS and scaled with XSCALE (Kabsch, 1993). For data processing statistics see **Table 1**.

The structure was determined by molecular replacement with PHASER (McCoy, 2007) using a search model created by SWISS-MODEL (Biasini et al., 2014) (all-atom rmsd to final model: 1.36 Å) based on the *S. cerevisiae* F_1c_{10} complex (PDB: 2XOK) (Stock et al., 1999). The *c*-ring was subsequently exchanged by a high-resolution yeast model

(PDB: 4F4S) and mutated manually to the *Y. lipolytica* sequence. Following an initial Autobuild run, iterative cycles of manual model building and refinement were performed with COOT (Emsley et al., 2010) and PHENIX (Afonine et al., 2012) to final R_{work}/R_{free} values of 0.274/0.305 at 3.50 Å resolution. The quality of the model was validated with MolProbity (Chen et al., 2010). Refinement and validation statistics are summarized in **Table 1**. The refinement resulted in unambiguous electron density maps for all subunits [α_E 49-533, α_{TP} 49-428 and 435-533, α_{DP} 49-533, β_E 39-506, β_{TP} 37-506, β_{DP} 37-506, γ23-116 and 120-292, δ15-94 and 101-133, ε16-residue poly-Ala model, and *c*1-76 (1 subunit), *c*1-75 (2 subunits), *c*2-76 (2 subunits), *c*2-75 (3 subunits), *c*2-74 (1 subunit), or *c*2-73 (1 subunit). Ramachandran plots indicated excellent stereochemistry with 94.94% of the non-glycine and non-proline residues found in the most-favored region and 0.15% outliers, as determined by Molprobity (Chen et al., 2010).

Structural alignments and rmsd calculations. Structures were superposed using the align function in PyMOL (The PyMOL Molecular Graphics System, Schrödinger, LLC.), which performs a sequence alignment before superposition. RMSD values were calculated for backbone atoms only to avoid bias from sequence similarity. Rotational states were analyzed by superposing structures on their entire γ-subunits (chain G), to optimize the fit over all atoms, before comparing the positions of the P-loops of the β_{DP} sites (chain D, residues 160-173 for *S. cerevisiae*, 159-172 for *Bos taurus* and 191-204 for *Y. lipolytica* structures).

Figures were generated with PyMOL (The PyMOL Molecular Graphics System, Schrödinger, LLC.) and USCF Chimera (Pettersen et al., 2004). The amino acid alignment figures were created using ESPript (Robert and Gouet, 2014). Movies were prepared with MORPHINATOR (Karlsen and Bublitz, 2016) and Sequimago scripts http://www.macupdate.com/app/mac/28164/sequimago.

Crosslink analysis - consistent crosslink distances. To validate the assignment of helix densities in the *Y. lipolytica* F_o stator subcomplex to subunits *a, b, 8, f* and *d*, we compared our model to crosslink distances reported for the F_o complexes of the closely related yeast *S. cerevisiae* or of *E. coli* (DeLeon-Rangel et al., 2013; Jiang and

Fillingame, 1998; Schwem and Fillingame, 2006; Stephens et al., 2003) (**Figure S6** and **Table S3**). Note that the crosslinks described for *E. coli* ATP synthase (Jiang and Fillingame, 1998) are also found in the *I. tartaricus* Na^+-ATP synthase (Vorburger et al., 2008). Another recently described crosslink (Lee et al., 2015) between subunits *e* and *f* also supports our subunit assignment. Sequence alignments to identify homologous residue positions in the *Y. lipolytica* dimer model closely matched all reported Cys-substitution crosslinks with the exception of residue *c*Leu53 of the c_{10} ring. Residues Val111 and Val112 of subunit *a* could not be assigned since both are located in the loop between *a*H3 and *a*H4. This loop is not resolved in our map, but is expected to be in close proximity to Ser55 of subunit *b* (**Figure S5**).

Supplementary References

Abrahams, J.P., Buchanan, S.K., Van Raaij, M.J., Fearnley, I.M., Leslie, A.G.W., and Walker, J.E. (1996). The structure of bovine F_1-ATPase complexed with the peptide antibiotic efrapeptin. Proc Natl Acad Sci USA *93*, 9420-9424.

Abrahams, J.P., Leslie, A.G.W., Lutter, R., and Walker, J.E. (1994). Structure at 2.8 Å resolution of F_1-ATPase from bovine heart mitochondria. Nature *370*, 621-628.

Afonine, P.V., Grosse-Kunstleve, R.W., Echols, N., Headd, J.J., Moriarty, N.W., Mustyakimov, M., Terwilliger, T.C., Urzhumtsev, A., Zwart, P.H., and Adams, P.D. (2012). Towards automated crystallographic structure refinement with phenix.refine. Acta Crystallogr D Biol Crystallogr *68*, 352-367.

Arsenieva, D., Symersky, J., Wang, Y., Pagadala, V., and Mueller, D.M. (2010). Crystal structures of mutant forms of the yeast F_1 ATPase reveal two modes of uncoupling. J Biol Chem *285*, 36561-36569.

Bason, J.V., Montgomery, M.G., Leslie, A.G.W., and Walker, J.E. (2014). Pathway of binding of the intrinsically disordered mitochondrial inhibitor protein to F_1-ATPase. Proc Natl Acad Sci USA *111*, 11305-11310.

Bason, J.V., Montgomery, M.G., Leslie, A.G.W., and Walker, J.E. (2015). How release of phosphate from mammalian F_1-ATPase generates a rotary substep. Proc Natl Acad Sci USA *112*, 6009-6014.

Biasini, M., Bienert, S., Waterhouse, A., Arnold, K., Studer, G., Schmidt, T., Kiefer, F., Cassarino, T.G., Bertoni, M., Bordoli, L., *et al.* (2014). SWISS-MODEL: modelling protein tertiary and quaternary structure using evolutionary information. Nucleic Acids Res *42*, W252-258.

Bowler, M.W., Montgomery, M.G., Leslie, A.G.W., and Walker, J.E. (2006). How azide inhibits ATP hydrolysis by the F-ATPases. Proc Natl Acad Sci USA *103*, 8646-8649.

Bowler, M.W., Montgomery, M.G., Leslie, A.G.W., and Walker, J.E. (2007). Ground state structure of F_1-ATPase from bovine heart mitochondria at 1.9 Å resolution. J Biol Chem *282*, 14238-14242.

Boyer, P.D. (1997). The ATP synthase - a splendid molecular machine. Annu Rev Biochem *66*, 717-749.

Braig, K., Menz, R.I., Montgomery, M.G., Leslie, A.G.W., and Walker, J.E. (2000). Structure of bovine mitochondrial F_1-ATPase inhibited by Mg^{2+} ADP and aluminium fluoride. Structure *8*, 567-573.

Cain, B.D. (2000). Mutagenic analysis of the F_0 stator subunits. J Bioenerg Biomembr *32*, 365-3671.

Chen, V.B., Arendall, W.B. 3rd, Headd, J.J., Keedy, D.A., Immormino, R.M., Kapral, G.J., Murray, L.W., Richardson, J.S., and Richardson, D.C. (2010). MolProbity: all-atom structure validation for macromolecular crystallography. Acta Crystallogr D Biol Crystallogr *66*, 12-21.

Cook, G.M., Keis, S., Morgan, H.W., von Ballmoos, C., Matthey, U., Kaim, G., and Dimroth, P. (2003). Purification and biochemical characterization of the F_1F_o-ATP synthase from thermoalkaliphilic *Bacillus* sp. strain TA2.A1. J Bacteriol *185*, 4442-4449.

Dautant, A., Velours, J., and Giraud, M.F. (2010). Crystal structure of the Mg.ADP-inhibited state of the yeast F_1c_{10}-ATP synthase. J Biol Chem *285*, 29502-29510.

Davies, K.M., Anselmi, C., Wittig, I., Faraldo-Gómez, J.D., and Kühlbrandt, W. (2012). Structure of the yeast F_1F_o-ATP synthase dimer and its role in shaping the mitochondrial cristae. Proc Natl Acad Sci USA *109*, 13602-13607.

DeLeon-Rangel, J., Ishmukhametov, R.R., Jiang, W., Fillingame, R.H., and Vik, S.B. (2013). Interactions between subunits *a* and *b* in the rotary ATP synthase as determined by cross-linking. FEBS Lett *587*, 892-897.

Emsley, P., Lohkamp, B., Scott, W.G., and Cowtan, K. (2010). Features and development of Coot. Acta Crystallogr D Biol Crystallogr *66*, 486-501.

Eya, S., Maeda, M., and Futai, M. (1991). Role of the carboxy terminal region of H^+-ATPase (F_0F_1) a subunit from *Escherichia coli*. Arch Biochem Biophys *284*, 71-77.

Giraud, M.F., Paumard, P., Sanchez, C., Brethes, D., Velours, J., and Dautant, A. (2012). Rotor architecture in the yeast and bovine F_1-c-ring complexes of F-ATP synthase. J Struct Biol *177*, 490-497.

Gledhill, J.R., Montgomery, M.G., Leslie, A.G.W., and Walker, J.E. (2007). How the regulatory protein, IF1, inhibits F_1-ATPase from bovine mitochondria. Proc Natl Acad Sci USA *104*, 15671-15676.

Hunte, C., Zickermann, V., and Brandt, U. (2010). Functional modules and structural basis of conformational coupling in mitochondrial complex I. Science *329*, 448-451.

Jiang, W., and Fillingame, R.H. (1998). Interacting helical faces of subunits *a* and *c* in the F_1F_0 ATP synthase of *Escherichia coli* defined by disulfide cross-linking. Proc Natl Acad Sci USA *95*, 6607-6612.

Kabaleeswaran, V., Puri, N., Walker, J.E., Leslie, A.G.W., and Mueller, D.M. (2006). Novel features of the rotary catalytic mechanism revealed in the structure of yeast F_1 ATPase. EMBO J *25*, 5433-5442.

Kabaleeswaran, V., Shen, H., Symersky, J., Walker, J.E., Leslie, A.G.W., and Mueller, D.M. (2009). Asymmetric structure of the yeast F_1 ATPase in the absence of bound nucleotides. J Biol Chem *284*, 10546-10551.

Kabsch, W. (1993). Automatic processing of rotation diffraction data from crystals of initially unknown symmetry and cell constants. J Appl Cryst *26*, 795-800.

Kagawa, R., Montgomery, M.G., Braig, K., Leslie, A.G.W., and Walker, J.E. (2004). The structure of bovine F_1-ATPase inhibited by ADP and beryllium fluoride. EMBO J *23*, 2734-2744.

Karlsen, J.L., and Bublitz, M. (2016). How to compare, analyze and morph between crystal structures of different conformations: the P-type ATPase example. Met Mol Biol *1377*, 523-539.

Lee, J., Ding, S., Walpole, T.B., Holding, A.N., Montgomery, M.G., Fearnley, I.M., and Walker, J.E. (2015). Organization of subunits in the membrane domain of the bovine F-ATPase revealed by covalent cross-linking. J Biol Chem *290*, 13308-13320.

Li, X., Mooney, P., Zheng, S., Booth, C.R., Braunfeld, M.B., Gubbens, S., Agard, D.A., and Cheng, Y. (2013). Electron counting and beam-induced motion correction enable near-atomic-resolution single-particle cryo-EM. Nat Methods *10*, 584-590.

Lightowlers, R.N., Howitt, S.M., Hatch, L., Gibson, F., and Cox, G.B. (1987). The proton pore in *Escherichia coli* F_0F_1- ATPase: A requirement of arginine at position 210 of the a-subunit. Biochim Biophys Acta *894*, 399-406.

Liu, S., Charlesworth, T.J., Bason, J.V., Montgomery, M.G., Harbour, M.E., Fearnley, I.M., and Walker, J.E. (2015). The purification and characterization of ATP synthase complexes from the mitochondria of four fungal species. Biochem J *468*, 167-175.

Ludtke, S.J., Baldwin, P.R., and Chiu, W. (1999). EMAN: semiautomated software for high-resolution single-particle reconstructions. J Struct Biol *128*, 82-97.

McCoy, A.J. (2007). Solving structures of protein complexes by molecular replacement with Phaser. Acta Crystallogr D Biol Crystallogr *63*, 32-41.

Menz, R.I., Leslie, A.G.W., and Walker, J.E. (2001a). The structure and nucleotide occupancy of bovine mitochondrial F_1-ATPase are not influenced by crystallisation at high concentrations of nucleotide. FEBS Lett *494*, 11-14.

Menz, R.I., Walker, J.E., and Leslie, A.G.W. (2001b). Structure of bovine mitochondrial F_1-ATPase with nucleotide bound to all three catalytic sites: implications for the mechanism of rotary catalysis. Cell *106*, 331-341.

Mindell, J.A., and Grigorieff, N. (2003). Accurate determination of local defocus and specimen tilt in electron microscopy. J Struct Biol *142*, 334-347.

Morales-Rios, E., Montgomery, M.G., Leslie, A.G.W., and Walker, J.E. (2015). Structure of ATP synthase from *Paracoccus denitrificans* determined by X-ray crystallography at 4.0 Å resolution. Proc Natl Acad Sci USA *112*, 13231-13236.

Orriss, G.L., Leslie, A.G.W., Braig, K., and Walker, J.E. (1998). Bovine F_1-ATPase covalently inhibited with 4-chloro-7-nitrobenzofurazan: the structure provides further support for a rotary catalytic mechanism. Structure *6*, 831-837.

Pettersen, E.F., Goddard, T.D., Huang, C.C., Couch, G.S., Greenblatt, D.M., Meng, E.C., and Ferrin, T.E. (2004). UCSF Chimera--a visualization system for exploratory research and analysis. J Comput Chem *25*, 1605-1612.

Rees, D.M., Leslie, A.G.W., and Walker, J.E. (2009). The structure of the membrane extrinsic region of bovine ATP synthase. Proc Natl Acad Sci USA *106*, 21597-21601.

Rees, D.M., Montgomery, M.G., Leslie, A.G.W., and Walker, J.E. (2012). Structural evidence of a new catalytic intermediate in the pathway of ATP hydrolysis by F_1-ATPase from bovine heart mitochondria. Proc Natl Acad Sci USA *109*, 11139-11143.

Robert, X., and Gouet, P. (2014). Deciphering key features in protein structures with the new ENDscript server. Nucleic Acids Res *42*, W320-324.

Robinson, G.C., Bason, J.V., Montgomery, M.G., Fearnley, I.M., Mueller, D.M., Leslie, A.G.W., and Walker, J.E. (2013). The structure of F_1-ATPase from *Saccharomyces cerevisiae* inhibited by its regulatory protein IF1. Open Biol *3*, 120164.

Schägger, H., and von Jagow, G. (1987). Tricine-sodium dodecyl sulfate-polyacrylamide gel electrophoresis for the separation of proteins in the range from 1 to 100 kDa. Anal Biochem *166*, 368-379.

Scheres, S.H. (2012). RELION: implementation of a Bayesian approach to cryo-EM structure determination. J Struct Biol *180*, 519-530.

Scheres, S.H. (2014). Beam-induced motion correction for sub-megadalton cryo-EM particles. eLife *3*, e03665.

Schwem, B.E., and Fillingame, R.H. (2006). Cross-linking between helices within subunit *a* of *Escherichia coli* ATP synthase defines the transmembrane packing of a four-helix bundle. J Biol Chem *281*, 37861-37867.

Stephens, A.N., Khan, M.A., Roucou, X., Nagley, P., and Devenish, R.J. (2003). The molecular neighborhood of subunit *8* of yeast mitochondrial F_1F_0-ATP synthase probed by cysteine scanning mutagenesis and chemical modification. J Biol Chem *278*, 17867-17875.

Stock, D., Leslie, A.G.W., and Walker, J.E. (1999). Molecular architecture of the rotary motor in ATP synthase. Science *286*, 1700-1705.

Tang, G., Peng, L., Baldwin, P.R., Mann, D.S., Jiang, W., Rees, I., and Ludtke, S.J. (2007). EMAN2: an extensible image processing suite for electron microscopy. J Struct Biol *157*, 38-46.

van Raaij, M.J., Abrahams, J.P., Leslie, A.G.W., and Walker, J.E. (1996). The structure of bovine F_1-ATPase complexed with the antibiotic inhibitor aurovertin B. Proc Natl Acad Sci USA *93*, 6913-6917.

Vorburger, T., Ebneter, J.Z., Wiedenmann, A., Morger, D., Weber, G., Diederichs, K., Dimroth, P., and von Ballmoos, C. (2008). Arginine-induced conformational change in the *c*-ring/*a*-subunit interface of ATP synthase. FEBS J *275*, 2137-2150.

Watt, I.N., Montgomery, M.G., Runswick, M.J., Leslie, A.G.W., and Walker, J.E. (2010). Bioenergetic cost of making an adenosine triphosphate molecule in animal mitochondria. Proc Natl Acad Sci USA *107*, 16823-16827.

Wittig, I., Braun, H.P., and Schägger, H. (2006). Blue native PAGE. Nat Protoc *1*, 418-428.

Zhou, A., Rohou, A., Schep, D.G., Bason, J.V., Montgomery, M.G., Walker, J.E., Grigorieff, N., and Rubinstein, J.L. (2015). Structure and conformational states of the bovine mitochondrial ATP synthase by cryo-EM. eLife *4*.
