# Supplementary figures and images for: Structure of a Complete ATP Synthase Dimer Reveals the Molecular Basis of Inner Mitochondrial Membrane Morphology
Source: Mol Cell. 2016 Aug 4;63(3):445–56. doi: 10.1016/j.molcel.2016.05.037 (PMC4980432; doi:10.1016/j.molcel.2016.05.037)

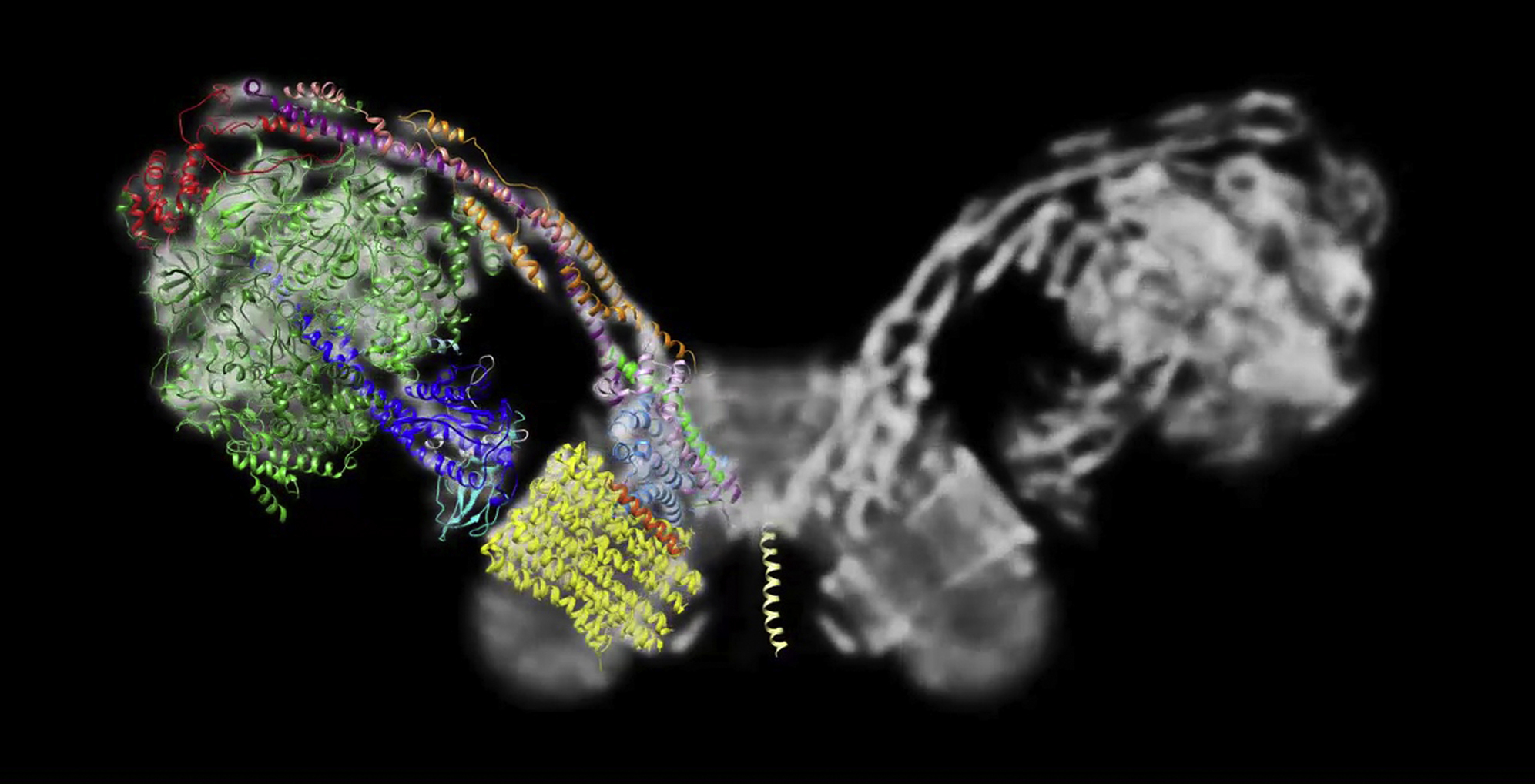

Supplement: Movie S1. Y. lipolytica ATP Synthase Dimer EM Map — Related to Figure 2. Cartoon model of all assigned protein subunits is shown in one of the two monomers. Color code is as in Figure 2. [file mmc2.jpg]

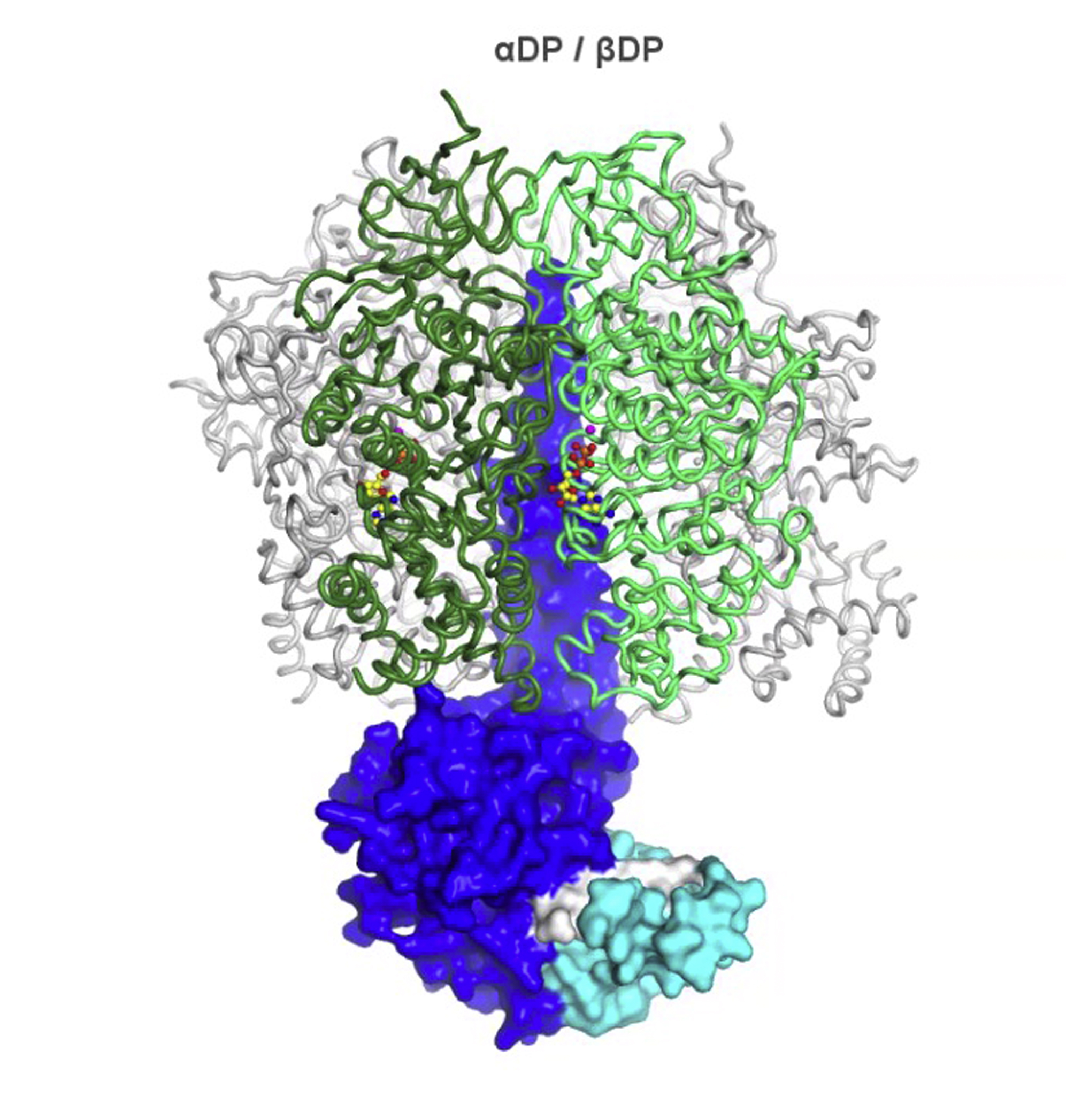

Supplement: Movie S2. Morphed Conformational Changes in Y. lipolytica F1-ATP Synthase — Related to Figure 1. Animation based on a morph between the three different conformational states observed in the Y. lipolytica F1c10 crystal structure. Subunit representations and coloring as follows: α, dark green or dark gray ribbon; β, light green or light gray ribbon; γ, blue surface; δ, cyan surface; ε, white surface. MgADP is represented as stick-ball model with C, N, O, P, and Mg colored in yellow, blue, red, orange, and magenta, respectively. The corner points for the morphs were generated by triplicating the entire molecule and then superposing the αDP subunit of the first copy and the αTP subunit of the second copy on the αE subunit of the original structure. [file mmc3.jpg]

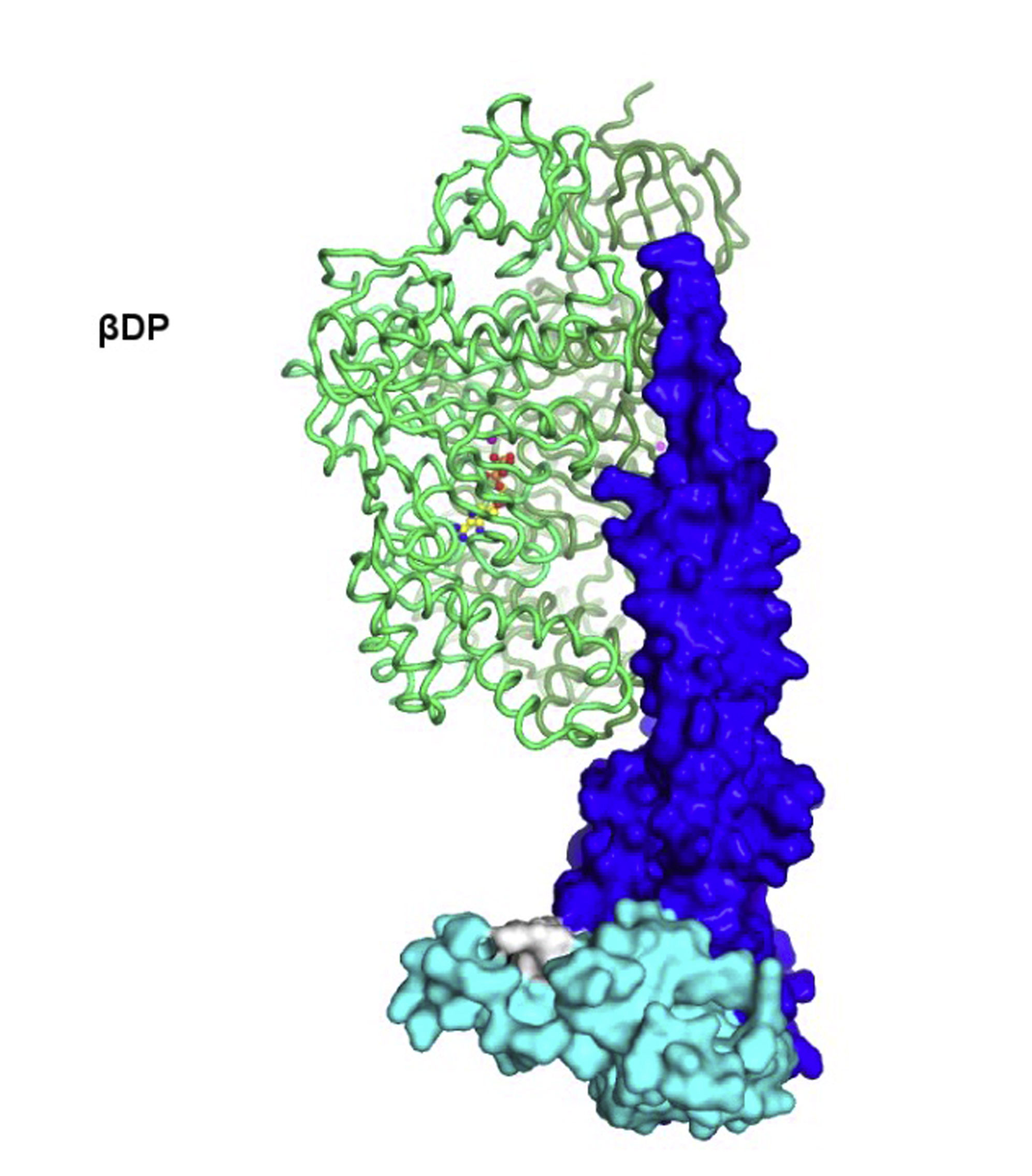

Supplement: Movie S3. Morphed Conformational Changes in the α/β Heterodimer — Related to Figure 1. Alternative view on the morphed animation shown also in Movie S2. For clarity, only one catalytic α/β heterodimer is shown, together with the rotating stalk containing subunits γ, δ, and ε. Representations and coloring as in Movie S2. [file mmc4.jpg]

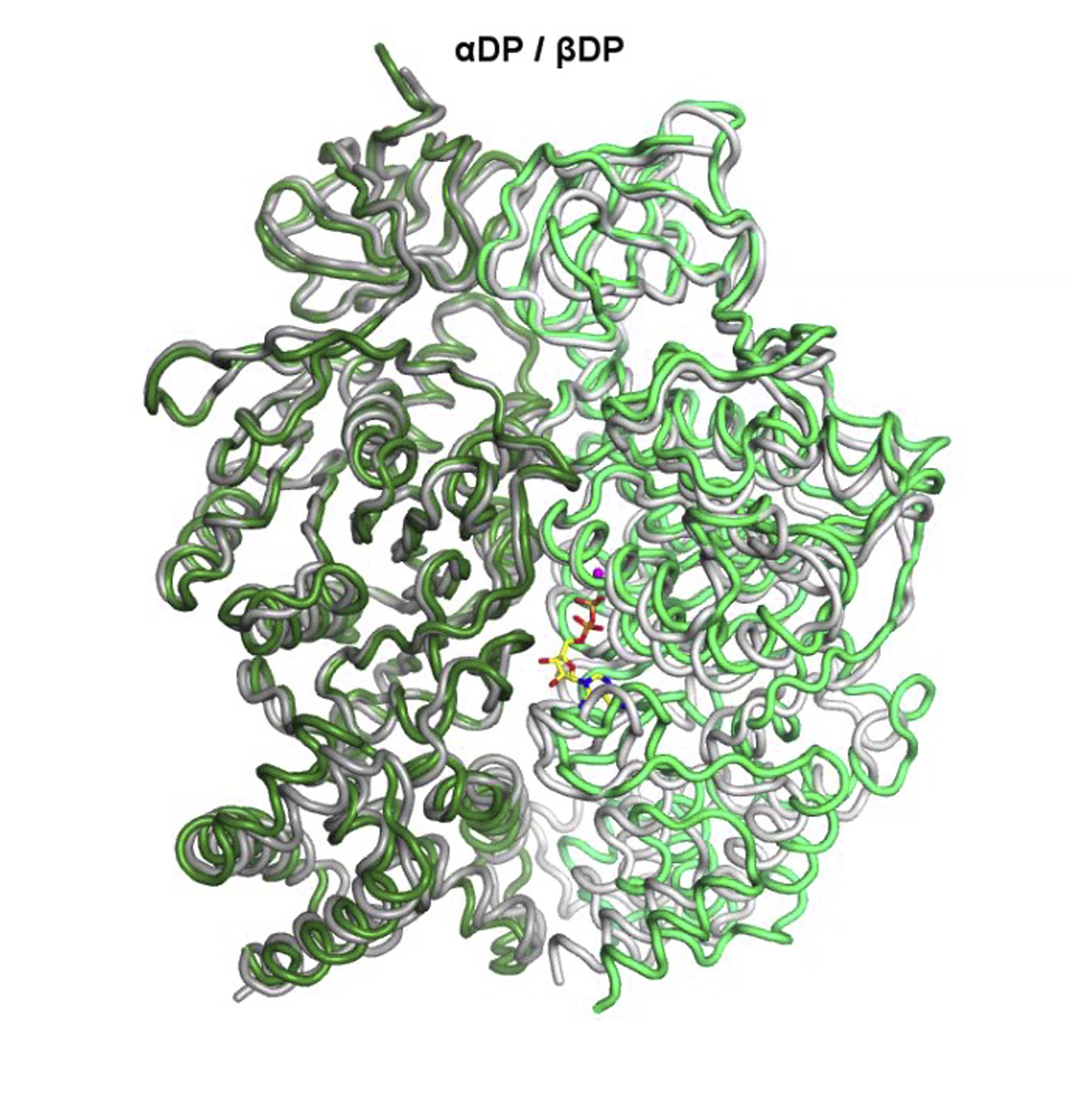

Supplement: Movie S4. Comparison of Yeast and Bovine ATP Synthase — Related to Figure 1. Superposed morphs of a catalytic α/β heterodimer from B. taurus (PDB: 1BMF; α, dark gray; β, light gray) (Abrahams et al., 1994) and Y. lipolytica (this work; α, dark green; β, light green). The α subunits were used as reference for both morphing and superposition. The conformations of the β subunits are most divergent in the βDP states, and more similar in the βTP and βE states. Also, the bovine structure undergoes larger overall conformational changes than the Y. lipolytica structure. [file mmc5.jpg]

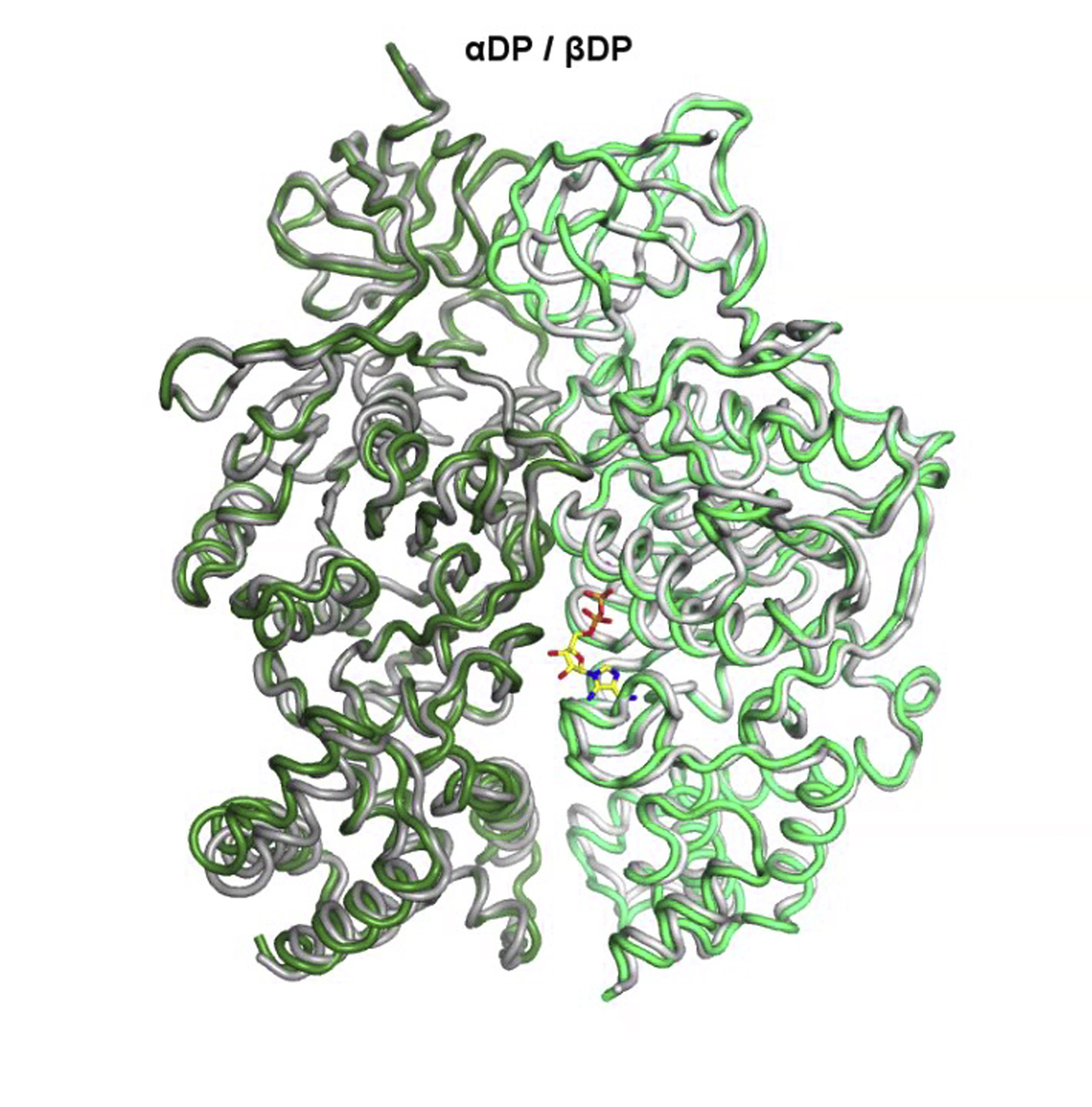

Supplement: Movie S5. Comparison of S. cerevisiae and Y. lipolytica α/β Heterodimers — Related to Figure 1. Superposed morphs of a catalytic α/β heterodimer from S. cerevisiae (PDB: 2HLD; α, dark gray; β, light gray) (Kabaleeswaran et al., 2006) and Y. lipolytica (this work; α, dark green; β, light green). The α subunits were used as reference for both morphing and superposition. [file mmc6.jpg]
