# Supplementary material for: Structure of a Complete ATP Synthase Dimer Reveals the Molecular Basis of Inner Mitochondrial Membrane Morphology
Source: Mol Cell. 2016 Aug 4;63(3):445–56. doi: 10.1016/j.molcel.2016.05.037 (PMC4980432; doi:10.1016/j.molcel.2016.05.037)
Supplement: Document S2. Article plus Supplemental Information [file mmc7.pdf]

Molecular Cell

Article

Structure of a Complete ATP Synthase Dimer Reveals the Molecular Basis of Inner Mitochondrial Membrane Morphology

Graphical Abstract

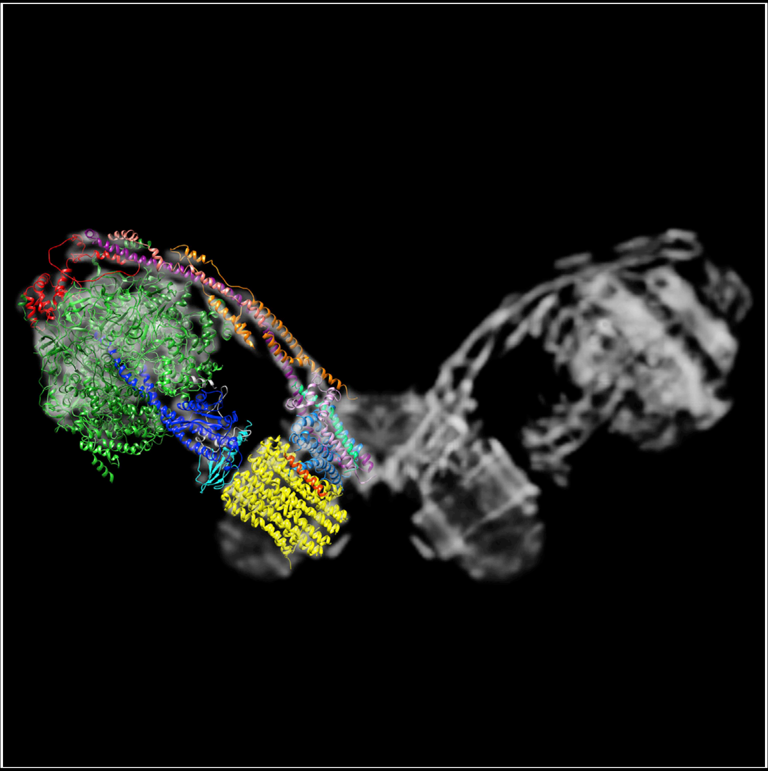

Authors

Alexander Hahn, Kristian Parey, Maike Bublitz, ..., Janet Vonck, Werner Kühlbrandt, Thomas Meier

Correspondence

werner.kuehlbrandt@biophys.mpg.de (W.K.), t.meier@imperial.ac.uk (T.M.)

In Brief

ATP synthases are complex macromolecular machines that supply most of the ATP in cells. Hahn et al. present the structure of a complete ATP synthase dimer, which provides insights into both the mechanism of these nanomotors and how they cause membrane bending to form cristae in the inner mitochondrial membrane.

Highlights

- Cryo-EM structure of a yeast F_1F_o-ATP synthase dimer
- Inhibitor-free X-ray structure of the F_1 head and rotor complex
- Mechanism of ATP generation by rotary catalysis
- Structural basis of cristae formation in the inner mitochondrial membrane

Accession Numbers

5FL7

Hahn et al., 2016, Molecular Cell *63*, 445–456
August 4, 2016 © 2016 The Authors. Published by Elsevier Inc.
http://dx.doi.org/10.1016/j.molcel.2016.05.037

CellPress

Structure of a Complete ATP Synthase Dimer Reveals the Molecular Basis of Inner Mitochondrial Membrane Morphology

Alexander Hahn,[1] Kristian Parey,[1] Maike Bublitz,[2,4] Deryck J. Mills,[1] Volker Zickermann,[3] Janet Vonck,[1] Werner Kühlbrandt,[1,*] and Thomas Meier[1,5,*]
[1]Department of Structural Biology, Max Planck Institute of Biophysics, Max-von-Laue-Str. 3, 60438 Frankfurt am Main, Germany
[2]Institute of Biochemistry
[3]Institute of Biochemistry II, Medical School
Goethe University Frankfurt, Max-von-Laue-Str. 9, 60438 Frankfurt am Main, Germany
[4]Present address: Department of Biochemistry, University of Oxford, South Parks Road, Oxford OX1 3QU, UK
[5]Present address: Department of Life Sciences, Imperial College London, Exhibition Road, London SW7 2AZ, UK
*Correspondence: werner.kuehlbrandt@biophys.mpg.de (W.K.), t.meier@imperial.ac.uk (T.M.)
http://dx.doi.org/10.1016/j.molcel.2016.05.037

SUMMARY

We determined the structure of a complete, dimeric F_1F_o-ATP synthase from yeast *Yarrowia lipolytica* mitochondria by a combination of cryo-EM and X-ray crystallography. The final structure resolves 58 of the 60 dimer subunits. Horizontal helices of subunit *a* in F_o wrap around the *c*-ring rotor, and a total of six vertical helices assigned to subunits *a, b, f, i*, and *8* span the membrane. Subunit *8* (*A6L* in human) is an evolutionary derivative of the bacterial *b* subunit. On the lumenal membrane surface, subunit *f* establishes direct contact between the two monomers. Comparison with a cryo-EM map of the F_1F_o monomer identifies subunits *e* and *g* at the lateral dimer interface. They do not form dimer contacts but enable dimer formation by inducing a strong membrane curvature of ~100°. Our structure explains the structural basis of cristae formation in mitochondria, a landmark signature of eukaryotic cell morphology.

INTRODUCTION

The mitochondrial F_1F_o-ATP synthase produces most of the ATP in the cell by rotary catalysis and plays a crucial role in severe human neurodegenerative disorders (Kucharczyk et al., 2009). The proton motive force (pmf) across the inner membrane drives the *c*-ring rotor in the membrane-embedded F_o subcomplex, generating the torque that powers a sequence of conformational changes in the membrane-extrinsic F_1 subcomplex, resulting in ATP generation (Abrahams et al., 1994; Boyer, 1997; Noji et al., 1997). The F_o subcomplex is connected to F_1 by the central stalk, which transmits torque to the catalytic head, and the peripheral stalk, which acts as a stator to prevent idle rotation of the F_1 head with the *c*-ring.

Dimers of the ATP synthase shape the inner mitochondrial membrane and mediate cristae formation (Davies et al., 2012; Paumard et al., 2002). The ATP synthase forms rows of V-shaped dimers along the highly curved edges of inner membrane cristae (Strauss et al., 2008). The dimer angle is 86° in yeasts and metazoans, but different in mitochondria of plants (Davies et al., 2011) and algae (Allegretti et al., 2015). Recently, the complete structure of the dimeric mitochondrial ATP synthase of the chlorophyll-less green alga *Polytomella* sp. was reported at 6.2 Å resolution, revealing the unexpected feature of a horizontal four-helix bundle in the *a* subunit of the F_o subcomplex (Allegretti et al., 2015). The long horizontal helices are conserved not only in mammalian mitochondria (Zhou et al., 2015) and bacteria (Morales-Rios et al., 2015) but also in the more distantly related V-type and A-type ATPases (Zhao et al., 2015), and are thus a fundamental feature common to all rotary ATPases (Kühlbrandt and Davies, 2016). Together with the *c*-ring rotor, the horizontal helices of subunit *a* create two aqueous half-channels on either side of the membrane (Allegretti et al., 2015; Kühlbrandt and Davies, 2016). The *c* subunits in the rotor ring bind and release protons as the ring rotates through the alternating hydrophobic environment of the lipid bilayer and the aqueous environment of the half-channels (Allegretti et al., 2015; Meier et al., 2011, 2005; Pogoryelov et al., 2010; Symersky et al., 2012), thereby generating the torque for ATP synthesis.

The recently reported structures include the dimeric form of an ATP synthase that has unusual peripheral stalks (Allegretti et al., 2015), and the monomer of the bovine complex (Zhou et al., 2015) as well as a bacterial ATP synthase (Morales-Rios et al., 2015), which both appear to be incomplete. There is currently no structure of an ATP synthase dimer that closely resembles the mammalian complex. Mitochondrial ATP synthases from yeasts have a subunit composition very similar to the mammalian (human) ATP synthase and form the same V-shaped dimers. By a combination of cryoelectron microscopy (cryo-EM) and X-ray crystallography, we have obtained the structure of the complete ATP synthase dimer from the aerobic, genetically accessible yeast *Yarrowia lipolytica*, in which ATP synthase dimers were previously reported (Davies et al., 2011; Nübel et al., 2009). The combined maps resolve 58 of the 60 known

This is an open access article under the CC BY license (http://creativecommons.org/licenses/by/4.0/).

Table 1. Table of Crystallography

	Y. lipolytica F_1c_{10}
Data Collection	
Wavelength (Å)	1.008
Space group	$P2_12_12$
Cell dimensions: *a*, *b*, *c* (Å)	169.5, 182.2, 193.0
Cell dimensions: α, β, γ (°)	90, 90, 90
Resolution (Å)	49.19–3.50 (3.60–3.50)[a]
Total reflections	1,477,286 (119,821)[a]
Unique reflections	75,882 (6,046)[a]
R_{merged}	18.2 (>100)[a]
$I / \sigma(I)$	9.84 (0.61)[a]
Completeness (%)	99.99 (100.0)[a]
Redundancy	19.5 (19.8)[a]
Refinement	
Resolution (Å)	3.50
R_{work} / R_{free} (%)	27.39 / 30.54
Wilson β factor	158
Average β factor (Å^2)	167
No. atoms	30,123
Protein	29,954
Ligands	152
Water	17[b]
RMSDs: bond lengths (Å)	0.006
RMSDs: bond angles (°)	0.883
PDB code	5FL7

[a]Values in parentheses are for highest-resolution shell.
[b]Water molecules coordinated to Mg^{2+} in the nucleotide binding sites (Figure S2E).

protein subunits and the inhibitor protein *IF1*. The structure reveals the previously unknown subunit architecture of the dimer interface in the membrane, thereby providing major new insights into mitochondrial membrane architecture.

RESULTS

Isolation and Biochemistry of *Yarrowia lipolytica* ATP Synthase Dimers

ATP synthase dimers from *Y. lipolytica* were purified from dodecylmaltoside (DDM)-solubilized mitochondrial membranes by centrifugation in a digitonin-containing glycerol gradient, followed by anion exchange chromatography. Two-dimensional gel electrophoresis and liquid chromatography-mass spectrometry (LC-MS) indicated that the 2*YL*F_1F_o (*Y. lipolytica* ATP synthase dimer) fraction contained all ATP synthase subunits, including *e*, *g*, and *k*, which are known as dimer specific (Arnold et al., 1998) (Figure S1; Table S1, available online). The DDM-purified monomeric *Y. lipolytica* ATP synthase (1*YL*F_1F_o) lacks subunits *e*, *g*, and *k*. The ATP hydrolysis activity of both 1*YL*F_1F_o and 2*YL*F_1F_o is ~2.25 U/mg. F_o is coupled to 95% and 75%, respectively, as determined by oligomycin inhibition. The lower percentage of coupled complexes in 2*YL*F_1F_o is most likely due to free F_1 subcomplexes and detergent in the dimer preparation (Figure S1A). The similarly high activities of 2*YL*F_1F_o and 1*YL*F_1F_o indicate that the two ATP synthase monomers within the dimer operate independently in ATP hydrolysis mode.

F_1c_{10} Crystal Structure

Crystals of the *YL*F_1c_{10} subcomplex were obtained from the 1*YL*F_1F_o complex. Whereas previous crystallographic studies of similar complexes (Giraud et al., 2012; Pagadala et al., 2011; Stock et al., 1999) used an excess of nucleotide substrates or inhibitors to trap functional states, we crystallized *YL*F_1c_{10} without any such additives to ensure similar conditions for crystallography and cryo-EM. The 3.5 Å X-ray structure of *YL*F_1c_{10} (Table 1; Figures S2A–S2D) reveals that all three non-catalytic α subunits bind Mg·ATP in their nucleotide sites. Of the three catalytic β subunits, one is empty (β_E), while both the β_{DP} and β_{TP} sites (Abrahams et al., 1994) contain Mg·ADP (Figures 1 and S2E).

Cryo-EM Structure of the *Yarrowia lipolytica* ATP Synthase Dimer

We determined the structure of the 2*YL*F_1F_o by single-particle cryo-EM (Figure 2A). After 2D and 3D classification, 38,679 particles were selected for reconstruction of a 3D map with C2 symmetry imposed. The central stalks of the two monomers include an angle of ~100°. Masking one monomer in the dimer during 3D refinement improved the resolution to 6.9 Å for the F_1 subcomplex and masking the F_o dimer improved it to 6.2 Å, as determined by gold-standard Fourier shell correlation (Figure S4). The long helices in the peripheral stalks and the F_o part of the stator are the best-resolved features (Movie S1). The resolution of the F_1c_{10} subcomplex in the cryo-EM map is slightly lower, due to minor variations in the dimer angle (Figures S3D and S3E) and to differences in rotational position of the rotor assembly. Further classification revealed that the position of the central stalk varies independently in both monomers (Figures S3F and S3G), confirming that the two ATP synthase assemblies in the dimer function independently, as already suggested by the similar ATPase hydrolysis activities of 1*YL*F_1F_o and 2*YL*F_1F_o.

Classification of the same dataset with one monomer in the dimer masked enabled us to distinguish three different rotational states of the F_1 head assembly, with two out of three positions favored, in which the positions of the central stalk differ by ~120° or 240° (Figures S3H and S3I). Interaction with the central stalk affects the nucleotide binding domains and C-terminal domains of the β subunits (Figure S3I). The three conformations show the three "Boyer states," open, loose, and tight (Boyer, 1997), of the *Y. lipolytica* complex as seen in the *YL*F_1c_{10} crystal structure (Movies S2 and S3), similar to the crystal structure of the bovine F_1 complex (Abrahams et al., 1994). The three states are trapped in energy wells, which stall the rotor in defined positions upon dissipation of the pmf by the membrane-solubilizing detergent.

In the most populated class (45% of the particles; subclass 2 in Figures S3H and S3I), a rod-like density protrudes from the $\alpha_{DP}\beta_{DP}$ pair close to the peripheral stalk (Figure 2D). This density superposes precisely on the inhibitor *IF1* in an X-ray structure of the bovine mitochondrial F_1F_o-ATP synthase with *IF1* bound (Gledhill et al., 2007). The presence of *IF1* in ATP synthases prepared from large-scale yeast fermentations is not unexpected,

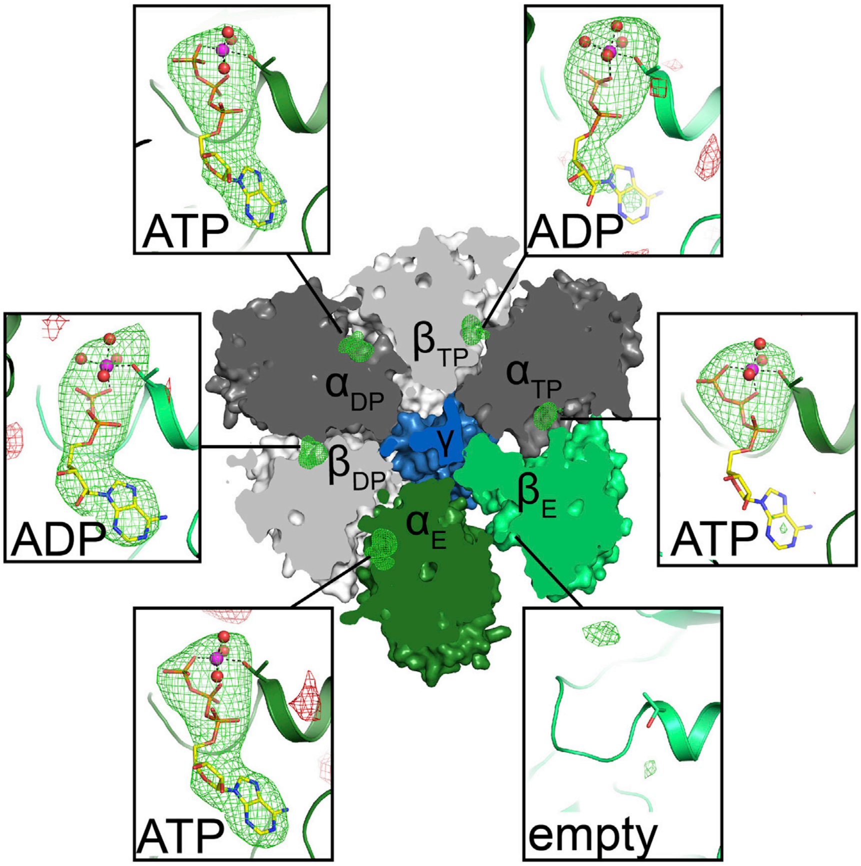

Figure 1. Nucleotide Binding Sites in *Yarrowia lipolytica* F_1c_{10}

Cross section through the F_1 X-ray structure shows the six nucleotide binding sites at the α/β subunit interfaces viewed from the matrix. Subunits α (dark green, dark gray) and β (light green, light gray) are arranged around the central γ subunit (blue). Green and red mesh indicates unbiased mF_{obs}-DF_{calc} nucleotide difference densities contoured at 3.0σ and -3.0σ, respectively. Boxed, close-up views of Walker A nucleotide binding motifs (cartoon) with αThr202, βThr195, and nucleotides in stick representation. Mg^{2+} ions with coordinated water molecules are shown as spheres. Atoms of C, N, O, P, and Mg are colored yellow, blue, red, orange, and magenta, respectively. Positive difference densities match Mg·ATP [·3 H_2O] in all three α sites, and Mg·ADP[·4 H_2O] in β_{DP} and β_{TP}. The β_E site is empty. The conformational flexibility of the β_E subunit is a possible cause of the weaker adenosine density of the ATP in the adjacent α_{TP} site. See Figure S3 for further difference maps after modeling various nucleotides in the α and β sites.

as oxygen concentration of these cultures can decrease, which reduces the matrix pH and triggers *IF1* binding, as observed with yeast grown on non-fermentable substrates (Satre et al., 1975). The fact that *IF1* is found in only one of the three classes is, however, surprising.

Peripheral Stalk

The peripheral stalk consists of several long, well-resolved α helices, which were traced without ambiguity (Figures 2A–2C). Homology models based on crystal structures of the bovine subunits *b*, *d*, and *OSCP* (Dickson et al., 2006; Rees et al., 2009) were fitted to the soluble sector of the *Y. lipolytica* peripheral stalk, which has the same subunit composition (Table S1). Subunit *h* has only 20% sequence identity to the equivalent bovine *F6* (Fujikawa et al., 2015), accounting for the observed structural differences. The overall curvature of the peripheral stalk differs from that in the bovine crystal structure, but resembles that in the cryo-EM map of the monomeric bovine complex (Baker et al., 2012), suggesting that crystal contacts affect stalk curvature. As in the bovine complex (Zhou et al., 2015), helices 1 and 5 of *OSCP* on the F_1 head are in contact with the N terminus of α_E (Rees et al., 2009). Two further close contacts are found at the N terminus of α_{TP}, which interacts with helices 4 and 5 of *OSCP*, and at the N terminus of α_{DP}, which intercalates between the peripheral stalk helices. The N terminus of this α subunit forms a previously unrecognized four-helix bundle with *b*, *h*, and the C terminus of *OSCP*, which positions the F_1 head and bonds it to the peripheral stalk (Figure 2B). The contacts in this interaction are mainly hydrophobic, except for those mediated by the conserved residues αGlu33 and αArg41. The *d* subunit interacts with the C terminus of the α_{DP} subunit, displacing it toward the peripheral stalk by 5 Å relative to the *YL*F_1c_{10} X-ray structure (Figure 2C). Below the F_1 head, peripheral stalk subunits *d* and *b* bend toward the central stalk. The density of subunit *b*, which is thought to have two trans-membrane helices at its N terminus (Figure S4), continues without interruption into the membrane.

Helix Assignment in the F_o Stator

The *Y. lipolytica* F_o stator subcomplex comprises the eight membrane protein subunits *a*, *b*, *e*, *f*, *g*, *i*, *k*, and *8*. The F_o part of each monomer contains ten well-defined α-helical densities enveloped by a detergent micelle that features the characteristic ~90° dimer membrane curvature (Davies et al., 2012) (Figure 2A). Six of these densities indicate trans-membrane α helices, numbered 1–6 in Figures 3A and 3B and assigned in Figure 3C. The loops connecting the helices are, for the most part, not visible at this resolution, but the helix segments can be identified on the basis of sequence comparison, secondary structure predictions, proximity, and known helix topology.

Subunit b

Helix 1 is the continuation of the peripheral stalk subunit *b* and is thus the second trans-membrane helix of *b.* Helix 2 is close to it and is the most likely candidate for the first trans-membrane helix of this subunit. The second-nearest helix 3 is too far for the short, six-residue loop connecting the two trans-membrane helices of subunit *b* (Figure S4A).

Subunit a

Sequence alignment of subunit *a* indicates a consistent pattern of seven characteristic consecutive protein regions (Figure S5A):

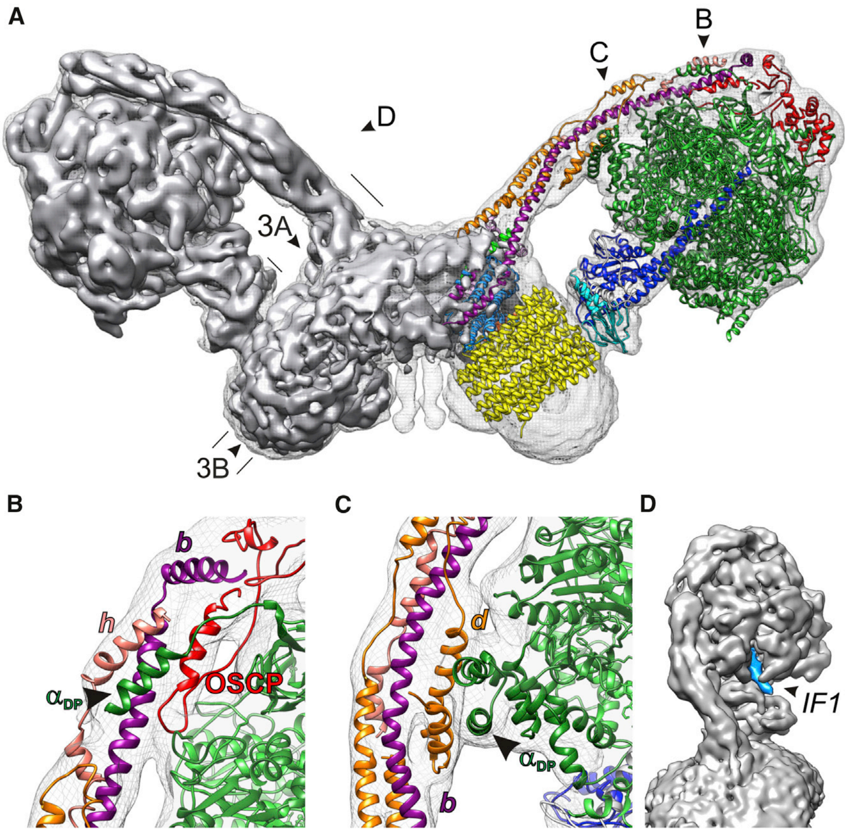

Figure 2. Cryo-EM Structure of the *Yarrowia lipolytica* F_1F_o-ATP Synthase Dimer

(A) Side view of the map (gray surface and volume). The monomer on the right was fitted in Coot (Emsley et al., 2010) with the X-ray structure of the *Y. lipolytica* F_1c_{10} complex and homology models of peripheral stalk subunits based on atomic models from the *B. taurus* outer stalk structures (PDB: 2WSS and 2CLY) (Figures 1 and S2E). Cross sections shown in Figures 3A and 3B and viewing directions for (B)–(D) are indicated.

(B and C) Detailed views of peripheral stalk subunit interactions as indicated in (A). (B) Upper and (C) lower section. Dark and light green, α and β subunits, respectively; light blue, subunit a; yellow, c_{10} ring; blue, subunit γ; cyan, subunit δ; light gray, subunit ε; purple, subunit *b*; orange, subunit *d*; salmon, subunit *h*; red, *OSCP*.

(D) The intrinsic inhibitor protein *IF1* (light blue) binds in the α/β_{DP} site proximal to the peripheral stalk. The overall map resolution of 7.8 Å in (A) improved upon masking to 6.9 Å for the F_1 complex (B–D). Movie S1 is a video of the rotating complete ATP synthase dimer.

(i) the hydrophilic N terminus; (ii) a ~20 residue hydrophobic stretch indicative of a trans-membrane helix; (iii) a region rich in hydrophilic and polar residues, prone to form an amphipathic helix (Figure S5B); (iv) two hydrophobic sequences separated by charged or polar side chains; (v) a region with several positively charged residues followed by (vi) a proline-rich region; and finally (vii) an extensive hydrophobic stretch with interspersed, highly conserved charged and polar residues.

We can assign region (iii), the amphipathic helix *a*H2, to the straight helix density on the matrix side just above the horizontal four-helix bundle (Figure 4). Region (ii), the trans-membrane helix of subunit *a*, which we refer to as *a*H1, would thus be helix density 3 in the map (Figures 3 and 4). The N-terminal region (i) of subunit *a* is small, is without clear predicted secondary structure, and has no discernible map density. Regions (iv) to (vii) are assigned to the two membrane-intrinsic helix hairpins of subunit *a* (which we refer to as *a*H3 to *a*H6) on the basis of their striking similarity to the same feature in the *Polytomella* dimer map (Allegretti et al., 2015). The assignment of the two shorter helices as *a*H3 and *a*H4 follows from their proximity to the amphipathic helix *a*H2 (Figure 4). The non-helical regions (v) and (vi) link the two helix hairpins, but only limited density is visible for them in the map. We assign the longest helix in the four-helix bundle, which follows the curve of the *c*-ring closely, to *a*H5 in the first half of region (vii), and the second helix in this hairpin to helix *a*H6 in the C-terminal half of this region (Figure 4). Helix *a*H5 contains the essential Arg182, which interacts with the protonatable *c*-ring glutamate (Cain, 2000; Eya et al., 1991; Lightowlers et al., 1987). Our assignment places this residue and a series of conserved charged or polar residues in the long horizontal hairpin at the subunit *a/c* interface (see Discussion). Our *a* subunit assignment is fully consistent with that of the bovine (Zhou et al., 2015) and *Paracoccus* ATP synthase (Morales-Rios et al., 2015), but the order of helices *a*H5 and *a*H6 with respect to the *Polytomella* assignment (Allegretti et al., 2015) is reversed.

Subunit 8

Helix 4 (green in Figure 3) has a short matrix extension with a slight kink toward the *c*-ring. We assign this density to the small, 48-residue subunit *8* (Figure S4B). Subunit *8* has a conserved N-terminal MPQL motif located in the intermembrane space (IMS) (Stephens et al., 2000), followed by a trans-membrane helix, terminated in yeasts by the conserved Pro33, and a short hydrophilic C-terminal stretch. This sequence fits the density well, with Pro33 at the kink. The trans-membrane helix of subunit *8* has a short connecting density in the IMS toward the *c*-ring and below the first helix hairpin of the *a* subunit, which accommodates the conserved MPQL motif. Thus, the N terminus of subunit *8* appears to anchor the horizontal four-helix bundle of subunit *a* in its position within the F_o assembly.

The longer mammalian subunit *8* has been shown to interact at its C terminus with the peripheral stalk subunits *b* and *d* (Lee et al., 2015); in plant mitochondria, subunit *8* is as long as a typical *b* subunit. This subunit was thought to have no prokaryotic equivalent (Lee et al., 2015; Stephens et al., 2003), but comparison with the *b* subunit of α-proteobacteria, which share a common ancestor with mitochondria, strikingly reveals the same N-terminal MPQL motif. Therefore, the mitochondrial subunit *8* derives from one of the two *b* subunits of its bacterial

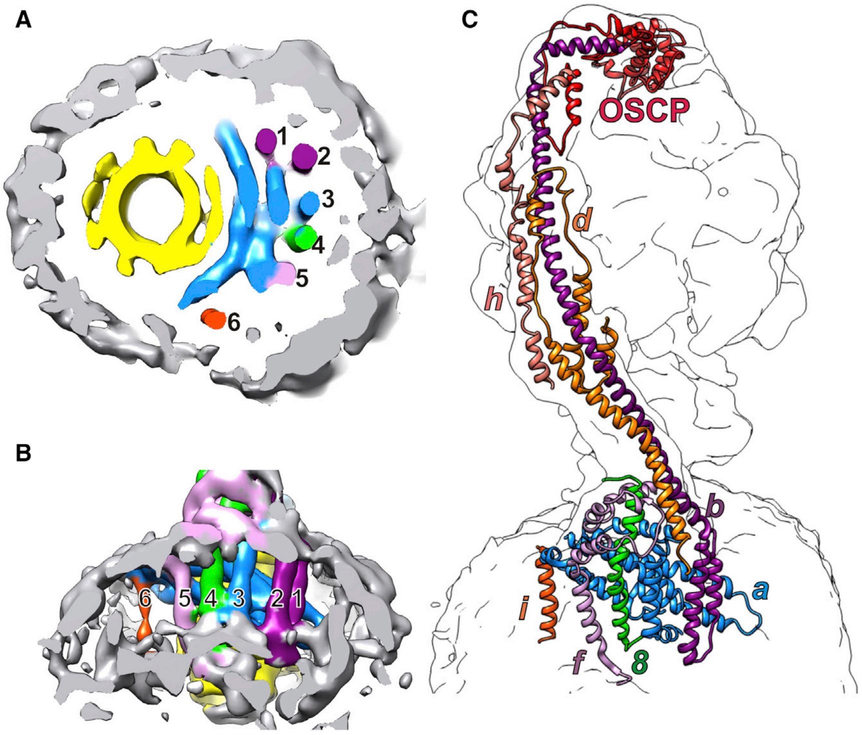

Figure 3. Assignment of the *Yarrowia lipolytica* F_1F_o-ATP Synthase Stator Region

(A and B) Cross sections through the F_o stator region as indicated in Figure 2A. (A) View from the matrix and (B) from the membrane. Four horizontal and six vertical helix densities (labeled 1–6) next to the c_{10} ring rotor (yellow) in the detergent micelle (gray) were assigned to stator subunits. Blue, subunit *a*; purple, subunit *b*; green, subunit *8*; lavender, subunit *f*; dark orange, subunit *i*.

(C) Overview of peripheral stalk and stator subunits in the cryo-EM map. The overall map resolution of 7.8 Å in Figure 2A improved upon masking to 6.2 Å for the F_o dimer (A and B) and 6.9 Å for the F_1 complex (C).

ancestor and is truncated in the mammalian and fungal lines. Subunit *8* is one of the few mitochondrially encoded ATP synthase components in *Y. lipolytica*, together with the *a* and *c* subunits (Kerscher et al., 2001), consistent with its bacterial origin.

Subunit f

Helix 5 (lavender in Figure 3) is the most likely candidate for the nuclear-encoded trans-membrane subunit *f*. In yeast, this 100-residue subunit has a hydrophilic N-terminal domain on the matrix side and a predicted C-terminal trans-membrane helix (Figure S4C). Three curved densities at the base of the peripheral stalk (Figure 3B) that surround the matrix extension of subunit *8* are assigned to the N terminus of subunit *f*. The sharp changes in direction between the densities assigned to this subunit are consistent with the positions of conserved prolines in the *f* subunit sequence alignment.

Subunit i

Finally, the density of helix 6 (orange in Figures 3A and 3B) is weaker than the others. Based on its position next to the *a* subunit, we assign it to the yeast-specific, non-essential subunit *i*, which is present in both the monomer and the dimer in *Y. lipolytica* (Table S1) and has been shown to interact with subunits *a*, *f*, *d*, and *g* (Paumard et al., 2000).

Our assignments are fully consistent with all previously reported chemical crosslinking results of ATP synthases from yeasts, metazoans, and bacteria (DeLeon-Rangel et al., 2013; Jiang and Fillingame, 1998; Schwem and Fillingame, 2006; Stephens et al., 2003) (Figure S6).

Subunits e and g at the Dimer Interface

We collected a cryo-EM dataset of 1*YL*F_1F_o and generated a 3D map of the monomeric *Y. lipolytica* ATP synthase at 8.4 Å resolution (Figure 5). Unlike the dimer, 1*YL*F_1F_o does not contain the dimer-specific subunits *e*, *g*, and *k* (Table S1). The bovine monomer has subunits *e* and *g*, but not *k* (Baker et al., 2012). A comparison of the 3D map volumes therefore reveals the location of *e* and *g* in the dimer map (Figures 5D and 5F). They occupy a roughly triangular density on the dimer interface next to the N-terminal trans-membrane helices of subunit *b*, with a narrow extension that protrudes ~40 Å into the IMS. This density is similar to the *e*/*g* density assigned in the bovine monomer (Zhou et al., 2015), but the orientation of the IMS extension is different (see below).

Subunit *e* is predicted to have an N-terminal trans-membrane helix with a conserved, essential GxxxG motif, a signature of helix-helix interaction (Arselin et al., 2003), and a hydrophilic C terminus that would account for the IMS extension. The *g* subunit can be crosslinked to the N terminus of *b* in the matrix (Soubannier et al., 1999). Deleting the first trans-membrane helix of *b* results in the loss of *g* and dissociation of the dimer (Soubannier et al., 2002), indicating that *g* contributes to dimer stability.

Subunit *g* consists of an N-terminal matrix domain and a predicted C-terminal trans-membrane helix that likewise contains a conserved GxxxG motif. Subunits *e* and *g* may thus form a tight heterodimer in the membrane via their GxxxG motifs. The helices in such a tight heterodimer would not be resolved at 6.2 Å, like the inner helices of the *c*-ring, which are known to interact through such motifs (Vonck et al., 2002). There is no contact between the *e*/*g* density of one monomer to any subunit of the other, so *e* and *g* do not participate directly in dimer formation. Side views of the bovine and *Y. lipolytica* maps (Figure S7A) indicate that each *e*/*g* heterodimer bends the membrane by ~50°, resulting in the ~100° kink observed in the dimer. The most prominent direct dimer contact is formed by the C-terminal domain of subunit *f* (Figure 6). The C terminus of subunit *f* contains conserved charged and polar residues that would mediate this interaction (Figure S3C). The membrane curvature induced by subunits *e* and *g* appears to be necessary to position the C-terminal domains of the *f* subunits in both monomers for interaction across the interface, resulting in dimer formation.

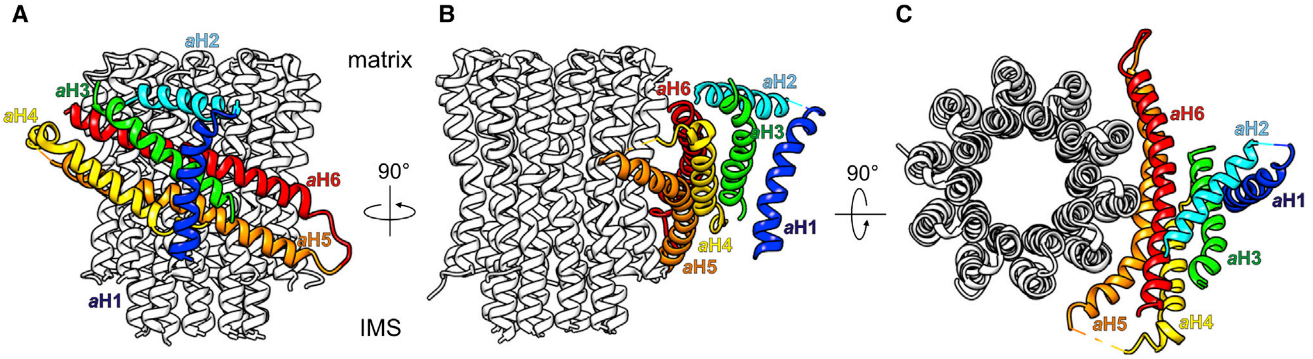

Figure 4. Subunit *a*

View (A) from the dimer interface, (B) along the *a*/*c* interface, and (C) from the matrix. The hydrophilic N terminus on the IMS side (region (i) in the text) is not resolved. Helix *a*H1 (blue, region (ii)) is the only vertical trans-membrane helix of subunit *a*. The amphipathic helix *a*H2 (cyan, region (iii)) runs along the matrix membrane surface. The membrane-intrinsic helices *a*H3 (green) and *a*H4 (yellow) of region (iv) form a hairpin. Regions (v) and (vi) are the unresolved connection between helices *a*H4 and *a*H5. The membrane-intrinsic helices *a*H5 (orange) and *a*H6 (red) form a second, longer hairpin (region (vii)), tilted by 20°–25° relative to the membrane plane. *a*H5 follows the curve of the *c*-ring (light gray) closely. *a*H5 and *a*H6 are ~70 Å long and in contact with 3–4 *c* subunits, while the helices in the distal hairpin, *a*H3 and *a*H4, are ~45 and ~35 Å long; neither is in direct contact with the *c*-ring. The *a*H4/*a*H5 hairpin loop is on the IMS side, while the C terminus is exposed on the matrix surface. All six *a* subunit helices are highly conserved (Figure S5).

DISCUSSION

Rotational F_1 States

A detailed comparison of the *YL*F_1c_{10} crystal structure to the bovine (Abrahams et al., 1994) and *Saccharomyces cerevisiae* (Kabaleeswaran et al., 2006) F_1 and F_1c_{10} complexes (Figure S8) reveals that the three conformational states of the corresponding α/β heterodimers are very similar in the two yeast species, with root-mean-square deviation (RMSD) values below 1.7 Å, while nucleotide binding and C-terminal regions of the bovine β subunits differ (Table S2; Movies S4 and S5). In *YL*F_1c_{10} both the β_E and the β_{DP} site are more open than in the bovine complex, while their β_{TP} sites are similar. Overall, the three β subunits resemble one another more closely in *Y. lipolytica* than in the bovine and *S. cerevisiae* complexes (Figure S8B).

Aligning the γ subunits in all F_1 X-ray structures and comparing the relative positions of the conserved P loop in the β_{DP} subunit (Figure S8A), we find that the *YL*F_1c_{10} P loop is shifted to a position that, in bovine F_1 (Rees et al., 2012), would indicate a post-hydrolysis or pre-product release state. The post-hydrolysis position of the γ subunit in *YL*F_1c_{10} agrees with the presence of bound Mg·ADP in the catalytic β_{DP} and β_{TP} sites. Since *YL*F_1c_{10} was crystallized without added nucleotides, the ADP originates from ATP hydrolysis during isolation or crystallization (Abrahams et al., 1996; Gledhill et al., 2007). The fact that ADP is present in the *Y. lipolytica* β_{DP} at this late stage of hydrolysis without addition of nucleotide-stabilizing azide (Bowler et al., 2006) is surprising, as ADP was not found in the binding sites of other F_1 complexes crystallized under similar conditions (Bianchet et al., 1998; Stocker et al., 2007). This might indicate a higher nucleotide affinity of the *Y. lipolytica* β_{DP} site. In contrast, phosphate (P_i) was not detected in the β_{DP} and β_{TP} sites, in line with a possible alternative leaving route for P_i, as described for *S. cerevisiae* F_1 (Kabaleeswaran et al., 2006).

We found the inhibitor protein *IF1* bound to one of the three different rotational F_1 states in the cryo-EM maps, but not in the *YL*F_1c_{10} X-ray structure, indicating that it was lost during crystallization. By contrast, *IF1* was present in all seven rotary states in the cryo-EM maps of monomeric bovine F_1F_o-ATP synthase (Zhou et al., 2015), as would be expected since the complex was purified by *IF1* affinity chromatography. Apart from the absence of *IF1* in the β_{DP} region (Figure 2D), the 3.5 Å *YL*F_1c_{10} crystal structure matches the dimer map closely (Figures 2A, S3H, and S3I). Like the X-ray structure, the cryo-EM map therefore shows a post-hydrolysis state.

Structure of the F_o Stator

The subunit *a* structure is remarkably conserved in F_1F_o-ATP synthases. Densities for all six helices of our *Y. lipolytica* structure are also present in the same orientations in the cryo-EM map of the bovine heart monomer (Zhou et al., 2015), while four helices are present in the recent 4 Å X-ray structure of the bacterial complex (Morales-Rios et al., 2015) (Figure S7B). The *Polytomella* cryo-EM map (Allegretti et al., 2015) has elements that correspond to each of the six *a* subunit helices in *Y. lipolytica*, even though the polypeptide sequences diverge. The other *Polytomella* stator subunits do not resemble those of yeasts, metazoans, or bacteria.

The cryo-EM map of the bovine monomer shows four trans-membrane helices, two of which were identified as belonging to the peripheral stalk subunit *b*, and one each to subunits *a* and *A6L* (subunit *8* in fungi) (Zhou et al., 2015). All helices superpose well on our map and the assignment agrees with ours, except that there is no density for the *f* subunit in the bovine map (Zhou et al., 2015). Although the bovine complex prepared according to Runswick et al. (Runswick et al., 2013) should contain the *f* subunit, this subunit was not identified by Zhou et al. Subunit *f* may have dissociated during isolation of the bovine monomer, suggesting that it is not firmly attached. As the *f* subunit is responsible for direct dimer contacts in our assignment (Figure 6), its dissociation from the bovine complex

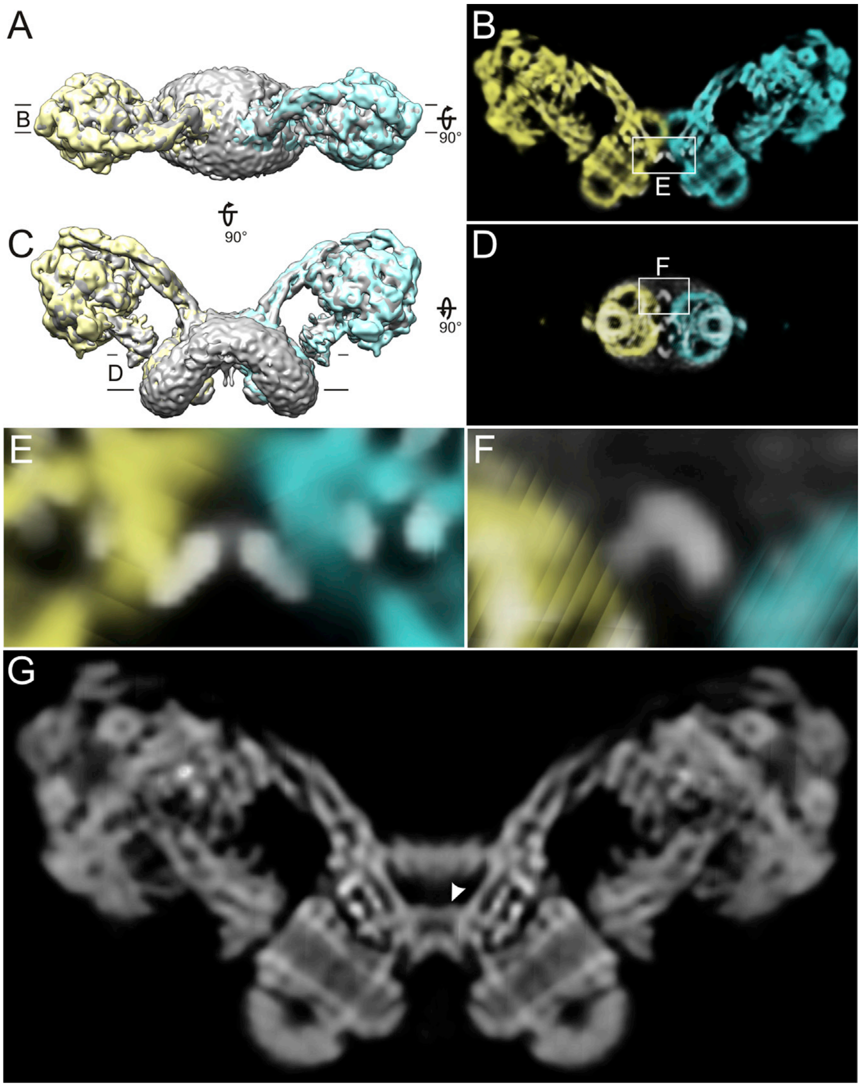

Figure 5. Dimer Interface

(A) Matrix view of the *Y. lipolytica* ATP synthase cryo-EM dimer map (gray) with superposed monomer maps (yellow and blue).

(B) Vertical slice through monomer maps as indicated in (A). Dimer contacts are mediated by protein densities outside the detergent micelle of the monomer (boxed).

(C) Side view of superposed maps in (A).

(D) Horizontal slice through the dimer interface as indicated in (C). The white density (boxed) belongs to membrane subunits present in the dimer, but not in the monomer. LC-MS analysis of monomer and dimer subunit composition identifies these subunits as *e* and *g* (Table S1).

(E) Detailed view of the lumenal dimer contacts as indicated in (B).

(F) Detailed view of the density assigned to the dimer-specific subunits *e* and *g* as indicated in (D).

(G) Central slice of the dimer. A sheet-like density (arrowhead) connecting subunits *a* and *8* may be the yeast-specific subunit *k*, the only unassigned protein in the dimer map.

may explain why dimers from mammalian mitochondria are, in our experience, less stable.

The X-ray structure of the bacterial F_1F_o complex from *Paracoccus denitrificans* (Morales-Rios et al., 2015) shows only two of the trans-membrane helices in the F_o stator, which superpose well on the trans-membrane helix of subunit *8* and *a*H1 in the *Y. lipolytica* dimer (panel (iv) in Figure S7B). Therefore, these helix densities, which were unassigned in the *Paracoccus* map, belong to one of the two bacterial *b* subunits and the trans-membrane helix of subunit *a* (*a*H1), lending strong support to our conclusion that mitochondrial subunit *8* derives from a bacterial *b* subunit. Surprisingly, the trans-membrane helix of the second *b* subunit seems to be completely absent in the *Paracoccus* structure, indicating that it is flexible or disordered in the 4 Å X-ray map.

Proton Translocation through F_o

Ion translocation through F_o is mediated by the *a* subunit and the *c*-ring (Figure 7). A number of high-resolution X-ray structures of ATPase *c*- or *K*-rings have shown that the conserved glutamate residues in the *c* subunits lock the protons (or Na^+) in the hydrophobic environment of the lipid bilayer (Meier et al., 2005; Murata et al., 2005; Pogoryelov et al., 2009) but open to release the ions in a hydrophilic environment (Mizutani et al., 2011; Pogoryelov et al., 2010; Symersky et al., 2012). The recent cryo-EM structures of the *Polytomella* ATP synthase and *S. cerevisiae* V-type ATPase indicate two aqueous half-channels at the subunit *a*/*c* interface that are thought to conduct protons to and from the *c*-ring protonation sites (Allegretti et al., 2015). We find similar aqueous half-channels in equivalent positions of the *Y. lipolytica* dimer map (Figures 7A and 7B). Conserved hydrophilic residues of *a*H5 and *a*H6 line the aqueous cavity on the matrix side (Figures 7C and 7D). The conserved charged and polar residues of *a*H5, starting with Glu168 four helix turns upstream of Arg182, create the hydrophilic environment to release the proton from the opposing *c* subunit glutamate into the matrix. The $\sim$20° tilt of the *a*H5/*a*H6 hairpin places the hairpin loop close to the IMS surface, and the C terminus of *a*H6 on the matrix side (Figure 7A). Consequently, the lumenal half-channel near the hairpin loop and the matrix half-channel at the C terminus of subunit *a* are laterally offset, as anticipated (Junge et al., 1997; Vik and Antonio, 1994). The proton entrance channel on the IMS side is likely to include the conserved Asn186 in *a*H5, Asn106 in *a*H3, and the exchangeable pair His191/Glu229 in *a*H5 and *a*H6, one of which is Glu or Asp in all ATP synthases (Figure S5). Rather than mediating proton release, the essential Arg182 would allow only deprotonated *c* subunit glutamate side chains to pass (Figure 7D). Removal of this arginine by mutagenesis uncouples ion

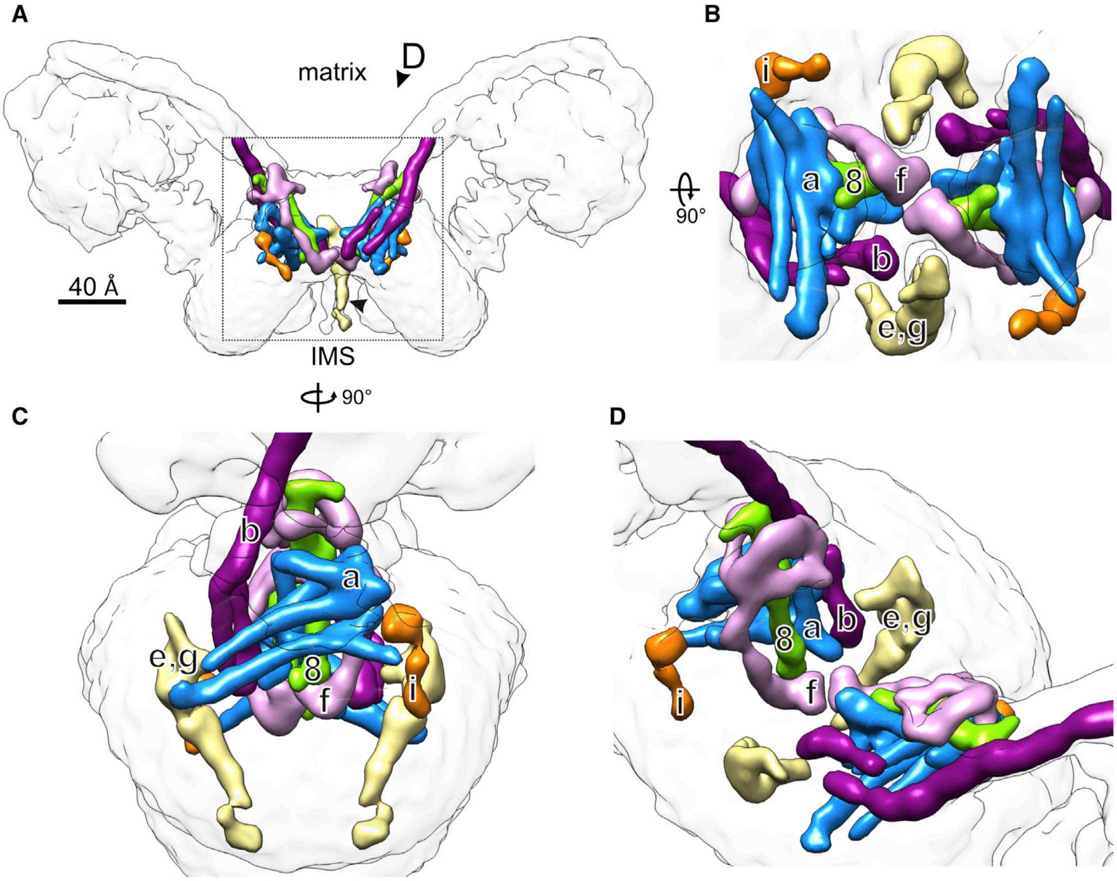

Figure 6. The F_o Stator

Membrane protein densities in the two F_o stator complexes of the *Y. lipolytica* ATP synthase dimer. Subunits *a*, *b*, *f*, *i*, and *8* are colored as in Figure 2. Subunits *e* and *g* are ivory.

(A) Side view of the dimer interface. The IMS extensions of subunit *e* (arrowhead) were segmented at a lower contour level. F_o subunits of the two ATP synthase monomers interact on the IMS side. A 40 Å gap on the matrix side contains lipid or detergent.

(B) View from the IMS. The C-terminal segment of subunit *f* mediates a direct protein contact between the two monomers in the dimer. Densities on either side of the protein contact are assigned to subunits *e* and *g* (see Figure 6).

(C) View from the *c*-ring.

(D) Oblique view in the direction indicated in (A). For an evaluation of Cys-Cys crosslinks, see Figure S6 and Table S3.

translocation from ATP synthesis (Mitome et al., 2010), as it results in futile proton translocation without *c*-ring rotation.

Clinical studies show that mutations in *a*H5 and *a*H6 impair the functionality or assembly of ATP synthase in human mitochondria (Kucharczyk et al., 2009; Xu et al., 2015), giving rise to severe neuropathological disorders (Houstek et al., 2006), such as the maternally inherited Leigh syndrome or retinitis pigmentosa (Kucharczyk et al., 2009). A molecular understanding of the exact ion translocation mechanism is essential for exploring future therapy. The structure of the genetically accessible *Y. lipolytica* ATP synthase now provides a basis for structural and functional studies to combat these diseases at the molecular level.

Dimer Contacts

In the *Y. lipolytica* F_1F_o dimer, which contains subunits *e* and *g*, the detergent belt is bent by roughly 100°. In the bovine F_1F_o monomer, which contains subunits *e* and *g*, it is bent by ~50° (Baker et al., 2012; Zhou et al., 2015), but in the *Y. lipolytica* monomer, which lacks *e* and *g*, the belt is more or less straight (Figure S7A; Table S1). We conclude that *e* and *g* are chiefly responsible for inducing the membrane curvature that results in mitochondrial cristae morphology.

There is no evident function for the C-terminal IMS helix of subunit *e* in the dimeric complex (Figure 6A). In contrast to the bovine map (Zhou et al., 2015), this elongated density protrudes straight out of the F_o stator region into the IMS, while in the bovine map it is curved and contacts the central plug of the *c*-ring. A role in the formation of higher-order ATP synthase oligomers and dimer rows (Strauss et al., 2008; Davies et al., 2012) seems unlikely, as the extension is not easily accessible and appears to point in the wrong direction for interaction between adjacent dimers (Figure 5D). Moreover, the dimer spacing along the rows is irregular (Daum et al., 2013), which suggests that the inter-dimer protein contacts are dynamic. Instead, the subunit *e* extension with its predicted coiled-coil propensity may play a role in complex assembly.

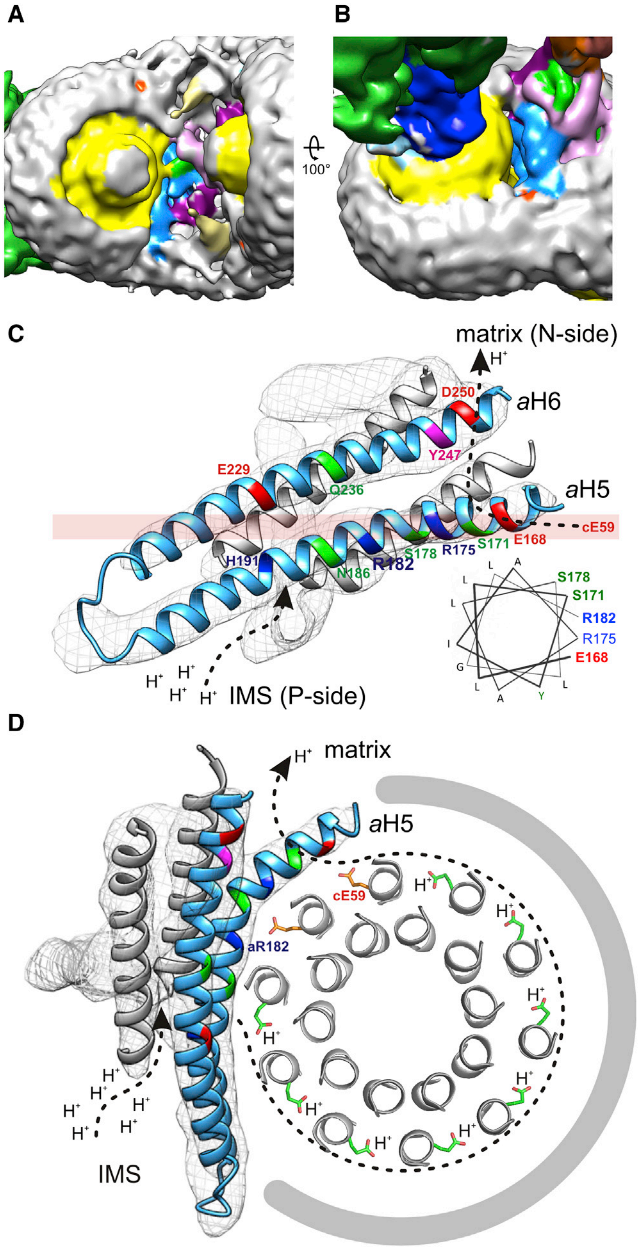

Figure 7. Ion Translocation through F_o

(A) IMS surface of the *Y. lipolytica* dimer. Subunits are color coded as in Figure 2. The *c*-ring and the dimer contact region are solvent exposed. *a*H5 of subunit *a* (light blue) is visible from the IMS side. The central plug on the *c*-rings consists of lipid or detergent (Meier et al., 2001).

(B) Oblique view from the matrix shows the gap between the *c*-ring (yellow) and subunit *a* (light blue).

(C) Fitted *a*H5 and *a*H6 hairpin of subunit *a* next to the *c*-ring rotor with modeled positions of conserved positive (blue), negative (red), and polar residues (green). The helical wheel projection (inset) indicates alternating polar, charged, and hydrophobic residues at the start of *a*H5, with polar or charged residues oriented toward the *c*-ring. The transparent pink bar indicates the level at which the protonated *c*-ring glutamates rotate past the strictly conserved *a*Arg182.

(D) Section through the c_{10}-ring (gray) and subunit *a* (gray mesh) with a cartoon model (blue) of the *c*-ring at the level of protonated glutamates in the locked (green) or open (orange) conformation (Pogoryelov et al., 2010; Symersky et al., 2012). *a*H6 peels away from the *c*-ring, accounting for the observed gap at the *a/c* interface. Dashed arrows in (C) and (D) indicate the proton pathway from the IMS (P side) to the matrix (N side), as proposed for the *Polytomella* ATP synthase (Allegretti et al., 2015).

The two F_o subcomplexes in the membrane are separated by a wedge-shaped gap that is ~40 Å wide on the matrix side and narrows to ~15 Å on the IMS side (Figures 5G and 6A). On the matrix side, this wedge appears to be filled by lipid or detergent, as there is no distinct protein density. The tip of the wedge on the IMS side contains the dimer contact domain assigned to the C terminus of subunit *f* (Figure 6). The center of the wedge-shaped gap is bridged by a conspicuous sheet of density connecting the trans-membrane helices of subunits *a* and *8* (Figure 5G), which may contribute to dimer formation. It is tempting to speculate that this density belongs to the so far unassigned yeast-specific subunit *k*, a small, partly hydrophilic protein without predicted trans-membrane helix (Figure S4D). The sheet may form a barrier between the membrane leaflet on the matrix side and hydrophilic protein domains on the IMS side of the dimer interface. The matrix half of the wedge-shaped space has the thickness of one membrane leaflet, implying a new and unusual membrane architecture in this region of the dimer.

Role of ATP Synthase Dimers in Membrane Morphology

The comparatively simple bacterial and chloroplast ATP synthases consist of eight or nine different subunits, which are sufficient for ATP production. The chloroplast ATP synthase has been shown to be monomeric (Daum et al., 2010), and no ATP synthase dimers have been reported in bacteria. By contrast, all known mitochondrial ATP synthases form dimers in the membrane that self-assemble into rows (Davies et al., 2012). Mitochondrial ATP synthases of yeasts and metazoans have eight additional subunits of so far unexplained structure and function. Our map shows how the mitochondria-specific subunits in the mitochondrial F_o subcomplex are arranged, and that most of them have a role in dimer formation: the N-terminal trans-membrane extension of *b* anchors *e* and *g* to the complex, and the *e/g* heterodimer induces local membrane curvature, which in turn appears to enable the IMS domain of *f* (and possibly subunit *k*) to establish protein-protein contacts across the dimer interface.

ATP synthase dimer rows are a prerequisite for the formation of inner membrane cristae (Davies et al., 2012), a hallmark signature of mitochondrial morphology. Cristae formation extends the membrane surface to accommodate a large number of respiratory chain complexes, making it possible to meet the high energy demands of eukaryotic cells (Lane and Martin, 2010). They also form a mitochondrial sub-compartment that supports a locally increased proton concentration in the confined cristae space. The structure of the mitochondrial ATP synthase dimer thus offers new insights into how mitochondria became the efficient power plants of eukaryotic cells.

EXPERIMENTAL PROCEDURES

ATP synthase dimers fully competent for oligomycin-sensitive ATP hydrolysis were isolated from mitochondria prepared from large-scale *Yarrowia lipolytica*

cultures (Kashani-Poor et al., 2001) and purified by glycerol gradient centrifugation, anion exchange, and gel filtration chromatography. Cryo-EM grids of 2*YL*F_1F_o dimers and 1*YL*F_1F_o monomers were prepared and images were recorded on an in-column energy-filtered JEOL 3200 FSC electron microscope with a Gatan K2 direct electron detector in movie mode. Global beam-induced motion was corrected by movie frame processing (Li et al., 2013). Two- and three-dimensional classification and 3D map refinement were carried out with RELION 1.3 (Scheres, 2012). Crystals of the F_1c_{10} subcomplex were grown from concentrated samples of the 1*YL*F_1F_o. X-ray data were collected at beamline PX-II X10SA (Swiss Light Source), and the structure was determined by molecular replacement with a model based on the *S. cerevisiae* F_1c_{10} complex (PDB: 2XOK) (Stock et al., 1999).

ACCESSION NUMBERS

The structure coordinates have been deposited in the Protein Data Bank under ID code PDB: 5FL7. The EM data were deposited in the worldwide PDB under ID codes wwPDB: EMD-8151 (symmetric dimer map); EMD-8152 (masked F_o map); and EMD-8153, EMD-8154, and EMD-8155 (subclass 1, 2, and 3 of the masked F_1F_o monomer).

SUPPLEMENTAL INFORMATION

Supplemental Information includes Supplemental Experimental Procedures, eight figures, three tables, and five movies and can be found with this article online at http://dx.doi.org/10.1016/j.molcel.2016.05.037.

AUTHOR CONTRIBUTIONS

T.M. initiated the study; T.M. and W.K. directed the project; V.Z. provided mitochondrial membranes; A.H. purified the protein; D.J.M. devised the cryo-EM data collection procedure; A.H. and D.J.M. collected cryo-EM data; A.H. and J.V. analyzed cryo-EM data; K.P. grew crystals and collected X-ray data; K.P., M.B., and T.M. analyzed X-ray data; T.M., J.V., W.K., K.P., M.B., and A.H. wrote the paper.

ACKNOWLEDGMENTS

We thank Karin Siegmund for technical assistance, Özkan Yildiz and Juan F. Castillo-Hernández for computer support, and the PXII beamline staff at the Swiss Light Source for continuous support. This work was funded by the Max-Planck Society, the Collaborative Research Center (SFB) 807 of the German Research Foundation (DFG), the Cluster of Excellence Frankfurt “Macromolecular Complexes” (DFG Project EXC 115), and by the Wellcome Trust [WT110068/Z/15/Z].

Received: December 29, 2015
Revised: April 21, 2016
Accepted: May 26, 2016
Published: June 30, 2016

REFERENCES

Abrahams, J.P., Leslie, A.G.W., Lutter, R., and Walker, J.E. (1994). Structure at 2.8 Å resolution of F_1-ATPase from bovine heart mitochondria. Nature *370*, 621–628.

Abrahams, J.P., Buchanan, S.K., Van Raaij, M.J., Fearnley, I.M., Leslie, A.G.W., and Walker, J.E. (1996). The structure of bovine F_1-ATPase complexed with the peptide antibiotic efrapeptin. Proc. Natl. Acad. Sci. USA *93*, 9420–9424.

Allegretti, M., Klusch, N., Mills, D.J., Vonck, J., Kühlbrandt, W., and Davies, K.M. (2015). Horizontal membrane-intrinsic α-helices in the stator a subunit of an F-type ATP synthase. Nature *521*, 237–240.

Arnold, I., Pfeiffer, K., Neupert, W., Stuart, R.A., and Schägger, H. (1998). Yeast mitochondrial F_1F_0-ATP synthase exists as a dimer: identification of three dimer-specific subunits. EMBO J. *17*, 7170–7178.

Arselin, G., Giraud, M.F., Dautant, A., Vaillier, J., Brèthes, D., Coulary-Salin, B., Schaeffer, J., and Velours, J. (2003). The GxxxG motif of the transmembrane domain of subunit e is involved in the dimerization/oligomerization of the yeast ATP synthase complex in the mitochondrial membrane. Eur. J. Biochem. *270*, 1875–1884.

Baker, L.A., Watt, I.N., Runswick, M.J., Walker, J.E., and Rubinstein, J.L. (2012). Arrangement of subunits in intact mammalian mitochondrial ATP synthase determined by cryo-EM. Proc. Natl. Acad. Sci. USA *109*, 11675–11680.

Bianchet, M.A., Hullihen, J., Pedersen, P.L., and Amzel, L.M. (1998). The 2.8-Å structure of rat liver F_1-ATPase: configuration of a critical intermediate in ATP synthesis/hydrolysis. Proc. Natl. Acad. Sci. USA *95*, 11065–11070.

Bowler, M.W., Montgomery, M.G., Leslie, A.G.W., and Walker, J.E. (2006). How azide inhibits ATP hydrolysis by the F-ATPases. Proc. Natl. Acad. Sci. USA *103*, 8646–8649.

Boyer, P.D. (1997). The ATP synthase—a splendid molecular machine. Annu. Rev. Biochem. *66*, 717–749.

Cain, B.D. (2000). Mutagenic analysis of the F_0 stator subunits. J. Bioenerg. Biomembr. *32*, 365–371.

Daum, B., Nicastro, D., Austin, J., 2nd, McIntosh, J.R., and Kühlbrandt, W. (2010). Arrangement of photosystem II and ATP synthase in chloroplast membranes of spinach and pea. Plant Cell *22*, 1299–1312.

Daum, B., Walter, A., Horst, A., Osiewacz, H.D., and Kühlbrandt, W. (2013). Age-dependent dissociation of ATP synthase dimers and loss of inner-membrane cristae in mitochondria. Proc. Natl. Acad. Sci. USA *110*, 15301–15306.

Davies, K.M., Strauss, M., Daum, B., Kief, J.H., Osiewacz, H.D., Rycovska, A., Zickermann, V., and Kühlbrandt, W. (2011). Macromolecular organization of ATP synthase and complex I in whole mitochondria. Proc. Natl. Acad. Sci. USA *108*, 14121–14126.

Davies, K.M., Anselmi, C., Wittig, I., Faraldo-Gómez, J.D., and Kühlbrandt, W. (2012). Structure of the yeast F_1F_o-ATP synthase dimer and its role in shaping the mitochondrial cristae. Proc. Natl. Acad. Sci. USA *109*, 13602–13607.

DeLeon-Rangel, J., Ishmukhametov, R.R., Jiang, W., Fillingame, R.H., and Vik, S.B. (2013). Interactions between subunits *a* and *b* in the rotary ATP synthase as determined by cross-linking. FEBS Lett. *587*, 892–897.

Dickson, V.K., Silvester, J.A., Fearnley, I.M., Leslie, A.G.W., and Walker, J.E. (2006). On the structure of the stator of the mitochondrial ATP synthase. EMBO J. *25*, 2911–2918.

Emsley, P., Lohkamp, B., Scott, W.G., and Cowtan, K. (2010). Features and development of Coot. Acta Crystallogr. D Biol. Crystallogr. *66*, 486–501.

Eya, S., Maeda, M., and Futai, M. (1991). Role of the carboxyl terminal region of H^+-ATPase (F_0F_1) a subunit from *Escherichia coli*. Arch. Biochem. Biophys. *284*, 71–77.

Fujikawa, M., Sugawara, K., Tanabe, T., and Yoshida, M. (2015). Assembly of human mitochondrial ATP synthase through two separate intermediates, F_1-*c*-ring and *b-e-g* complex. FEBS Lett. *589* (19 Pt B), 2707–2712.

Giraud, M.F., Paumard, P., Sanchez, C., Brèthes, D., Velours, J., and Dautant, A. (2012). Rotor architecture in the yeast and bovine F_1-c-ring complexes of F-ATP synthase. J. Struct. Biol. *177*, 490–497.

Gledhill, J.R., Montgomery, M.G., Leslie, A.G.W., and Walker, J.E. (2007). How the regulatory protein, IF1, inhibits F_1-ATPase from bovine mitochondria. Proc. Natl. Acad. Sci. USA *104*, 15671–15676.

Houstek, J., Pícková, A., Vojtísková, A., Mrácek, T., Pecina, P., and Jesina, P. (2006). Mitochondrial diseases and genetic defects of ATP synthase. Biochim. Biophys. Acta *1757*, 1400–1405.

Jiang, W., and Fillingame, R.H. (1998). Interacting helical faces of subunits a and c in the F_1F_0 ATP synthase of *Escherichia coli* defined by disulfide cross-linking. Proc. Natl. Acad. Sci. USA *95*, 6607–6612.

Junge, W., Lill, H., and Engelbrecht, S. (1997). ATP synthase: an electrochemical transducer with rotatory mechanics. Trends Biochem. Sci. *22*, 420–423.

Kabaleeswaran, V., Puri, N., Walker, J.E., Leslie, A.G.W., and Mueller, D.M. (2006). Novel features of the rotary catalytic mechanism revealed in the structure of yeast F_1 ATPase. EMBO J. *25*, 5433–5442.

Kashani-Poor, N., Kerscher, S., Zickermann, V., and Brandt, U. (2001). Efficient large scale purification of his-tagged proton translocating NADH:ubiquinone oxidoreductase (complex I) from the strictly aerobic yeast *Yarrowia lipolytica*. Biochim. Biophys. Acta *1504*, 363–370.

Kerscher, S., Durstewitz, G., Casaregola, S., Gaillardin, C., and Brandt, U. (2001). The complete mitochondrial genome of *Yarrowia lipolytica*. Comp. Funct. Genomics *2*, 80–90.

Kucharczyk, R., Zick, M., Bietenhader, M., Rak, M., Couplan, E., Blondel, M., Caubet, S.D., and di Rago, J.P. (2009). Mitochondrial ATP synthase disorders: molecular mechanisms and the quest for curative therapeutic approaches. Biochim. Biophys. Acta *1793*, 186–199.

Kühlbrandt, W., and Davies, K.M. (2016). Rotary ATPases: A new twist to an ancient machine. Trends Biochem. Sci. *41*, 106–116.

Lane, N., and Martin, W. (2010). The energetics of genome complexity. Nature *467*, 929–934.

Lee, J., Ding, S., Walpole, T.B., Holding, A.N., Montgomery, M.G., Fearnley, I.M., and Walker, J.E. (2015). Organization of subunits in the membrane domain of the bovine F-ATPase revealed by covalent cross-linking. J. Biol. Chem. *290*, 13308–13320.

Li, X., Mooney, P., Zheng, S., Booth, C.R., Braunfeld, M.B., Gubbens, S., Agard, D.A., and Cheng, Y. (2013). Electron counting and beam-induced motion correction enable near-atomic-resolution single-particle cryo-EM. Nat. Methods *10*, 584–590.

Lightowlers, R.N., Howitt, S.M., Hatch, L., Gibson, F., and Cox, G.B. (1987). The proton pore in the *Escherichia coli* F_0F_1-ATPase: a requirement for arginine at position 210 of the a subunit. Biochim. Biophys. Acta *894*, 399–406.

Meier, T., Matthey, U., Henzen, F., Dimroth, P., and Müller, D.J. (2001). The central plug in the reconstituted undecameric c cylinder of a bacterial ATP synthase consists of phospholipids. FEBS Lett. *505*, 353–356.

Meier, T., Polzer, P., Diederichs, K., Welte, W., and Dimroth, P. (2005). Structure of the rotor ring of F-type Na^+-ATPase from *Ilyobacter tartaricus*. Science *308*, 659–662.

Meier, T., Faraldo-Gómez, J.D., and Börsch, M. (2011). ATP synthase, a paradigmatic molecular machine. In Molecular Machines in Biology, J. Frank, ed. (Cambridge University Press), pp. 208–238.

Mitome, N., Ono, S., Sato, H., Suzuki, T., Sone, N., and Yoshida, M. (2010). Essential arginine residue of the F_0-a subunit in F_0F_1-ATP synthase has a role to prevent the proton shortcut without c-ring rotation in the F_0 proton channel. Biochem. J. *430*, 171–177.

Mizutani, K., Yamamoto, M., Suzuki, K., Yamato, I., Kakinuma, Y., Shirouzu, M., Walker, J.E., Yokoyama, S., Iwata, S., and Murata, T. (2011). Structure of the rotor ring modified with *N,N′*-dicyclohexylcarbodiimide of the Na^+-transporting vacuolar ATPase. Proc. Natl. Acad. Sci. USA *108*, 13474–13479.

Morales-Rios, E., Montgomery, M.G., Leslie, A.G.W., and Walker, J.E. (2015). Structure of ATP synthase from *Paracoccus denitrificans* determined by X-ray crystallography at 4.0 Å resolution. Proc. Natl. Acad. Sci. USA *112*, 13231–13236.

Murata, T., Yamato, I., Kakinuma, Y., Leslie, A.G.W., and Walker, J.E. (2005). Structure of the rotor of the V-type Na^+-ATPase from *Enterococcus hirae*. Science *308*, 654–659.

Noji, H., Yasuda, R., Yoshida, M., and Kinosita, K., Jr. (1997). Direct observation of the rotation of F_1-ATPase. Nature *386*, 299–302.

Nübel, E., Wittig, I., Kerscher, S., Brandt, U., and Schägger, H. (2009). Two-dimensional native electrophoretic analysis of respiratory supercomplexes from *Yarrowia lipolytica*. Proteomics *9*, 2408–2418.

Pagadala, V., Vistain, L., Symersky, J., and Mueller, D.M. (2011). Characterization of the mitochondrial ATP synthase from yeast *Saccharomyces cerevisae*. J. Bioenerg. Biomembr. *43*, 333–347.

Paumard, P., Vaillier, J., Napias, C., Arselin, G., Brèthes, D., Graves, P.V., and Velours, J. (2000). Environmental study of subunit i, a F_0 component of the yeast ATP synthase. Biochemistry *39*, 4199–4205.

Paumard, P., Vaillier, J., Coulary, B., Schaeffer, J., Soubannier, V., Mueller, D.M., Brèthes, D., di Rago, J.P., and Velours, J. (2002). The ATP synthase is involved in generating mitochondrial cristae morphology. EMBO J. *21*, 221–230.

Pogoryelov, D., Yildiz, O., Faraldo-Gómez, J.D., and Meier, T. (2009). High-resolution structure of the rotor ring of a proton-dependent ATP synthase. Nat. Struct. Mol. Biol. *16*, 1068–1073.

Pogoryelov, D., Krah, A., Langer, J.D., Yildiz, Ö., Faraldo-Gómez, J.D., and Meier, T. (2010). Microscopic rotary mechanism of ion translocation in the F_0 complex of ATP synthases. Nat. Chem. Biol. *6*, 891–899.

Rees, D.M., Leslie, A.G.W., and Walker, J.E. (2009). The structure of the membrane extrinsic region of bovine ATP synthase. Proc. Natl. Acad. Sci. USA *106*, 21597–21601.

Rees, D.M., Montgomery, M.G., Leslie, A.G.W., and Walker, J.E. (2012). Structural evidence of a new catalytic intermediate in the pathway of ATP hydrolysis by F_1-ATPase from bovine heart mitochondria. Proc. Natl. Acad. Sci. USA *109*, 11139–11143.

Runswick, M.J., Bason, J.V., Montgomery, M.G., Robinson, G.C., Fearnley, I.M., and Walker, J.E. (2013). The affinity purification and characterization of ATP synthase complexes from mitochondria. Open Biol. *3*, 120160.

Satre, M., de Jerphanion, M.B., Huet, J., and Vignais, P.V. (1975). ATPase inhibitor from yeast mitochondria. Purification and properties. Biochim. Biophys. Acta *387*, 241–255.

Scheres, S.H. (2012). RELION: implementation of a Bayesian approach to cryo-EM structure determination. J. Struct. Biol. *180*, 519–530.

Schwem, B.E., and Fillingame, R.H. (2006). Cross-linking between helices within subunit *a* of *Escherichia coli* ATP synthase defines the transmembrane packing of a four-helix bundle. J. Biol. Chem. *281*, 37861–37867.

Soubannier, V., Rusconi, F., Vaillier, J., Arselin, G., Chaignepain, S., Graves, P.V., Schmitter, J.M., Zhang, J.L., Mueller, D., and Velours, J. (1999). The second stalk of the yeast ATP synthase complex: identification of subunits showing cross-links with known positions of subunit *4* (subunit *b*). Biochemistry *38*, 15017–15024.

Soubannier, V., Vaillier, J., Paumard, P., Coulary, B., Schaeffer, J., and Velours, J. (2002). In the absence of the first membrane-spanning segment of subunit 4 (b), the yeast ATP synthase is functional but does not dimerize or oligomerize. J. Biol. Chem. *277*, 10739–10745.

Stephens, A.N., Roucou, X., Artika, I.M., Devenish, R.J., and Nagley, P. (2000). Topology and proximity relationships of yeast mitochondrial ATP synthase subunit 8 determined by unique introduced cysteine residues. Eur. J. Biochem. *267*, 6443–6451.

Stephens, A.N., Khan, M.A., Roucou, X., Nagley, P., and Devenish, R.J. (2003). The molecular neighborhood of subunit *8* of yeast mitochondrial F_1F_0-ATP synthase probed by cysteine scanning mutagenesis and chemical modification. J. Biol. Chem. *278*, 17867–17875.

Stock, D., Leslie, A.G.W., and Walker, J.E. (1999). Molecular architecture of the rotary motor in ATP synthase. Science *286*, 1700–1705.

Stocker, A., Keis, S., Vonck, J., Cook, G.M., and Dimroth, P. (2007). The structural basis for unidirectional rotation of thermoalkaliphilic F_1-ATPase. Structure *15*, 904–914.

Strauss, M., Hofhaus, G., Schröder, R.R., and Kühlbrandt, W. (2008). Dimer ribbons of ATP synthase shape the inner mitochondrial membrane. EMBO J. *27*, 1154–1160.

Symersky, J., Pagadala, V., Osowski, D., Krah, A., Meier, T., Faraldo-Gómez, J.D., and Mueller, D.M. (2012). Structure of the c_{10} ring of the yeast mitochondrial ATP synthase in the open conformation. Nat. Struct. Mol. Biol. *19*, 485–491, S1.

Vik, S.B., and Antonio, B.J. (1994). A mechanism of proton translocation by F_1F_0 ATP synthases suggested by double mutants of the a subunit. J. Biol. Chem. *269*, 30364–30369.

Vonck, J., von Nidda, T.K., Meier, T., Matthey, U., Mills, D.J., Kühlbrandt, W., and Dimroth, P. (2002). Molecular architecture of the undecameric rotor of a bacterial Na^+-ATP synthase. J. Mol. Biol. *321*, 307–316.

Xu, T., Pagadala, V., and Mueller, D.M. (2015). Understanding structure, function, and mutations in the mitochondrial ATP synthase. Microb. Cell *2*, 105–125.

Zhao, J., Benlekbir, S., and Rubinstein, J.L. (2015). Electron cryomicroscopy observation of rotational states in a eukaryotic V-ATPase. Nature *521*, 241–245.

Zhou, A., Rohou, A., Schep, D.G., Bason, J.V., Montgomery, M.G., Walker, J.E., Grigorieff, N., and Rubinstein, J.L. (2015). Structure and conformational states of the bovine mitochondrial ATP synthase by cryo-EM. eLife *4*, e10180.

Molecular Cell, Volume *63*

Supplemental Information

Structure of a Complete ATP Synthase Dimer Reveals the Molecular Basis of Inner Mitochondrial Membrane Morphology

Alexander Hahn, Kristian Parey, Maike Bublitz, Deryck J. Mills, Volker Zickermann, Janet Vonck, Werner Kühlbrandt, and Thomas Meier

SUPPLEMENTARY FIGURES

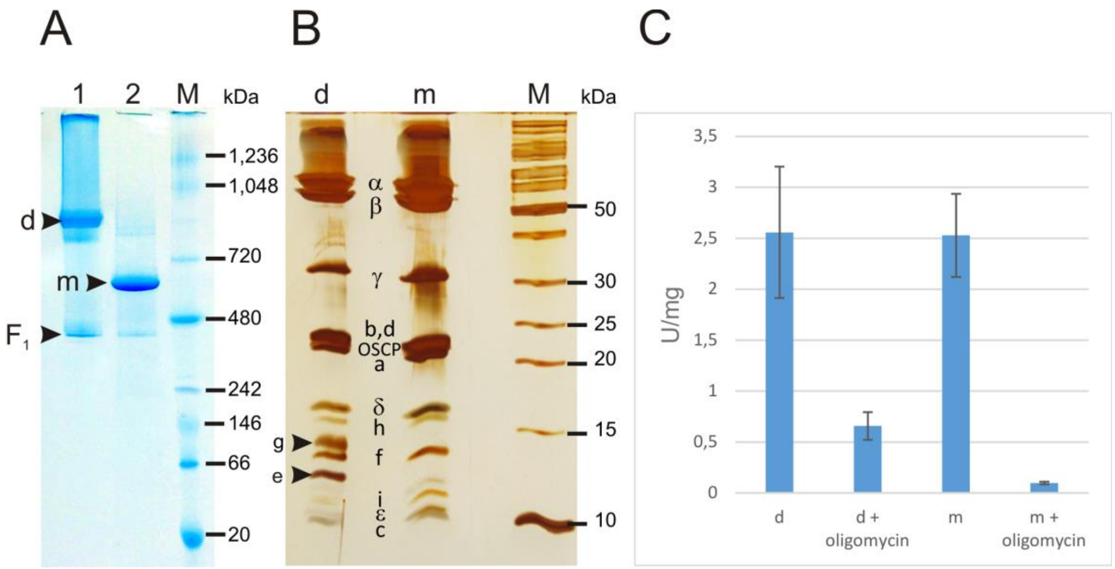

Figure S1, related to Figure 2. Subunit composition of monomeric and dimeric *Y. lipolytica* ATP synthase and ATP hydrolase activities of purified ATPases. The (1) dimeric (digitonin) and (2) monomeric (dodecylmaltoside) purified ATP synthase samples were separated by (**A**) Blue native-PAGE. Indicated protein complexes (d, m) were cut out of the gel and used for LC-MS analysis and 2nd dimension SDS-PAGE. (**B**) ATP synthase subunits were separated in the 2nd dimension SDS-PAGE using a 15% polyacrylamide gel, according to (Schägger and von Jagow, 1987). The subunits are indicated according to their molecular weight (Liu et al., 2015). Molecular weight markers (in kDa) are indicated on the right side of the gels. Arrow heads indicate the presence of subunit *e* and *g* only in the (d) dimer preparation but not in the (m) monomeric ATP synthase preparation. (**C**) Determination of ATP hydrolytic activities using the spectrophotometric coupled assay (Cook et al., 2003) and inhibition of ATP hydrolytic activity upon adding 5 µg/ml oligomycin (= 6.3 µM). Error bars are indicated (*n*=3).

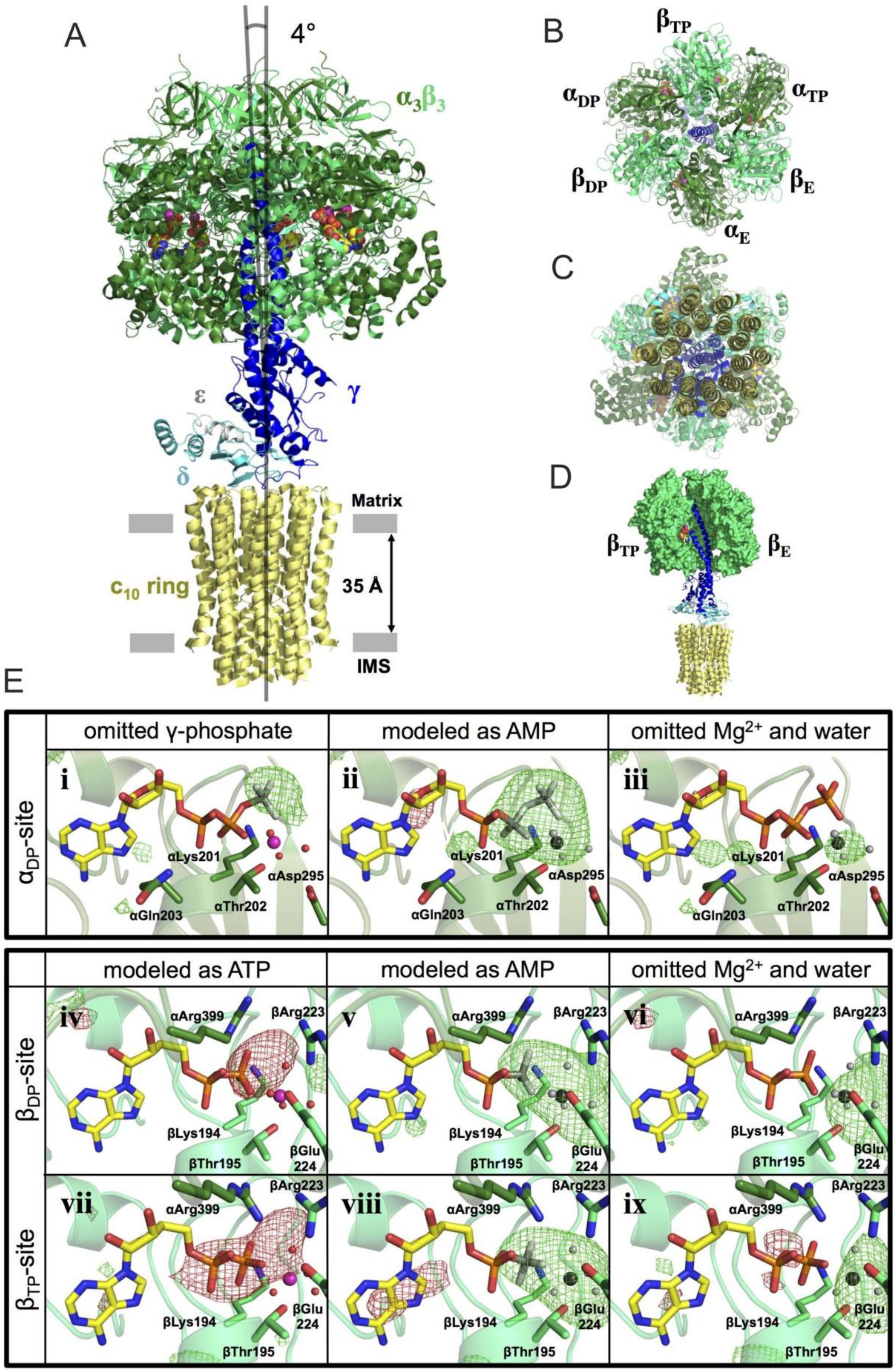
A
4°
α3β3
ε
γ
δ
Matrix
c10 ring
35 Å
IMS
B
βTP
αDP
αTP
βDP
βE
αE
C
D
βTP
βE
E
omitted γ-phosphate
modeled as AMP
omitted Mg2+ and water
αDP-site
i
ii
iii
αLys201
αAsp295
αThr202
αGln203
modeled as ATP
modeled as AMP
omitted Mg2+ and water
βDP-site
iv
v
vi
αArg399
βArg223
βLys194
βThr195
βGlu 224
βTP-site
vii
viii
ix

Figure S2, related to Figure 1. Crystal structure of the *Y. lipolytica* F_1c_{10} ATP synthase subcomplex. Overview from the (**A**) side, (**B**) top and (**C**) bottom. The structure is displayed in ribbon representation. The α- and β-subunits are shown in light and dark green, respectively, the γ-, δ- and ε-subunits are represented in blue, cyan and light grey, respectively, and the *c*-ring is colored in yellow. Nucleotides and Mg^{2+} are drawn as spheres. The symmetry axes of the F_1-stator and F_o-rotor, drawn as grey lines, are tilted by ~4° (as determined by Chimera). The membrane borders are indicated by grey bars. IMS: Intermembrane space. (**D**) Open F_1 (α-subunits omitted) in surface representation (β-subunits) and rotor in ribbon model. (**E**) Difference (omit) maps after modeling different nucleotide ligands at the α_{DP}-, β_{DP}- and β_{TP}-sites. The main chain is represented as a ribbon in dark and light green for the α- and β-subunits, respectively. Coordinating side chains are shown in stick representation. Nucleotides are also in stick representation with carbon, nitrogen, oxygen and phosphorus in yellow, blue, red and orange, respectively; Mg^{2+} and coordinating waters are depicted as spheres in magenta and red, respectively. Omitted atoms are indicated in grey, as a positional reference. The mF_{obs}-DF_{calc} electron density map after refinement is shown as mesh (contoured at ±3.0σ; green, positive; red, negative). *Upper panels*: α_{DP}-site after omitting (i) the γ-phosphate, (ii) the β- and γ-phosphate, Mg^{2+} and coordinating water, or (iii) Mg^{2+} and water. The positive peaks appearing after omitting parts of the ligand confirm the presence of Mg·ATP[·3 H_2O]. *Middle panels*: β_{DP}-site after (iv) refining with Mg·ATP[·3 H_2O] instead of Mg·ADP[·4 H_2O], (v) refining with AMP, or (vi) omitting Mg^{2+} and water. *Lower panels*: β_{TP}-site after (vii) refining with Mg·ATP[·3 H_2O] instead of Mg·ADP[·4 H_2O], (viii) refining with AMP, or (ix) omitting Mg^{2+} and water. In both the β_{DP}- and β_{TP}-site, negative density peaks appearing upon refinement of a triphosphate ligand indicate the absence of a γ-phosphate. In contrast, the presence of both the β-phosphates and the Mg^{2+} ions (including coordinating water) is supported by positive difference density peaks. While the resolution of the data does not allow to see density for water molecules directly, the presence of coordinated water at the Mg^{2+} ions was deduced from residual difference density peaks in the F_o-F_c maps if waters were omitted during the data analysis and from structure comparison with *S. cerevisiae* F_1 structures at higher resolution (Bowler et al., 2007; Kabaleeswaran et al., 2006).

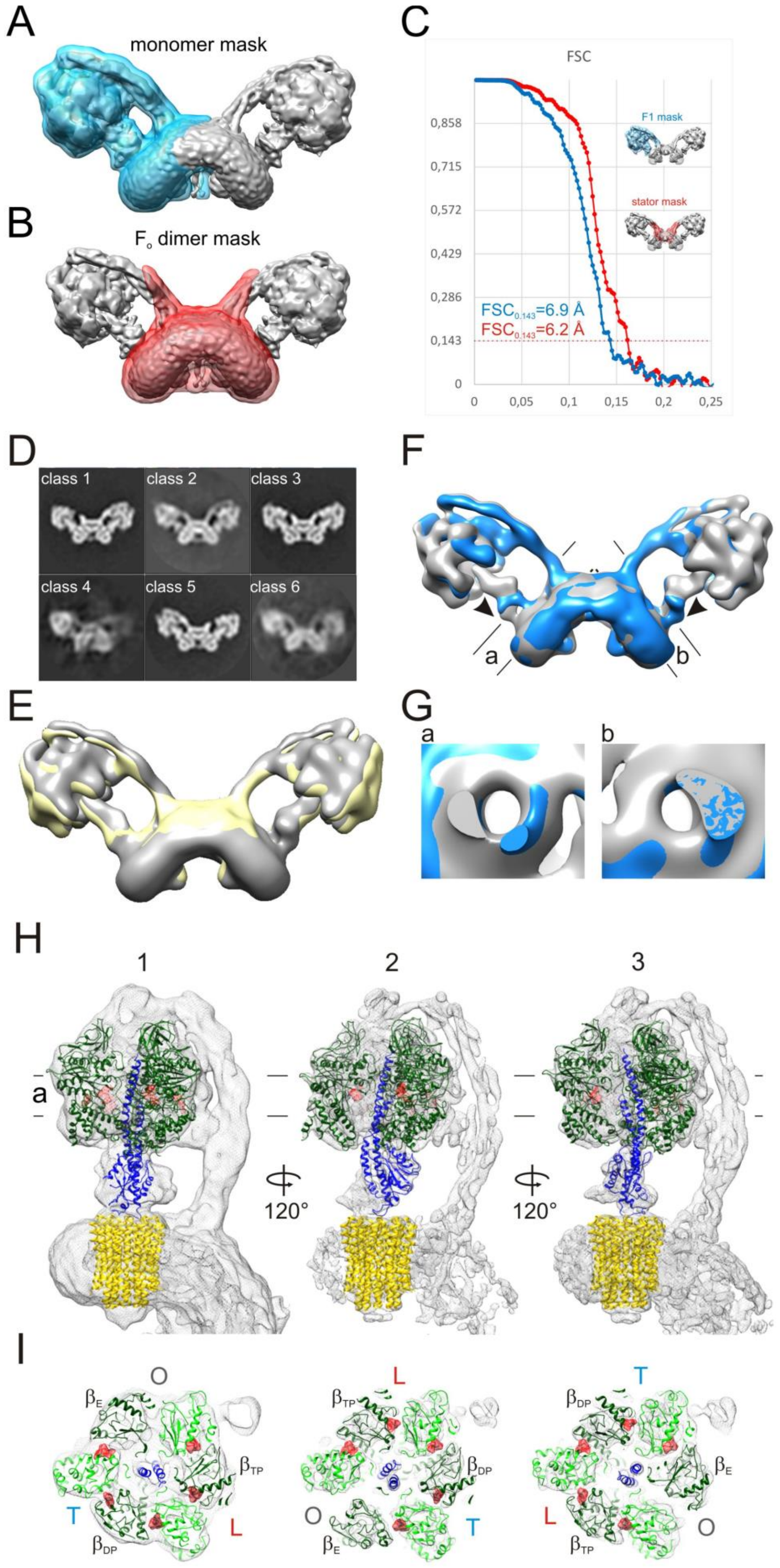

A
monomer mask
B
F$_o$ dimer mask
C
FSC
F1 mask
stator mask
0,858
0,715
0,572
0,429
0,286
0,143
0
0 0,05 0,1 0,15 0,2 0,25
FSC$_{0.143}$=6.9 Å
FSC$_{0.143}$=6.2 Å
D
class 1
class 2
class 3
class 4
class 5
class 6
E
F
a
b
G
a
b
H
1
2
3
a
120°
120°
I
O
β_E
β_{TP}
T
L
β_{DP}
L
β_{TP}
β_{DP}
O
T
β_E
T
β_{DP}
β_E
L
O
β_{TP}

Figure S3, related to Experimental Procedures, Figure 1 and Figure 2. Conformational variability in the F_1F_o dimer. (**A-C**) Use of soft edge masks to compensate for conformational variability and estimation of local resolution of the *Y. lipolytica* ATP synthase dimer. The resolution of 3D reconstructions is limited by conformational variability and structural flexibility of the V-shaped dimers. To further improve the resolution of the reconstructed model, customized soft edge masks were used which only include selected subvolumes. (**A**) Mask that includes an F_1F_o monomer (blue) and (**B**) mask of the F_o-dimerization interface (red). Both masks were introduced during the last 3D refinement iterations in RELION-1.3 (Scheres, 2012). (**C**) Resolution of the masked F_1 head (blue) and masked F_o stator region (red) was determined by Fourier Shell Correlation (FSC). The resolution of F_1 and F_o stator was improved to 6.9 Å and 6.2 Å, respectively. (**D-G**) 3D classification of *Y. lipolytica* ATP synthase dimers reveals variation in the angle of the two monomers and asymmetric resting positions of two rotor elements within the dimer. (**D**) RELION-1.3 was used for 3D classification of 48,207 particles (selected after 2D classification). Six classes were generated. Class 1 (16,286 particles), class 3 (11,346 particles) and class 5 (11,047 particles) are very similar. Class 5 shows inverted handedness. Particles in class 2, 4 and 6 (9,628 particles total) do not form well shaped 3D volumes. All particles of class 2, 4 and 6 were discarded to clean the dataset before final 3D refinement of all particles. (**E**) Difference in the angle of the V-shaped dimer between class 1 (grey) and class 3 (yellow). (**F**) 38,679 particles were used for 3D reconstruction. The aligned dataset was used as starting point for 3D classification with an angular sampling interval of 1.8° and local angular search range of ±5° without applied symmetry to determine the structural heterogeneity of the dataset. Nine classes were generated to allow for structural flexibility during 3D classification. Class 4 (grey, 7,259 particles) and class 8 (blue, 6,862 particles) are superposed. (**G**) Slices through the maps in (F) (marked a and b) indicate variable rotor positions: In contrast to class 4 (grey) class 8 (blue) shows an asymmetric resting position of the two rotor elements in the dimer. The structural asymmetry of rotor positions within the dimer in the dataset suggests that the two F_1F_o monomers in an ATP synthase dimer are not functionally coupled. (**H, I**) Three different conformational states of the *Y. lipolytica* ATP synthase monomer complexes. The states are separated by 3D classification with an angular sampling interval of 1.8° and local angular search range of ±5° combined with an F_1F_o monomer soft edge mask (A-C). 38,679 particles were used for 3D classification. The majority of particles were sorted into subclass 2 and 3. The three classes are *subclass 1*: 5,621 (15.3%), *subclass 2*: 16,671 (45.5%) and *subclass 3*: 14,379 (39.2%) particles. Particles in each subclass were used for a final 3D refinement in RELION-1.3 (Scheres, 2012). (**H**) The three final maps are distinguished by 120° rotations of the central stalk subunit γ (shown in blue). (**I**) Cross sections of the three states at the level of the nucleotide binding sites. The α/β headpiece (light green/dark green) shows different conformations corresponding to the 'Boyer-Walker' states described as *open* (β_E), *loose* (β_{TP}) and *tight* (β_{DP}) (Abrahams et al., 1994; Boyer, 1997). The view from the matrix side shows the counterclockwise transition of the α/β subunits in synthesis direction. The nucleotides are indicated in red, according to the *Y. lipolytica* F_1c_{10} structure (this work). The *c*-ring is indicated in yellow.

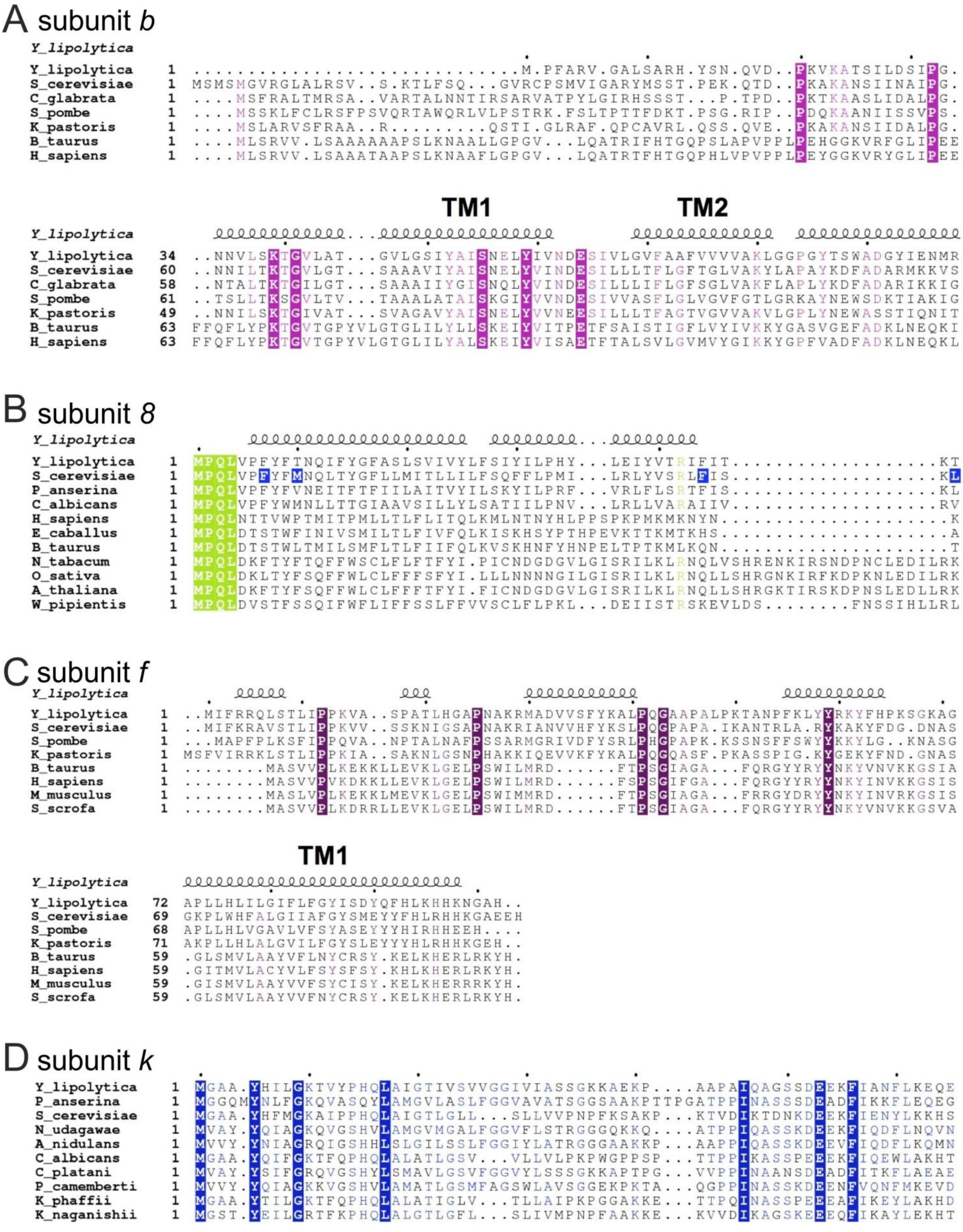
A subunit b
Y_lipolytica
Y_lipolytica 1 ..........................M.PFARV.GALSARH.YSN.QVD..PKVKATSILDSIPG.
S_cerevisiae 1 MSMSMGVRGLALRSV..S.KTLFSQ...GVRCPSMVIGARYMSST.PEK.QTD..PKAKANSIINAIPG.
C_glabrata 1 ....MSFRALTMRSA..VARTALNNTIRSARVATPYLGIRHSSST...P.TPD..PKTKAASLIDALPG.
S_pombe 1 ....MSSKLFCLRSFPSVQRTAWQRLVLPSTRK.FSLTPTTFDKT.PSG.RIP..PDQKAANIISSVPS.
K_pastoris 1 ....MSLARVSFRAA..R........QSTI.GLRAF.QPCAVRL.QSS.QVE..PKAKANSIIDALPG.
B_taurus 1 ....MLSRVV.LSAAAAAAPSLKNAALLGPGV...LQATRIFHTGQPSLAPVPPLPEHGGKVRFGLIPEE
H_sapiens 1 ....MLSRVV.LSAAATAAPSLKNAAFLGPGV...LQATRTFHTGQPHLVPVPPLPEYGGKVRYGLIPEE
TM1
TM2
Y_lipolytica
Y_lipolytica 34 ..NNVLSKTGVLAT...GVLGSIYAISNELYIVNDESIVLGVFAAFVVVVAKLGGPGYTSWADGYIENMR
S_cerevisiae 60 ..NNILTKTGVLGT...SAAAVIYAISNELYVINDESILLLTFLGFTGLVAKYLAPAYKDFADARMKKVS
C_glabrata 58 ..NTALTKTGILGT...SAAAIIYGISNQLYVINDESILLLIFLGFSGLVAKFLAPLYKDFADARIKKIG
S_pombe 61 ..TSLLTKSGVLTV...TAAALATAISKGIYVVNDESIVVASFLGLVGVFGTLGRKAYNEWSDKTIAKIG
K_pastoris 49 ..NNILSKTGIVAT...SVAGAVYAISNELYVVNEESILLLTFAGTVGVVAKVLGPLYNEWASSTIQNIT
B_taurus 63 FFQFLYPKTGVTGPYVLGTGLILYLLSKEIYVITPETFSAISTIGFLVYIVKKYGASVGEFADKLNEQKI
H_sapiens 63 FFQFLYPKTGVTGPYVLGTGLILYALSKEIYVISAETFTALSVLGVMVYGIKKYGPFVADFADKLNEQKL
B subunit 8
Y_lipolytica
Y_lipolytica 1 MPQLVPFYFTNQIFYGFASLSVIVYLFSIYILPHY...LEIYVTRIFIT..................KT
S_cerevisiae 1 MPQLVPFFYFMNQLTYGFLLMITLLILFSQFFLPMI...LRLYVSRLFIS..................KL
P_anserina 1 MPQLVPFYFVNEITFTFIILAITVYILSKYILPRF...VRLFLSRTFIS..................KL
C_albicans 1 MPQLVPFYWMNLLTTGIAAVSILLYLSATIILPNV...LRLLVARAIIV..................RV
H_sapiens 1 MPQLNTTVWPTMITPMLLTLFLITQLKMLNTNYHLPPSPKPMKMKNYN...................K
E_caballus 1 MPQLDTSTWFINIVSMILTLFIVFQLKISKHSYPTHPEVKTTKMTKHS...................A
B_taurus 1 MPQLDTSTWLTMILSMFLTLFIIFQLKVSKHNFYHNPELTPTKMLKQN...................T
N_tabacum 1 MPQLDKFTYFTQFFWSCLFLFTFYI.PICNDGDGVLGISRILKLRNQLVSHRENKIRSNDPNCLEDILRK
O_sativa 1 MPQLDKLTYFSQFFWLCLFFFSFYI.LLLNNNNGILGISRILKLRNQLLSHRGNKIRFKDPKNLEDILRK
A_thaliana 1 MPQLDKFTYFSQFFWLCLFFFTFYI.FICNDGDGVLGISRILKLRNQLLSHRGKTIRSKDPNSLEDLLRK
W_pipientis 1 MPQLDVSTFSSQIFWFLIFFSSLFFVVSCLFLPKL...DEIISTRSKEVLDS........FNSSIHLLRL
C subunit f
Y_lipolytica
Y_lipolytica 1 ..MIFRRQLSTLIPPKVA..SPATLHGAPNAKRMADVVSFYKALPQGAAPALPKTANPFKLYYRKYFHPKSGKAG
S_cerevisiae 1 ..MIFKRAVSTLIPPKVV..SSKNIGSAPNAKRIANVVHFYKSLPQGPAPA.IKANTRLA.RYKAKYFDG.DNAS
S_pombe 1 ...MAPFPLKSFIPPQVA..NPTALNAFPSSARMGRIVDFYSRLPHGPAPK.KSSNSFFSWYKKKYLG..KNASG
K_pastoris 1 MSFVIRRKLSTLIPPKIA..SAKNLGSNPHAKKIQEVVKFYKALPQGQASF.PKASSPIG.KYGEKYFND.GNAS
B_taurus 1 ........MASVVPLKEKKLLEVKLGELPSWILMRD......FTPSGIAGA...FQRGYYRYYNKYVNVKKGSIA
H_sapiens 1 ........MASVVPVKDKKLLEVKLGELPSWILMRD......FSPSGIFGA...FQRGYYRYYNKYINVKKGSIS
M_musculus 1 ........MASLVPLKEKKLMEVKLGELPSWIMMRD......FTPSGIAGA...FRRGYDRYYNKYINVRKGSIS
S_scrofa 1 ........MASVVPLKDRRLLEVKLGELPSWILMRD......FTPSGIAGA...FQRGYYRYYNKYVNVKKGSVA
TM1
Y_lipolytica
Y_lipolytica 72 APLLHLILGIFLFGYISDYQFHLKHHKNGAH..
S_cerevisiae 69 GKPLWHFALGIIAFGYSMEYYFHLRHHKGAEEH
S_pombe 68 APLLHLVGAVLVFSYASEYYYHIRHHEEH....
K_pastoris 71 AKPLLHLALGVILFGYSLEYYYHLRHHKGEH..
B_taurus 59 .GLSMVLAAYVFLNYCRSY.KELKHERLRKYH.
H_sapiens 59 .GITMVLACYVLFSYSFSY.KHLKHERLRKYH.
M_musculus 59 .GISMVLAAYVVFSYCISY.KELKHERRRKYH.
S_scrofa 59 .GLSMVLAAYVVFNYCRSY.KELKHERLRKYH.
D subunit k
Y_lipolytica 1 MGAA.YHILGKTVYPHQLAIGTIVSVVGGIVIASSGKKAEKP....AAPAIQAGSSDEEKFIANFLKEQE
P_anserina 1 MGGQMYNLFGKQVASQYLAMGVLASLFGGVAVATSGGSAAKPTTPGATPPINASSSDEADFIKKFLEQEG
S_cerevisiae 1 MGAA.YHFMGKAIPPHQLAIGTLGLL...SLLVVPNPFKSAKP...KTVDIKTDNKDEEKFIENYLKKHS
N_udagawae 1 MVAY.YQIAGKQVGSHVLAMGVMGALFGGVFLSTRGGGQKKQ....ATPPIQASSKDEEKFIQDFLNQVN
A_nidulans 1 MVVY.YNIAGRQIGSHHLSLGILSSLFGGIYLATRGGGAAKKP...AAPPIQASSKDEEVFIQDFLKQMN
C_albicans 1 MGAA.YQIFGKTFQPHQLALATLGSV...VLLVLPKPWGPPSP...TTPPIKASSPEEEKFIQEWLAKHT
C_platani 1 MVAY.YSIFGRQVGSHYLSMAVLGSVFGGVYLSSSGKKAPTPG...VVPPINAANSDEADFITKFLAEAE
P_camemberti 1 MVVY.YQIAGKKVGSHVLAMATLGSMFAGSWLAVSGGEKPKTA...QGPPINASSKDEENFVQNFMKEVD
K_phaffii 1 MGAA.YTILGKTFQPHQLALATIGLV...TLLAIPKPGGAKKE...TTPQINASSPEEEAFIKEYLAKHD
K_naganishii 1 MGST.YEILGRTFKPHQLALGTLGFL...SLIVMPNPFAAKKE...KVVDIKAGSKEEEQFIKAYLEKHT

Figure S4, related to Figure 3, Figure 5 and Figure 6. Sequence alignments of subunits *b*, *8*, *f* and *k*. (**A**) Subunit *b*. The alignment was created using the sequences from *Yarrowia lipolytica, Saccharomyces cerevisiae, Candida glabrata*, *Schizosaccharomyces pombe*, *Komagataella pastoris*, *Bos taurus* and *Homo sapiens*. (**B**) Subunit *8*. The alignment was created by using the sequences from *Yarrowia lipolytica*, *Saccharomyces cerevisiae, Podospora anserina*, *Candida albicans*, *Homo sapiens*, *Equus caballus* and *Bos taurus* with subunit *b* from *Nicotiana tabacum*, *Oryza sativa*, *Arabidopsis thaliana* and *Wolbachia pipientis*. Crosslinking residues (**Figure S6**) are represented in blue boxes. (**C**) Subunit *f*. The alignment was created using the sequences from *Yarrowia lipolytica*, *Saccharomyces cerevisiae*, *Schizosaccharomyces pombe*, *Komagataella pastoris*, *Bos taurus*, *Homo sapiens*, *Mus musculus* and *Sus scrofa*. (**D**) Subunit *k*. The alignment was created using the sequences from *Yarrowia lipolytica*, *Podospora anserina*, *Saccharomyces cerevisiae*, *Aspergillus nidulans* FGSC A4, *Neosartorya udagawae*, *Candida albicans* P94015, *Ceratocystis platani*, *Penicillium camemberti*, *Komagataella phaffii* GS115 and *Kazachstania naganishii* CBS 8797. The secondary structure is indicated according to the model based on the cryoEM structure. The alignments do not show the complete sequences (except subunit *f*).

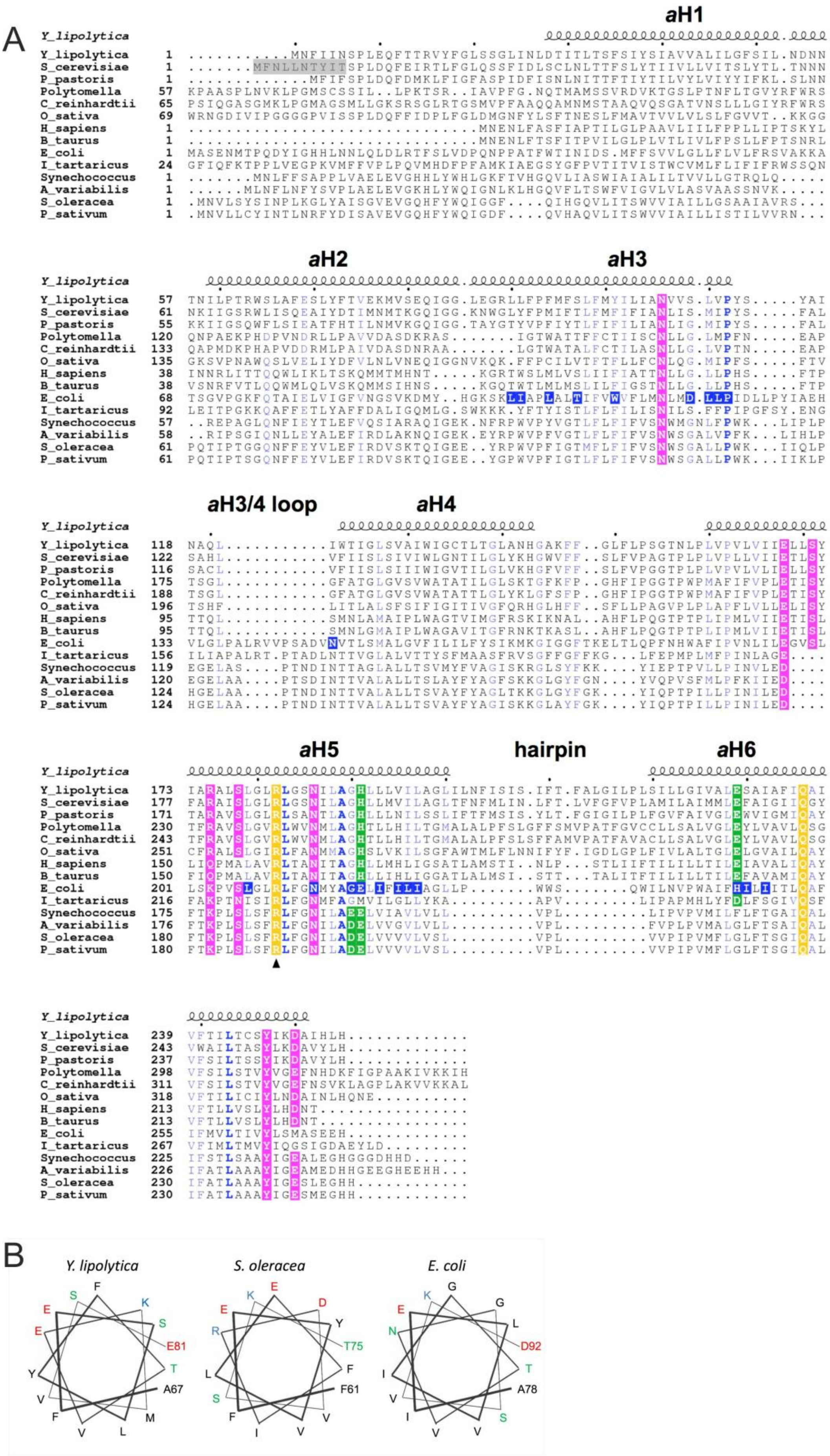
A
aH1
aH2
aH3
aH3/4 loop
aH4
aH5
hairpin
aH6
Y_lipolytica
S_cerevisiae
P_pastoris
Polytomella
C_reinhardtii
O_sativa
H_sapiens
B_taurus
E_coli
I_tartaricus
Synechococcus
A_variabilis
S_oleracea
P_sativum
B
Y. lipolytica
S. oleracea
E. coli
E81
A67
T75
F61
D92
A78

Figure S5, related to Figure 4. Sequence alignment and helical wheels of subunit *a*. (**A**) The alignment was generated from sequences of *Yarrowia lipolytica, Saccharomyces cerevisiae, Pichia pastoris, Polytomella* sp. Pringsheim, *Chlamydomonas reinhardtii*, *Oryza sativa*, *Homo sapiens*, *Bos taurus*, *Escherichia coli*, *Ilyobacter tartaricus*, *Synechococcus* sp., *Anabaena variabilis*, *Spinacia oleracea* and *Pisum sativum*. The secondary structure assignment is based on the model of the cryoEM structure. The interchangeable residue pairs *a*Arg175/*a*Gln236 and *a*Glu229/*a*His191 are yellow and green, respectively (Cain, 2000; Eya et al., 1991; Lightowlers et al., 1987). Conserved charged and polar residues are highlighted in pink. The black arrowhead marks the strictly conserved arginine essential for coupled proton translocation. Crosslinking residues are represented in blue boxes. The signal sequence of *S. cerevisiae* is represented as a bar in light grey. (**B**) Helical wheels for the predicted helix *a*H2 show evidence for a conserved amphipathic helix in all species in mitochondria (exemplified by *Y. lipolytica*), chloroplasts (*S. oleracea*) and bacteria (*E. coli*). Hydrophobic residues are shown in black, positively charged in blue, negatively charged in red, polar in green.

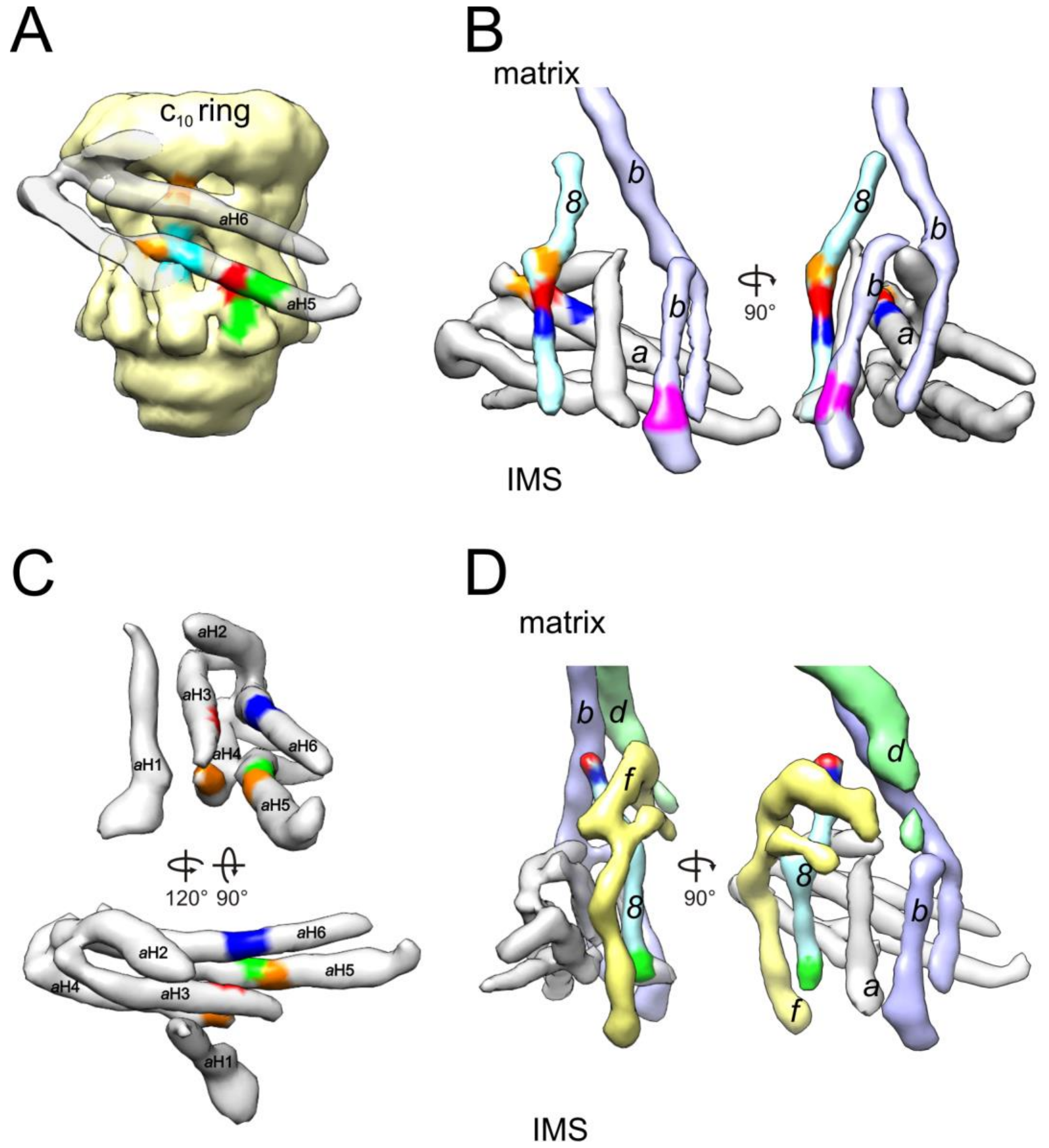

Figure S6, related to Figure 6. Cys-Cys crosslink positions in *E. coli* and *S. cerevisiae*. To verify the assignment of α-helices in the F_o region of the *Y. lipolytica* F_1F_o dimer, we compared available crosslink studies for *E. coli* and *S. cerevisiae* (DeLeon-Rangel et al., 2013; Jiang and Fillingame, 1998; Schwem and Fillingame, 2006; Stephens et al., 2003) with our ATP synthase dimer model. By using multiple pairwise sequence alignment ESPript (Robert and Gouet, 2014) we generated a homology model and assigned corresponding amino acids in **Table S3**. To visualize the crosslink positions, the crosslinks are shown as color pairs on the segmented cryo-EM volumes as indicated in **Table S3**. (**A**) Cys-Cys crosslink positions of subunit *a*H5 to the c_{10} ring. The crosslink pairs follow the curvature of *a*H5 around the c_{10} ring. (**B**) Subunit *8* is in close proximity to *a*H2 and *a*H3. Val111 and Pro112 of subunit *a* are located in the flexible loop region between *a*H3 and *a*H4, which lies in close proximity to Ser55 from subunit *b* (pink). (**C**) Crosslink positions in the horizontal double hairpin between *a*H3 and *a*H5 (red/green), *a*H4 and *a*H5 (orange), *a*H3 and *a*H6 (red/blue) and *a*H5 and *a*H6 (green/blue). (**D**) Crosslinks between subunit *8* and subunit *b*, *d* and *f* (red/blue) on the matrix side. Our model places the N terminus of subunit *8* (green) and the C terminus of *f* in close proximity at the IMS, which also matches with *S. cerevisiae* crosslinks (Stephens et al., 2003).

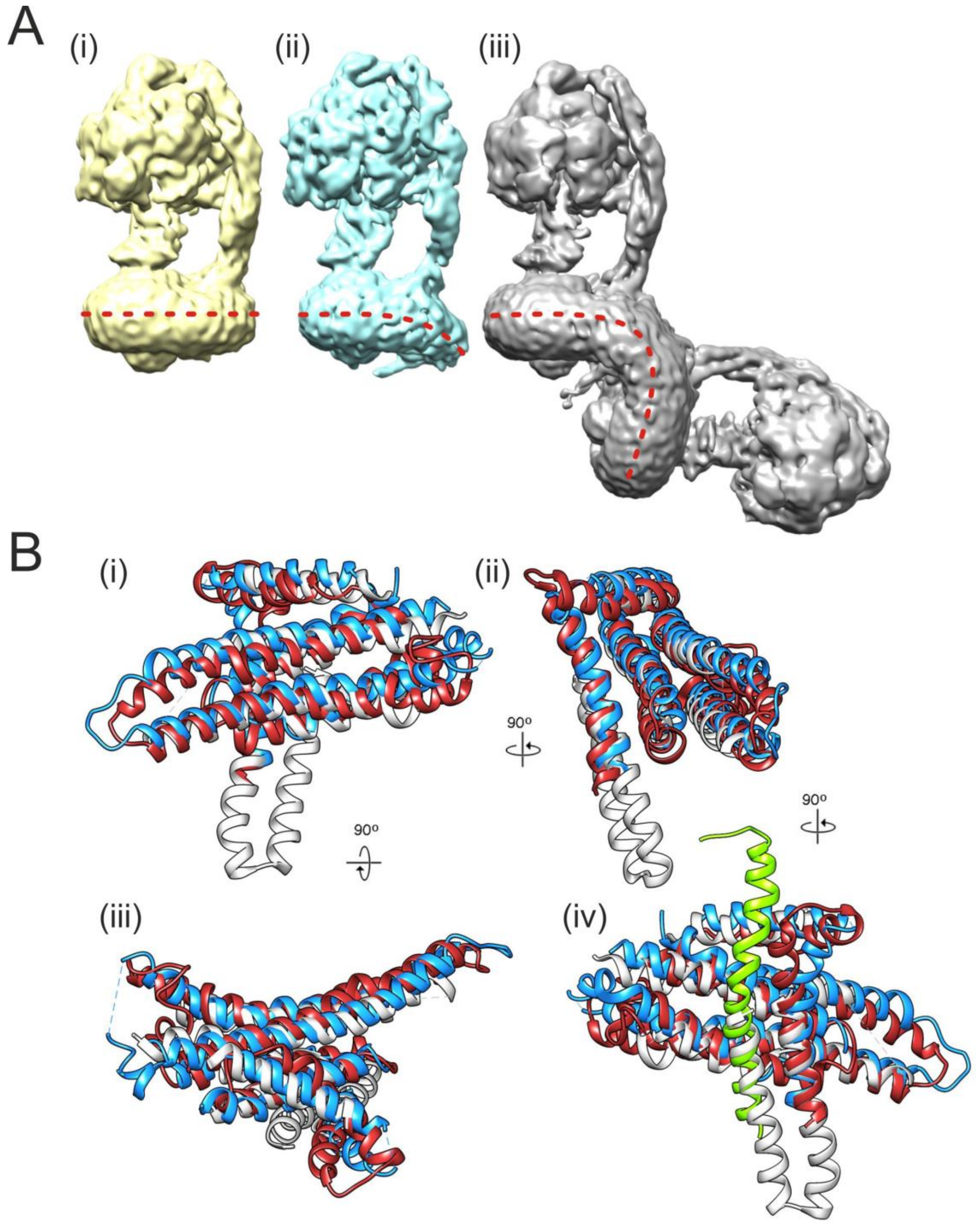

Figure S7, related to Figure 4 and Figure 5. (**A**) Subunit *e* and *g* bend the inner mitochondrial membrane. (i) Monomeric ATP synthase of *Y. lipolytica* (8.4 Å). Subunit *e* and *g* and the fungi-specific subunit *k* are lost during purification with the detergent dodecyl maltoside (**Table S1**). The detergent belt forms a level, unbent ring around the F_o sector. (ii) Monomeric bovine ATP synthase (Zhou et al., 2015): EMD-3165, 7.4 Å. The dimer-specific subunits *e* and *g* were retained during purification. The detergent belt bends away from the *c*-ring, probably due to the presence of subunits *e* and *g*. (iii) Dimeric ATP synthase of *Y. lipolytica* at 7.8 Å resolution. Subunits *e, g* and *k* were retained during purification in the mild detergent digitonin (**Table S1**). The F_o sector is strongly bent, presumably by subunits *e* and *g*. This might force both halves of the dimer into a position where subunit *f* can form the dimer contact. (**B**) Comparison of subunit *a* from different species. *Y. lipolytica* mitochondria, blue (this work); *Paracoccus denitrificans* (5DN6), white (Morales-Rios et al., 2015); bovine heart

mitochondria (5ARA), brown (Zhou et al., 2015). (i) View from the *c*-ring towards the *a*H5-*a*H6 hairpin. The matrix side is on top. (ii) Side view. (iii) Top view from the matrix side. (iv) View from the dimer interface. Subunit *8* of *Y. lipolytica* is shown in green. This subunit superposes well on an unassigned trans-membrane helix in the *P. denitrificans* ATP synthase. We identify this unassigned helix as belonging to one of the two bacterial *b*-subunits. There is no density for the second *b*-subunit trans-membrane helix in the *P. denitrificans* X-ray map.

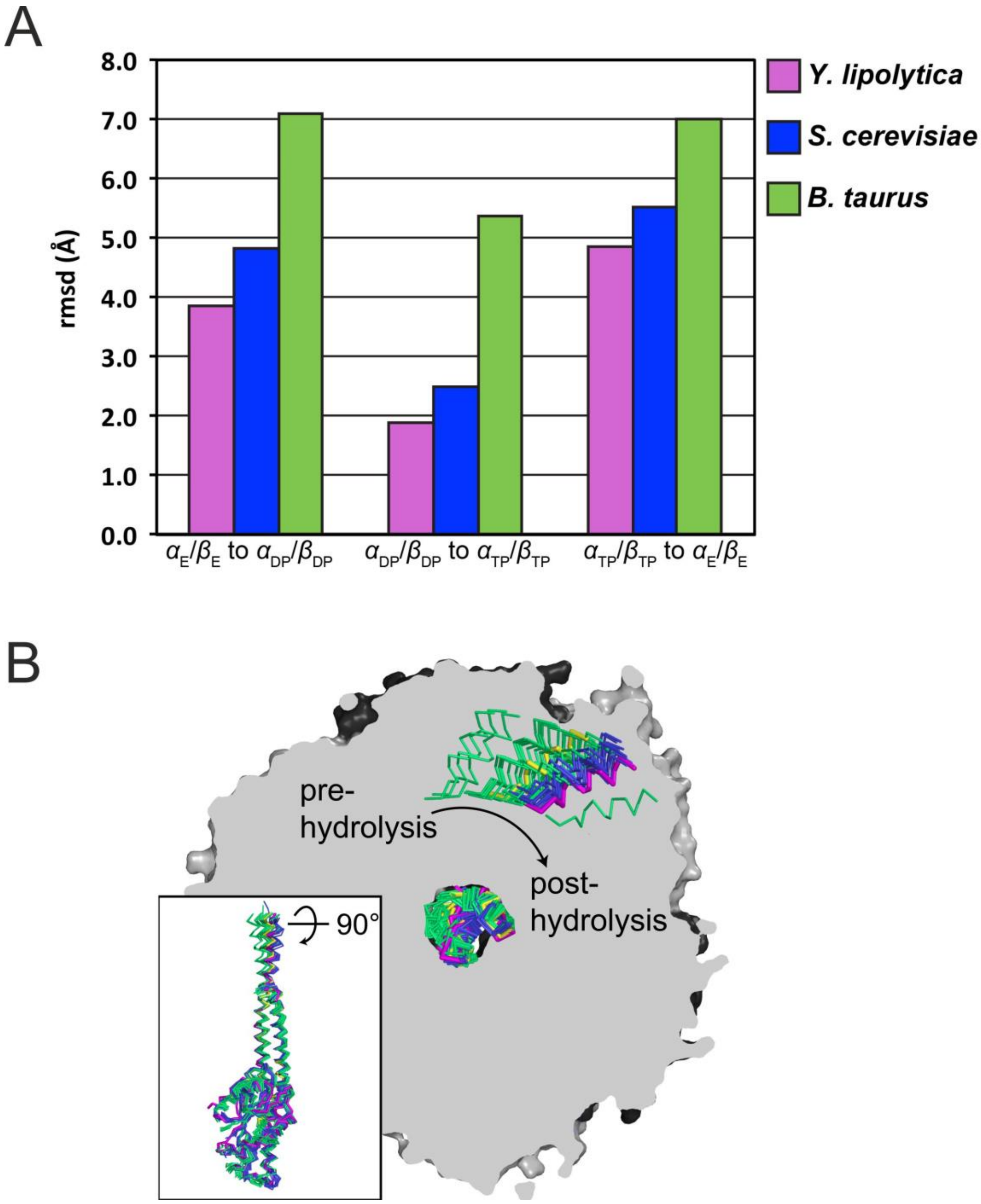

Figure S8, related to Figure 1. Comparison of the *Y. lipolytica* F_1c_{10} structure with *B. taurus* and *S. cerevisiae* F_1 and F_1c_{10} structures. (**A**) Structural differences between α/β heterodimers in *Y. lipolytica*, *S. cerevisiae* and *B. taurus* ATP synthase. To compare the structural differences within the α/β heterodimer, including the catalytic site in the β-subunits, the root mean square deviation (rmsd) values were calculated using the α/β heterodimers of the F_1 sectors from *Y. lipolytica* F_1c_{10} structure (*YL*F_1c_{10}, this work), *S. cerevisiae* structure PDB: 2HLD (Kabaleeswaran et al., 2006) and the F_1 reference structure, PDB: 1BMF (Abrahams et al., 1994). The rmsd values of the three different conformational Boyer states *open*, *tight* and *loose* (Boyer, 1997), corresponding to β_E, β_{DP} and β_{TP} (Abrahams et al., 1994), respectively, are largest within the structure of the bovine F_1 (PDB: 1BMF), while the inhibitor-free *YL*F_1c_{10}

exhibits the smallest overall conformational differences. (B) Rotational positioning of the *Yarrowia lipolytica* F_1c_{10} ATP synthase γ-subunit and P-loop in comparison with other F_1 crystal structures from *Saccharomyces cerevisiae* and *Bos taurus*. The angular position of the γ-subunit relative to the α_3/β_3 head piece was visualized by aligning the structures on the γ-subunits (the overall alignment is shown in the inset in the bottom left corner; see Methods for details) and comparing the angular position of the P-loops in the β_{DP} site of the F_1 sector (depicted as colored ribbons). The following structures were compared from *B. taurus* (green): PDBs: 1BMF, 1COW, 1E1Q, 1E1R, 1E79, 1EFR, 1H8E, 1H8H, 1NBM, 1W0J, 1W0K, 2CK3, 2JDI, 2JIZ, 2JJ1, 2JJ2, 2V7Q, 2WSS, 2XND, 4ASU, 4TSF, 4TT3, 4YXW, and 4Z1M (Abrahams et al., 1996; Abrahams et al., 1994; Bason et al., 2014, 2015; Bowler et al., 2006, 2007; Braig et al., 2000; Gledhill et al., 2007; Kagawa et al., 2004; Menz et al., 2001a; Menz et al., 2001b; Orriss et al., 1998; Rees et al., 2009; Rees et al., 2012; van Raaij et al., 1996; Watt et al., 2010). The reference structure (Abrahams et al., 1994) is highlighted in yellow. *S. cerevisiae* (blue), PDBs: 2HLD, 2WPD, 2XOK, 3FKS, 3OE7, 3OEE, 3OEH, 3OFN, 3ZIA, 3ZRY (Arsenieva et al., 2010; Dautant et al., 2010; Giraud et al., 2012; Kabaleeswaran et al., 2006; Kabaleeswaran et al., 2009; Robinson et al., 2013; Stock et al., 1999). Purple: *Y. lipolytica*. The view is from the membrane, the *Y. lipolytica* F_1 sector is depicted as a cross-section at the approximate level of the P-loop. The ATP hydrolysis rotation direction of α_3/β_3 relative to the central stalk is indicated by an arrow. The *Y. lipolytica* P-loop is shifted the furthest to a post-hydrolysis state, as compared to the majority of the *S. cerevisiae* and *B. taurus* F_1 structures, with only one exception, the *B. taurus* post-hydrolysis (pre-product release) structure 4ASU (Rees et al., 2012).

Supplementary Movies

Movie S1, related to Figure 2. *Y. lipolytica* ATP synthase dimer EM map. Cartoon model of all assigned protein subunits is shown in one of the two monomers. Color code is as in **Figure 2**.

Movie S2, related to Figure 1. Morphed conformational changes in *Y. lipolytica* F_1-ATP synthase. Animation based on a morph between the three different conformational states observed in the *Y. lipolytica* F_1c_{10} crystal structure. Subunit representations and coloring as follows: α, dark green or dark grey ribbon; β, light green or light grey ribbon; γ, blue surface; δ, cyan surface; ε, white surface. MgADP is represented as stick-ball model with C, N, O, P, and Mg colored in yellow, blue, red, orange, and magenta, respectively. The corner points for the morphs were generated by triplicating the entire molecule and then superposing the α_{DP} subunit of the first copy and the α_{TP} subunit of the second copy on the α_E subunit of the original structure.

Movie S3, related to Figure 1. Morphed conformational changes in the α/β heterodimer. Alternative view on the morphed animation shown also in **Movie S2**. For clarity, only one catalytic α/β heterodimer is shown, together with the rotating stalk containing subunits γ, δ, and ε. Representations and coloring as in **Movie S2**.

Movie S4, related to Figure 1. Comparison of yeast and bovine ATP synthase. Superposed morphs of a catalytic α/β heterodimer from *B. taurus* (PDB: 1BMF; α, dark gray; β, light gray) (Abrahams et al., 1994) and *Y. lipolytica* (this work, α, dark green; β, light green). The α-subunits were used as reference for both morphing and superposition. The conformations of the β-subunits are most divergent in the β_{DP} states, and more similar in the β_{TP} and β_E states. Also, the bovine structure undergoes larger overall conformational changes than the *Y. lipolytica* structure.

Movie S5, related to Figure 1. Comparison of *S. cerevisiae* and *Y. lipolytica* α/β heterodimers. Superposed morphs of a catalytic α/β heterodimer from *S. cerevisiae* (PDB: 2HLD; α, dark gray; β, light gray) (Kabaleeswaran et al., 2006) and *Y. lipolytica* (this work, α, dark green; β, light green). The α-subunits were used as reference for both morphing and superposition.

Supplementary Tables

Table S1, related to Figure 2 and Figure 5. Protein subunits in *Y. lipolytica* monomeric and dimeric ATP synthase, analyzed by LC-MS[*].

	subunit	dimer	monomer	size (kDa)	Dimer sequence coverage (%)	Dimer score	Monomer sequence coverage (%)	Monomer score
F1	*α*	+ [a,b]	+ [a,b]	58	50.9[a], 62.7[b]	1993.48[a], 1918.78[b]	58[a], 52.4[b]	2158.7[a], 1415.97[b]
	β	+ [a,b]	+ [a,b]	54.5	55.2[a], 57.2[b]	1716.49[a], 1160.33 [b]	61.7[a], 38.7[b]	1727.06[a], 726.89[b]
	γ	+ [a,b]	+ [a,b]	32.3	37.5[a], 41.30[b]	664.48[a], 390.50 [b]	46.4[a], 43.7[b]	838.31[a], 473.33[b]
	δ	n.d.	n.d.	-	-	-	-	-
	ε	n.d.	n.d.	-	-	-	-	-
	OSCP	+ [a,b]	+ [a,b]	22.8	25.7[a], 32.2[b]	219.4[a], 299.74 [b]	48.6[a], 32.7[b]	512.61[a] , 307.53[b]
Fo	***a***	+ [a,b]	+ [a,b]	27.8	7.5[a], 7.1[b]	141.9[a]5, 88.2 [b]	7.1[a], 3.1[b]	116.46[a], 80.84[b]
	b	+ [a,b]	+ [a,b]	24	46.1[a], 34.6[b]	556.2[a], 264.85 [b]	48.8[a], 12.9[b]	589.47[a], 133.45[b]
	c	n.d.	n.d.		-	-		
	d (F6)	+ [a,b]	+ [a,b]	19.8	46.6[a], 46.6[b]	451.47[a], 289.53 [b]	47.2[a], 43.8[b]	439.34[a], 285.17[b]
	f	+ [a,b]	+ [a,b]	11.4	42.7[a], 47.6[b]	473.38[a], 322.14 [b]	41.7[a], 47.6[b]	347.87[a], 337.87[b]
	h	n.d.	n.d.	-	-	-	-	-
	i	+ [b]	+ [b]	6.9	59.7[b]	132.48[b]	43.5[b]	136.8[b]
	8 (A6L)	n.d.	n.d.	-	-	-	-	-
	e	+ [a,b]	n.d.	10	48.9[a], 28.9[b]	158.46[a], 142.68[b]	-	-
	g	+ [a,b]	n.d.	15.1	22.5[a], 49.3[b]	156.25[a], 284.93[b]	-	-
	k	+ [a]	n.d..	7.3	37.1[a]	150.8[a]	-	-

[a]Trypsin, [b]Chymotrypsin, n.d., not detected, ( ) alternative nomenclature for bovine,
[*]for details see Supplementary Experimental Procedures

Table S2, related to Figure 1. Comparison of the *Y. lipolytica* α/β heterodimers with other crystal structures. Structures of the three α/β heterodimers in the F_1 sectors of ATP synthase from *Y. lipolytica* (this work), *B. taurus* PDB: 1BMF (Abrahams et al., 1994) and *S. cerevisiae*, PDB: 2HLD (Kabaleeswaran et al., 2006) were compared. The root mean square deviation (rmsd)-values (Å) between the main chain atoms of the respective heterodimers were determined with the 'align' function in PyMOL (Schrödinger, LLC).

		Y. lipolytica			***B. taurus***			***S. cerevisiae***	
		α_E/β_E	α_{DP}/β_{DP}	α_{TP}/β_{TP}	α_E/β_E	α_{DP}/β_{DP}	α_{TP}/β_{TP}	α_E/β_E	α_{TP}/β_{TP}
Y. lipolytica	α_E/β_E								
	α_{DP}/β_{DP}	3.85							
	α_{TP}/β_{TP}	4.84	1.89						
B. taurus	α_E/β_E	1.89	4.28	5.35					
	α_{DP}/β_{DP}	5.54	2.63	2.70	7.11				
	α_{TP}/β_{TP}	5.32	2.88	2.07	7.00	5.38			
S. cerevisiae	α_E/β_E	1.28	4.27	5.31	2.95	6.04	6.05		
	α_{DP}/β_{DP}	4.11	1.97	2.46	4.90	3.11	3.23	4.84	
	α_{TP}/β_{TP}	4.96	2.44	1.75	5.71	3.43	2.67	5.54	2.49

Table S3, related to Figure 6. Cys-Cys crosslink positions in *E. coli, S. cerevisiae* and *Y. lipolytica*. List of residue pairs that form Cys-substitution crosslinks in *E. coli* and *S. cerevisiae*. Homologous residues in *Y. lipolytica* were identified by sequence alignments (**Figures S4** and **Figure S5**). For each crosslink study (A-D) a color code was assigned to show the position of each homologous crosslink pair in the segmented EM subvolume of *Y. lipolytica* (**Figure S6**).

	E. coli[1]	*Y. lipolytica*
A	*a* L207/*c* I55	*a* L179/c_{10} L53
	a N214/*c* A62	*a* N186/c_{10} A60
	a N214/*c* M65	*a* N186/c_{10} L63
	a I221/*c* G69	*a* L193/c_{10} M67
	a I223/*c* L72	*a* V195/c_{10} F70
	a L224/*c* Y73	*a* I196/c_{10} L71
	a I225/*c* Y73	*a* L197/c_{10} L71
	***E. coli*[2]**	***Y. lipolytica***
B	*a* P122/*b* N4	*a* P112/*b* S55
	a L121/*b* N4	*a* V111/*b* S55
	a W111/*b* F14	*a* Y101/*8* L20
	a W111/*b* V15	*a* Y101/*8* S21
	a T107/*b* V18	*a* S97/*8* V24
	a L104/*b* M22	*a* F94/*8* S28
	a I101C/*b* W26	*a* P91/*8* L32
	a L100C/*b* W26	*a* L90/*8* L32
	***E. coli*[3]**	***Y. lipolytica***
C	*a* D119/*a* G218	*a* S109/*a* G190
	a L120/*a* G218	*a* L110/*a* G190
	a N148/*a* E219	*a* I122/*a* H191
	a L120/*a* I246	*a* L110/*a* S230
	a L120/*a* H245	*a* L110/*a* E229
	a G218/*a* I248	*a* G190/*a* I232
	***S. cerevisiae*[4]**	***Y. lipolytica***
D	*8* F44/sub. *b* matrix	*8* F44/sub. *b* matrix
	8 F44/sub. *d* matrix	*8* F44/sub. *d* matrix
	8 F44/sub. *f* matrix	*8* F44/sub. *f* matrix
	8 L48/sub. *d* matrix	*8* T48/sub. *d* matrix
	8 L48/sub. *f* matrix	*8* T48/sub. *f* matrix
	8 M10/*a* C23	*8* T10/*a* 23
	8 F7/*a* C23	*8* F7/*a* 23
	8 F7/sub. *f* IMS	*8* F7/sub. *f* IMS

[1](Jiang and Fillingame, 1998)
[2](DeLeon-Rangel et al., 2013)
[3](Schwem and Fillingame, 2006)
[4](Stephens et al., 2003)

SUPPLEMENTARY EXPERIMENTAL PROCEDURES

Isolation of *Yarrowia lipolytica* ATP synthase dimers and monomers. Large-scale fermentation of *Y. lipolytica* and isolation of mitochondrial membranes were carried out as previously described (Hunte et al., 2010). Complex I was removed by metal affinity purification. For long-term storage at -80°C, glycerol was added to a final concentration of 20% (v/v) before solubilized membranes were rapidly frozen in liquid nitrogen. For purification of intact ATP synthase dimers (2*YL*F_1F_o), the sample was diluted 1:1 with Buffer A [30mM MOPS-NaOH pH 7.5, 2 mM $MgCl_2$, 0.5 mM EDTA, 50 mM NaCl, 0.2% (w/v) digitonin (AppliChem, D)] to a total volume of 16 ml. Subsequently 50% (w/w) PEG 6000 was added to a final concentration of 3%. After 15 min incubation on ice the protein precipitate was removed by centrifugation for 15 min at 20,000 x *g*. The supernatant was loaded on a discontinuous glycerol gradient (55-25% in 5% steps) of Buffer A and run at 34.6k rpm with a SW40 rotor (Beckman Coulter, USA) for at least 16 h at 4°C. Fractions containing 2*YL*F_1F_o as judged by BN-PAGE (Wittig et al., 2006) were collected and loaded on a POROS GoPure HQ 50 anion exchange column (Life Technologies, USA) equilibrated with Buffer B (Buffer A with 0.1% (w/v) digitonin) and gradually eluted with Buffer C (Buffer B with 1 M NaCl) using an ÄKTAexplorer chromatography system (GE Healthcare, USA). Fractions containing ATP synthase dimers were collected and concentrated to 0.5 ml (Amicon Ultra-15 centrifugal filters with 100 kDa molecular mass cutoff) and loaded onto a 16/300 Superose 6 gel filtration column (GE Healthcare, USA) equilibrated with Buffer D (Buffer A with 0.05% (w/v) digitonin). Fractions eluting at a retention volume of 10–11 ml were collected and used for further analysis. Protein concentrations were determined with a BCA Protein Assay Kit (Thermo Scientific, D). Purification of the F_1F_o-ATP synthase monomer was carried out as described above, except that digitonin was replaced by 0.1% DDM during all purification steps and the gel filtration step was omitted.

ATPase activity measurements. ATP hydrolysis activity of 2*YL*F_1F_o was measured by an ATP-regenerating assay (Cook et al., 2003). Samples of 15 µg 2*YL*F_1F_o (0.5 mg/ml) were measured in 1 ml reaction volumes. 100 µg/ml yeast polar lipid extract and 12 µg/ml cardiolipin (Avanti, USA) was added and incubated overnight. To remove degradation products, 2*YL*F_1F_o was again applied to a Superose 6 column equilibrated

with Buffer E (Buffer D with yeast polar lipid and cardiolipin). ATP hydrolysis was inhibited by adding oligomycin (0.5 mg/ml in DMSO) to a final concentration of 5 µg/ml and incubation for at least 30 min. 1*YL*F_1F_o monomers were generated by adding DDM (20% (w/v) in water) to a final concentration of 1% (w/v) and 30 min incubation. Complete dissociation of the dimer was confirmed by BN-PAGE and electron microscopy of negatively stained samples.

Single particle cryo-EM. 3 µl of a 2-3 mg/ml 2*YL*F_1F_o (or 1*YL*F_1F_o) solution in 0.05% (w/v) digitonin [or 0.1% (w/v) DDM] was applied to R2/2 holey carbon grids (Quantifoil, DE) and plunge-frozen in liquid ethane using a Vitrobot (FEI) at 100% humidity after blotting for 7 to 9 s. Dose-fractionated 6 s movies of 30 frames with an electron dose of 0.6 electrons per Å^2 per frame were recorded on a Gatan K2 direct electron detector in a JEOL 3200 FSC electron microscope with an in column energy filter operated at zero-loss mode at 300 kV at 1.5–3.5 µm defocus, using a nominal magnification of 20,000x. The calibrated pixel size at was 1.63 Å.

Image processing and map interpretation. Global beam-induced motion was corrected by movie frame processing (Li et al., 2013). The contrast transfer function for each image was determined using CTFFIND3 (Mindell and Grigorieff, 2003) within the RELION-1.3 workflow (Scheres, 2012). 50,118 particles were picked manually with EMAN boxer (Ludtke et al., 1999). A sub-tomogram average of the *Saccharomyces cerevisiae* ATP synthase dimer EMD-2161 (Davies et al., 2012), obtained from tomographic volumes of mitochondrial membranes, was used as an initial reference for refinement in RELION-1.3 (Scheres, 2012). Two-dimensional classification of picked particles was performed in RELION-1.3. 2,774 particles were discarded. Three-dimensional classification of remaining particles was performed to identify structural flexibility and heterogeneity. A total of 38,679 particles belonging to three classes were combined for further processing. Individual frames were B-factor weighted and movements of individual particles were reversed by movie frame correction in RELION-1.3 (Scheres, 2014). A C2-averaged volume was calculated from particles in frames 1–20. A B-factor of -200 Å^2 for map sharpening was determined using the modulation transfer function of the K2 detector. To improve the resolution of individual sub-volumes during 3D refinement, customized soft edge masks for the F_1F_o monomer and the F_o dimer were generated using Chimera and

EMAN2 (Pettersen et al., 2004; Tang et al., 2007). Masks were used in the final iteration steps without imposing symmetry. Gold-standard Fourier shell correlations were calculated from two independently refined data sets to determine the resolution of the complete volume and sub-areas of the structure. For the latter, different regions of two independently refined maps were soft-masked and the resolution determined by Fourier shell correlations using EMAN2 (Tang et al., 2007). Models were fitted with Chimera (Pettersen et al., 2004).

Analysis of conformational states within the EM map. All pre-aligned particles were 3D-classified without symmetry, with an angular sampling interval of 1.8 degrees and local angular search range of +/-5 degrees in RELION-1.3. To analyze each monomer individually we repeated the 3D classification and applied a monomer mask. The classification quickly converged to three classes [named: *subclass 1*: 5,621 (15.3%), *subclass 2*: 16,671 (45.5%) and *subclass 3*: 14,379 (39.2%) particles] (**Figure S3D-G**). Next, we reconstructed 3D maps from all particles in each class using the 40 Å low-pass filtered maps of the corresponding 3D classes as new initial models.

Crystallization, X-ray data collection and structure determination of the *Y. lipolytica* F_1c_{10} subcomplex. Purified 1*YL*F_1F_o at a protein concentration of 15 mg/ml was supplemented with 1.0% (w/v) 3-([3-cholamidopropyl]-dimethylammonio)-2-hydroxy-1-propanesulfonate (CHAPSO, Hampton Research, USA). 1 µl of the protein solution was then mixed with 1 µl of 100 mM Tris-HCl with a pH adjusted to 8.0 and 23% (v/v) of PEG 400 (Sigma-Aldrich, USA). Crystallization plates were incubated at 18°C. Cuboid crystals were obtained after 14 days. The crystals were harvested and cryo-cooled in liquid nitrogen after dipping into cryo-protectant solution containing reservoir solution supplemented with 15% (v/v) glycerol. Datasets were collected at 100 K at the beamline PX-II X10SA (Swiss Light Source, Villigen, CH). Diffraction data were indexed and integrated with XDS and scaled with XSCALE (Kabsch, 1993). For data processing statistics see **Table 1**.

The structure was determined by molecular replacement with PHASER (McCoy, 2007) using a search model created by SWISS-MODEL (Biasini et al., 2014) (all-atom rmsd to final model: 1.36 Å) based on the *S. cerevisiae* F_1c_{10} complex (PDB: 2XOK) (Stock et al., 1999). The *c*-ring was subsequently exchanged by a high-resolution yeast model

(PDB: 4F4S) and mutated manually to the *Y. lipolytica* sequence. Following an initial Autobuild run, iterative cycles of manual model building and refinement were performed with COOT (Emsley et al., 2010) and PHENIX (Afonine et al., 2012) to final R_{work}/R_{free} values of 0.274/0.305 at 3.50 Å resolution. The quality of the model was validated with MolProbity (Chen et al., 2010). Refinement and validation statistics are summarized in **Table 1**. The refinement resulted in unambiguous electron density maps for all subunits [α_E 49-533, α_{TP} 49-428 and 435-533, α_{DP} 49-533, β_E 39-506, β_{TP} 37-506, β_{DP} 37-506, γ23-116 and 120-292, δ15-94 and 101-133, ε16-residue poly-Ala model, and *c*1-76 (1 subunit), *c*1-75 (2 subunits), *c*2-76 (2 subunits), *c*2-75 (3 subunits), *c*2-74 (1 subunit), or *c*2-73 (1 subunit). Ramachandran plots indicated excellent stereochemistry with 94.94% of the non-glycine and non-proline residues found in the most-favored region and 0.15% outliers, as determined by Molprobity (Chen et al., 2010).

Structural alignments and rmsd calculations. Structures were superposed using the align function in PyMOL (The PyMOL Molecular Graphics System, Schrödinger, LLC.), which performs a sequence alignment before superposition. RMSD values were calculated for backbone atoms only to avoid bias from sequence similarity. Rotational states were analyzed by superposing structures on their entire γ-subunits (chain G), to optimize the fit over all atoms, before comparing the positions of the P-loops of the β_{DP} sites (chain D, residues 160-173 for *S. cerevisiae*, 159-172 for *Bos taurus* and 191-204 for *Y. lipolytica* structures).

Figures were generated with PyMOL (The PyMOL Molecular Graphics System, Schrödinger, LLC.) and USCF Chimera (Pettersen et al., 2004). The amino acid alignment figures were created using ESPript (Robert and Gouet, 2014). Movies were prepared with MORPHINATOR (Karlsen and Bublitz, 2016) and Sequimago scripts http://www.macupdate.com/app/mac/28164/sequimago.

Crosslink analysis - consistent crosslink distances. To validate the assignment of helix densities in the *Y. lipolytica* F_o stator subcomplex to subunits *a, b, 8, f* and *d*, we compared our model to crosslink distances reported for the F_o complexes of the closely related yeast *S. cerevisiae* or of *E. coli* (DeLeon-Rangel et al., 2013; Jiang and

Fillingame, 1998; Schwem and Fillingame, 2006; Stephens et al., 2003) (**Figure S6** and **Table S3**). Note that the crosslinks described for *E. coli* ATP synthase (Jiang and Fillingame, 1998) are also found in the *I. tartaricus* Na^+-ATP synthase (Vorburger et al., 2008). Another recently described crosslink (Lee et al., 2015) between subunits *e* and *f* also supports our subunit assignment. Sequence alignments to identify homologous residue positions in the *Y. lipolytica* dimer model closely matched all reported Cys-substitution crosslinks with the exception of residue *c*Leu53 of the c_{10} ring. Residues Val111 and Val112 of subunit *a* could not be assigned since both are located in the loop between *a*H3 and *a*H4. This loop is not resolved in our map, but is expected to be in close proximity to Ser55 of subunit *b* (**Figure S5**).

Supplementary References

Abrahams, J.P., Buchanan, S.K., Van Raaij, M.J., Fearnley, I.M., Leslie, A.G.W., and Walker, J.E. (1996). The structure of bovine F_1-ATPase complexed with the peptide antibiotic efrapeptin. Proc Natl Acad Sci USA *93*, 9420-9424.

Abrahams, J.P., Leslie, A.G.W., Lutter, R., and Walker, J.E. (1994). Structure at 2.8 Å resolution of F_1-ATPase from bovine heart mitochondria. Nature *370*, 621-628.

Afonine, P.V., Grosse-Kunstleve, R.W., Echols, N., Headd, J.J., Moriarty, N.W., Mustyakimov, M., Terwilliger, T.C., Urzhumtsev, A., Zwart, P.H., and Adams, P.D. (2012). Towards automated crystallographic structure refinement with phenix.refine. Acta Crystallogr D Biol Crystallogr *68*, 352-367.

Arsenieva, D., Symersky, J., Wang, Y., Pagadala, V., and Mueller, D.M. (2010). Crystal structures of mutant forms of the yeast F_1 ATPase reveal two modes of uncoupling. J Biol Chem *285*, 36561-36569.

Bason, J.V., Montgomery, M.G., Leslie, A.G.W., and Walker, J.E. (2014). Pathway of binding of the intrinsically disordered mitochondrial inhibitor protein to F_1-ATPase. Proc Natl Acad Sci USA *111*, 11305-11310.

Bason, J.V., Montgomery, M.G., Leslie, A.G.W., and Walker, J.E. (2015). How release of phosphate from mammalian F_1-ATPase generates a rotary substep. Proc Natl Acad Sci USA *112*, 6009-6014.

Biasini, M., Bienert, S., Waterhouse, A., Arnold, K., Studer, G., Schmidt, T., Kiefer, F., Cassarino, T.G., Bertoni, M., Bordoli, L., *et al.* (2014). SWISS-MODEL: modelling protein tertiary and quaternary structure using evolutionary information. Nucleic Acids Res *42*, W252-258.

Bowler, M.W., Montgomery, M.G., Leslie, A.G.W., and Walker, J.E. (2006). How azide inhibits ATP hydrolysis by the F-ATPases. Proc Natl Acad Sci USA *103*, 8646-8649.

Bowler, M.W., Montgomery, M.G., Leslie, A.G.W., and Walker, J.E. (2007). Ground state structure of F_1-ATPase from bovine heart mitochondria at 1.9 Å resolution. J Biol Chem *282*, 14238-14242.

Boyer, P.D. (1997). The ATP synthase - a splendid molecular machine. Annu Rev Biochem *66*, 717-749.

Braig, K., Menz, R.I., Montgomery, M.G., Leslie, A.G.W., and Walker, J.E. (2000). Structure of bovine mitochondrial F_1-ATPase inhibited by Mg^{2+} ADP and aluminium fluoride. Structure *8*, 567-573.

Cain, B.D. (2000). Mutagenic analysis of the F_0 stator subunits. J Bioenerg Biomembr *32*, 365-3671.

Chen, V.B., Arendall, W.B. 3rd, Headd, J.J., Keedy, D.A., Immormino, R.M., Kapral, G.J., Murray, L.W., Richardson, J.S., and Richardson, D.C. (2010). MolProbity: all-atom structure validation for macromolecular crystallography. Acta Crystallogr D Biol Crystallogr *66*, 12-21.

Cook, G.M., Keis, S., Morgan, H.W., von Ballmoos, C., Matthey, U., Kaim, G., and Dimroth, P. (2003). Purification and biochemical characterization of the F_1F_o-ATP synthase from thermoalkaliphilic *Bacillus* sp. strain TA2.A1. J Bacteriol *185*, 4442-4449.

Dautant, A., Velours, J., and Giraud, M.F. (2010). Crystal structure of the Mg.ADP-inhibited state of the yeast F_1c_{10}-ATP synthase. J Biol Chem *285*, 29502-29510.

Davies, K.M., Anselmi, C., Wittig, I., Faraldo-Gómez, J.D., and Kühlbrandt, W. (2012). Structure of the yeast F_1F_o-ATP synthase dimer and its role in shaping the mitochondrial cristae. Proc Natl Acad Sci USA *109*, 13602-13607.

DeLeon-Rangel, J., Ishmukhametov, R.R., Jiang, W., Fillingame, R.H., and Vik, S.B. (2013). Interactions between subunits *a* and *b* in the rotary ATP synthase as determined by cross-linking. FEBS Lett *587*, 892-897.

Emsley, P., Lohkamp, B., Scott, W.G., and Cowtan, K. (2010). Features and development of Coot. Acta Crystallogr D Biol Crystallogr *66*, 486-501.

Eya, S., Maeda, M., and Futai, M. (1991). Role of the carboxy terminal region of H^+-ATPase (F_0F_1) a subunit from *Escherichia coli*. Arch Biochem Biophys *284*, 71-77.

Giraud, M.F., Paumard, P., Sanchez, C., Brethes, D., Velours, J., and Dautant, A. (2012). Rotor architecture in the yeast and bovine F_1-c-ring complexes of F-ATP synthase. J Struct Biol *177*, 490-497.

Gledhill, J.R., Montgomery, M.G., Leslie, A.G.W., and Walker, J.E. (2007). How the regulatory protein, IF1, inhibits F_1-ATPase from bovine mitochondria. Proc Natl Acad Sci USA *104*, 15671-15676.

Hunte, C., Zickermann, V., and Brandt, U. (2010). Functional modules and structural basis of conformational coupling in mitochondrial complex I. Science *329*, 448-451.

Jiang, W., and Fillingame, R.H. (1998). Interacting helical faces of subunits *a* and *c* in the F_1F_0 ATP synthase of *Escherichia coli* defined by disulfide cross-linking. Proc Natl Acad Sci USA *95*, 6607-6612.

Kabaleeswaran, V., Puri, N., Walker, J.E., Leslie, A.G.W., and Mueller, D.M. (2006). Novel features of the rotary catalytic mechanism revealed in the structure of yeast F_1 ATPase. EMBO J *25*, 5433-5442.

Kabaleeswaran, V., Shen, H., Symersky, J., Walker, J.E., Leslie, A.G.W., and Mueller, D.M. (2009). Asymmetric structure of the yeast F_1 ATPase in the absence of bound nucleotides. J Biol Chem *284*, 10546-10551.

Kabsch, W. (1993). Automatic processing of rotation diffraction data from crystals of initially unknown symmetry and cell constants. J Appl Cryst *26*, 795-800.

Kagawa, R., Montgomery, M.G., Braig, K., Leslie, A.G.W., and Walker, J.E. (2004). The structure of bovine F_1-ATPase inhibited by ADP and beryllium fluoride. EMBO J *23*, 2734-2744.

Karlsen, J.L., and Bublitz, M. (2016). How to compare, analyze and morph between crystal structures of different conformations: the P-type ATPase example. Met Mol Biol *1377*, 523-539.

Lee, J., Ding, S., Walpole, T.B., Holding, A.N., Montgomery, M.G., Fearnley, I.M., and Walker, J.E. (2015). Organization of subunits in the membrane domain of the bovine F-ATPase revealed by covalent cross-linking. J Biol Chem *290*, 13308-13320.

Li, X., Mooney, P., Zheng, S., Booth, C.R., Braunfeld, M.B., Gubbens, S., Agard, D.A., and Cheng, Y. (2013). Electron counting and beam-induced motion correction enable near-atomic-resolution single-particle cryo-EM. Nat Methods *10*, 584-590.

Lightowlers, R.N., Howitt, S.M., Hatch, L., Gibson, F., and Cox, G.B. (1987). The proton pore in *Escherichia coli* F_0F_1- ATPase: A requirement of arginine at position 210 of the a-subunit. Biochim Biophys Acta *894*, 399-406.

Liu, S., Charlesworth, T.J., Bason, J.V., Montgomery, M.G., Harbour, M.E., Fearnley, I.M., and Walker, J.E. (2015). The purification and characterization of ATP synthase complexes from the mitochondria of four fungal species. Biochem J *468*, 167-175.

Ludtke, S.J., Baldwin, P.R., and Chiu, W. (1999). EMAN: semiautomated software for high-resolution single-particle reconstructions. J Struct Biol *128*, 82-97.

McCoy, A.J. (2007). Solving structures of protein complexes by molecular replacement with Phaser. Acta Crystallogr D Biol Crystallogr *63*, 32-41.

Menz, R.I., Leslie, A.G.W., and Walker, J.E. (2001a). The structure and nucleotide occupancy of bovine mitochondrial F_1-ATPase are not influenced by crystallisation at high concentrations of nucleotide. FEBS Lett *494*, 11-14.

Menz, R.I., Walker, J.E., and Leslie, A.G.W. (2001b). Structure of bovine mitochondrial F_1-ATPase with nucleotide bound to all three catalytic sites: implications for the mechanism of rotary catalysis. Cell *106*, 331-341.

Mindell, J.A., and Grigorieff, N. (2003). Accurate determination of local defocus and specimen tilt in electron microscopy. J Struct Biol *142*, 334-347.

Morales-Rios, E., Montgomery, M.G., Leslie, A.G.W., and Walker, J.E. (2015). Structure of ATP synthase from *Paracoccus denitrificans* determined by X-ray crystallography at 4.0 Å resolution. Proc Natl Acad Sci USA *112*, 13231-13236.

Orriss, G.L., Leslie, A.G.W., Braig, K., and Walker, J.E. (1998). Bovine F_1-ATPase covalently inhibited with 4-chloro-7-nitrobenzofurazan: the structure provides further support for a rotary catalytic mechanism. Structure *6*, 831-837.

Pettersen, E.F., Goddard, T.D., Huang, C.C., Couch, G.S., Greenblatt, D.M., Meng, E.C., and Ferrin, T.E. (2004). UCSF Chimera--a visualization system for exploratory research and analysis. J Comput Chem *25*, 1605-1612.

Rees, D.M., Leslie, A.G.W., and Walker, J.E. (2009). The structure of the membrane extrinsic region of bovine ATP synthase. Proc Natl Acad Sci USA *106*, 21597-21601.

Rees, D.M., Montgomery, M.G., Leslie, A.G.W., and Walker, J.E. (2012). Structural evidence of a new catalytic intermediate in the pathway of ATP hydrolysis by F_1-ATPase from bovine heart mitochondria. Proc Natl Acad Sci USA *109*, 11139-11143.

Robert, X., and Gouet, P. (2014). Deciphering key features in protein structures with the new ENDscript server. Nucleic Acids Res *42*, W320-324.

Robinson, G.C., Bason, J.V., Montgomery, M.G., Fearnley, I.M., Mueller, D.M., Leslie, A.G.W., and Walker, J.E. (2013). The structure of F_1-ATPase from *Saccharomyces cerevisiae* inhibited by its regulatory protein IF1. Open Biol *3*, 120164.

Schägger, H., and von Jagow, G. (1987). Tricine-sodium dodecyl sulfate-polyacrylamide gel electrophoresis for the separation of proteins in the range from 1 to 100 kDa. Anal Biochem *166*, 368-379.

Scheres, S.H. (2012). RELION: implementation of a Bayesian approach to cryo-EM structure determination. J Struct Biol *180*, 519-530.

Scheres, S.H. (2014). Beam-induced motion correction for sub-megadalton cryo-EM particles. eLife *3*, e03665.

Schwem, B.E., and Fillingame, R.H. (2006). Cross-linking between helices within subunit *a* of *Escherichia coli* ATP synthase defines the transmembrane packing of a four-helix bundle. J Biol Chem *281*, 37861-37867.

Stephens, A.N., Khan, M.A., Roucou, X., Nagley, P., and Devenish, R.J. (2003). The molecular neighborhood of subunit *8* of yeast mitochondrial F_1F_0-ATP synthase probed by cysteine scanning mutagenesis and chemical modification. J Biol Chem *278*, 17867-17875.

Stock, D., Leslie, A.G.W., and Walker, J.E. (1999). Molecular architecture of the rotary motor in ATP synthase. Science *286*, 1700-1705.

Tang, G., Peng, L., Baldwin, P.R., Mann, D.S., Jiang, W., Rees, I., and Ludtke, S.J. (2007). EMAN2: an extensible image processing suite for electron microscopy. J Struct Biol *157*, 38-46.

van Raaij, M.J., Abrahams, J.P., Leslie, A.G.W., and Walker, J.E. (1996). The structure of bovine F_1-ATPase complexed with the antibiotic inhibitor aurovertin B. Proc Natl Acad Sci USA *93*, 6913-6917.

Vorburger, T., Ebneter, J.Z., Wiedenmann, A., Morger, D., Weber, G., Diederichs, K., Dimroth, P., and von Ballmoos, C. (2008). Arginine-induced conformational change in the *c*-ring/*a*-subunit interface of ATP synthase. FEBS J *275*, 2137-2150.

Watt, I.N., Montgomery, M.G., Runswick, M.J., Leslie, A.G.W., and Walker, J.E. (2010). Bioenergetic cost of making an adenosine triphosphate molecule in animal mitochondria. Proc Natl Acad Sci USA *107*, 16823-16827.

Wittig, I., Braun, H.P., and Schägger, H. (2006). Blue native PAGE. Nat Protoc *1*, 418-428.

Zhou, A., Rohou, A., Schep, D.G., Bason, J.V., Montgomery, M.G., Walker, J.E., Grigorieff, N., and Rubinstein, J.L. (2015). Structure and conformational states of the bovine mitochondrial ATP synthase by cryo-EM. eLife *4*.
